# Emerging Trends in Cross-Coupling: Twelve-Electron-Based L_1_Pd(0) Catalysts, Their Mechanism of Action, and Selected Applications

**DOI:** 10.1021/acs.chemrev.2c00204

**Published:** 2022-10-03

**Authors:** Sharbil J. Firsan, Vilvanathan Sivakumar, Thomas J. Colacot

**Affiliations:** † Science and Lab Solutions−Chemistry, MilliporeSigma, 6000 North Teutonia Avenue, Milwaukee, Wisconsin53209, United States; ‡ Merck Life Science Pvt Ltd, No-12, Bommasandra-Jigani Link Road, Industrial Area, Bangalore560100, India; § Science and Lab Solutions−Chemistry, MilliporeSigma, 6000 North Teutonia Avenue, Milwaukee, Wisconsin53209, United States

## Abstract

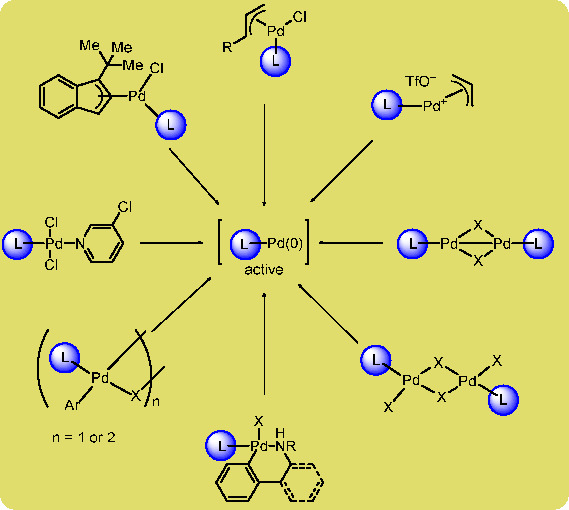

Monoligated palladium(0) species, L_1_Pd(0), have emerged as the most active catalytic species in the cross-coupling cycle. Today, there are methods available to generate the highly active but unstable L_1_Pd(0) catalysts from stable precatalysts. While the size of the ligand plays an important role in the formation of L_1_Pd(0) during in situ catalysis, the latter can be precisely generated from the precatalyst by various technologies. Computational, kinetic, and experimental studies indicate that all three steps in the catalytic cycle—oxidative addition, transmetalation, and reductive elimination—contain monoligated Pd. The synthesis of precatalysts, their mode of activation, application studies in model systems, as well as in industry are discussed. Ligand parametrization and AI based data science can potentially help predict the facile formation of L_1_Pd(0) species.

## 1 Introduction

The palladium-catalyzed cross-coupling is an exceptionally important area within the field of homogeneous catalysis in modern organic synthesis. This is attested to by the awarding of the 2010 Nobel Prize in chemistry to Richard Heck, Akira Suzuki, and Ei-ichi Negishi, three pioneers in the 1970s of different types of Pd catalyzed carbon-carbon cross-coupling reactions that bear their names.^1^ Other pioneers, such as Murahashi, Stille, and Mizoroki, have also made fundamental contributions that, along with those of Hiyama, Tamao, and Miyaura, have made it possible for the technology to develop and mature into what it is today. The significant discoveries and applications of the different types of cross-coupling, which have propelled these reactions to become a leading synthesis technology of the 21st century, have been reviewed by Colacot, Snieckus, and coauthors.^2^ Cross-coupling is an example of a technology where incremental rather than breakthrough innovations led to the major advances in the field and, consequently, the awarding of the Nobel Prize, with a focus on C–C bond forming cross-coupling reactions. Since the mid-1990s, independent pioneering work by Hartwig and Buchwald has expanded the scope of the cross-coupling reaction to carbon–heteroatom bond-forming reactions, known today collectively as the Buchwald–Hartwig cross-coupling, which has seen so far a tremendous growth in its applications.

Until the late 1990s, the improvement and expansion of cross-coupling reactions had focused on: (i) switching from Ni to Pd in order to utilize the well-defined, two-electron process,^3^ and (ii) changing the nucleophilic coupling partner from an organomagnesium nucleophile to an organozinc, tin, silicon, or boron guided by Pauling’s electronegativity scale.^4^ One of the milestones in the development of cross-coupling was disclosed by Littke and Fu in 1998,^5^ in which the effective coupling of an aryl chloride in a C–C coupling was achieved using P(*t-*Bu)_3_ in conjunction with a palladium catalyst precursor.^6^ In the same year, Koie reported the use of a P(*t*-Bu)_3_/Pd system for the amination of aryl chlorides with diarylamines.^7,8^ Concurrently with the disclosure of Littke and Fu, reports from Buchwald’s^9^ and Hartwig’s^10^ laboratories also helped to significantly propel this area of research to a whole new level with the introduction of novel privileged ligands, newer applications, and better processes.^11^ N-Heterocyclic carbenes (NHCs),^12^ which had been investigated by Herrmann^13,14^ and co-workers in conjunction with palladium, also emerged as a new class of ligand for cross-coupling, with significant successive contributions by Nolan,^15−20^ Organ,^21,22^ and Glorius.^23^ A consensus then emerged that designed ligands with appropriate steric bulk and electronic parameters were the most important component in cross-coupling,^11^ an observation that had also been implied earlier independently by Osborn^24^ and Milstein^25^ for the palladium-catalyzed carbonylations of aryl chlorides. Key observations led to important conclusions regarding the relationship of ligand properties to its overall effect on the catalytic cycle: oxidative addition, transmetalation, and reductive elimination.^26^

**Figure 1:**
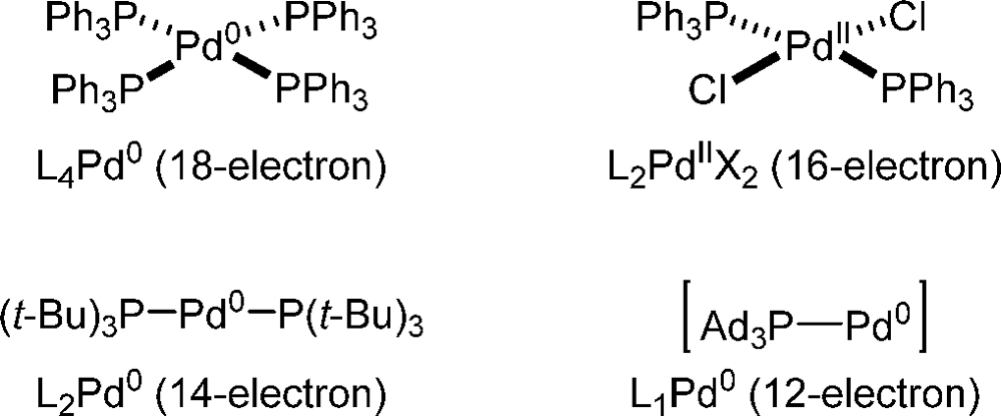
Examples of 18-, 16-, 14-, and 12-electron Pd species.

Although monoligated L_1_Pd(0) complexes constitute the active species in the cross-coupling catalytic cycle, these complexes are practically impossible to synthesize and isolate due to their high reactivity. Hence, precursors such as Pd_2_(dba)_3_ and Pd(OAc)_2_ have been employed in conjunction with suitable ligands to generate the “active” Pd(0) in situ. The inherent purity issues of both Pd_2_(dba)_3_ and Pd(OAc)_2_ have been highlighted^27,28^ based on the work of Ananikov^29^ and Colacot,^30,31^ respectively. When one uses in situ catalysis (ligand plus a Pd precursor), the precise formation of the desired catalytic species is difficult to achieve, although the size and geometry of the ligand may help to a certain extent. Hence, the in situ technology can result in poor efficiency of the overall catalytic process in terms of metal loading, conversion, and selectivity. It is well understood that even when utilizing the same ligand, there could be a significant difference in activity between monoligated and bisligated complexes (Figure 1).^32^ As far as the preformed catalysts are concerned, the 14-electron-based L_2_Pd(0) complexes are typically utilized directly or generated in situ from a Pd precursor, L_2_PdX_2_.^33^ Although many examples of bis-coordinated L_2_Pd(0) complexes are commercially available, even in bulk quantities,^34^ monoligated L_1_Pd(0) complexes have yet to be isolated or fully characterized as stated above. The high activity of monoligated L_1_Pd(0) is related to its unsaturated coordination sphere, based on the well-known 18-electron rule postulated by Langmuir.^35,36^

Fortunately, several new technologies do exist today that permit the generation of the air-sensitive and highly reactive L_1_Pd(0) species through activation of suitable precatalysts. Many of these new-generation precatalysts are air- and moisture-stable, even at elevated temperatures and in solution, due to their existence as Pd(II) complexes. The rate and mode of activation of these precatalysts are important in determining the outcome of catalytic transformations. Therefore, these unique precatalysts, containing the same ligand, may exhibit different cross-coupling activities and selectivities, depending on their structural profile and reaction conditions.

Currently, there are no comprehensive reviews on this relevant modern topic, and this survey is intended to help researchers both in academia and industry gain a better understanding of this important emerging area.

In the sections that follow, this review highlights the development of various emerging technologies for generating L_1_Pd(0) catalysts, wherein L is a tertiary phosphine or NHC ligand. Each of the approaches for generating L_1_Pd(0) is described and critically evaluated in terms of synthesis, activation mechanism, and unique applications. Overlap with earlier reviews has been avoided; however, relevant reviews from the last 5–10 years are cited to make this review comprehensive. Selected industrial applications focusing on pharmaceuticals are also included. Most importantly, this review should serve as a primer for chemists wanting to become familiar with cross-coupling reactions and as a reference guide for chemists seeking alternative synthetic methods that offer improved reaction efficiency from a process- and atom-economy points of view.

## 2 Mechanistic Studies Suggesting the Involvement of L_1_Pd(0) in the Catalytic Cycle

**Scheme 1:**
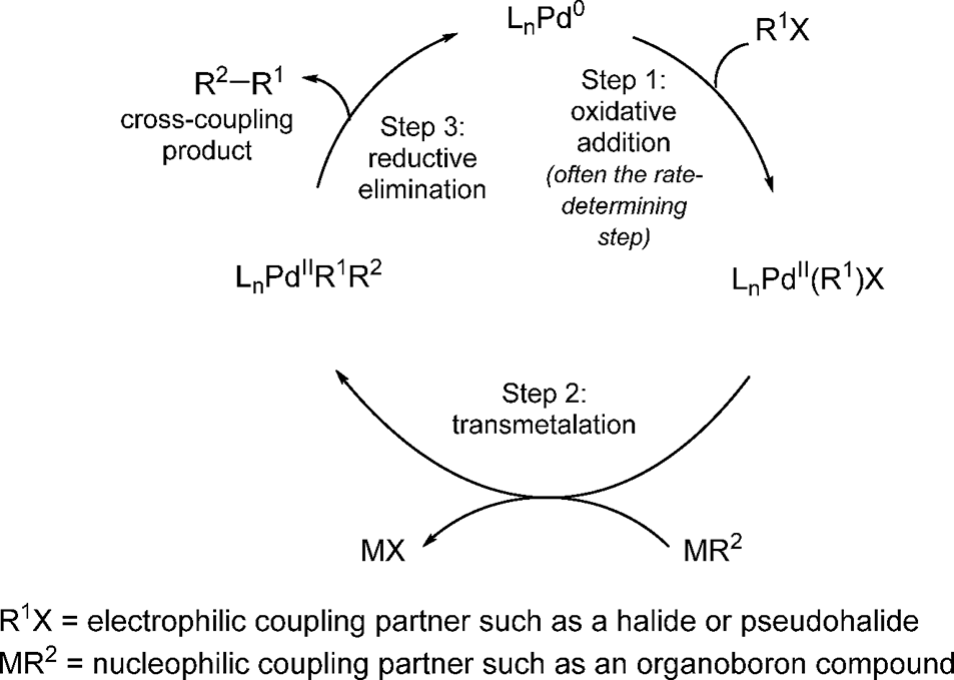
Major Steps in the Generally Accepted Catalytic Cycle of Cross-Coupling Reactions

The generally accepted mechanism of cross-coupling reactions involves three principal steps: (i) oxidative addition, (ii) transmetalation, and (iii) reductive elimination (Scheme 1).^37^ Miyaura and Suzuki’s statement,^37^ “*palladium complexes that contain fewer than four phosphine ligands or bulky phosphines such as tris(2,4,6-trimethoxyphenyl)phosphine are, in general, highly reactive for the oxidative addition because of the ready formation of coordinate unsaturated palladium species*”, is based on a kinetic study conducted by Farina and Krishnan on the Stille cross-coupling reaction.^38^ Farina’s observation that excess phosphine slows down the coupling, coupled with the above statement by Miyaura and Suzuki, may have prompted many modern cross-coupling experts to look into employing bulky ligands to form coordinatively unsaturated palladium complexes to improve the efficacy and efficiency of a given cross-coupling reaction.

In this regard, investigations carried out by Fu on P(*t*-Bu)_3_, Buchwald on biaryl ligand systems, Hartwig on the QPhos ligand, and Beller on CataCXium (Evonik Degussa GmbH) ligands have clearly indicated that bulky phosphine ligands prefer forming low-coordination Pd species during the catalytic cycle.^11,39^ In general, Tolman’s cone angle has been useful in measuring the overall sterics of the phosphine ligand^11^ while Nolan’s^15−20^ and Organ’s^21,22^ work on bulky N-heterocyclic carbenes (NHCs) have invoked the percent buried volume (% *V*_bur_) for ligand parametrization, especially for NHC systems.

One of the fundamental questions in cross-coupling reactions is about the nature of the active species in the catalytic cycle. While it is generally accepted that L_*n*_Pd(0) is the active species, the further question is whether or not it is a monocoordinated (*n* = 1) or biscoordinated (*n* = 2) palladium or both. In this section, we shall review the available mechanistic studies aimed at answering these questions.

### 2.1 L_1_Pd(0)-Based Catalytic Species in the Oxidative Addition Step

**Scheme 2:**
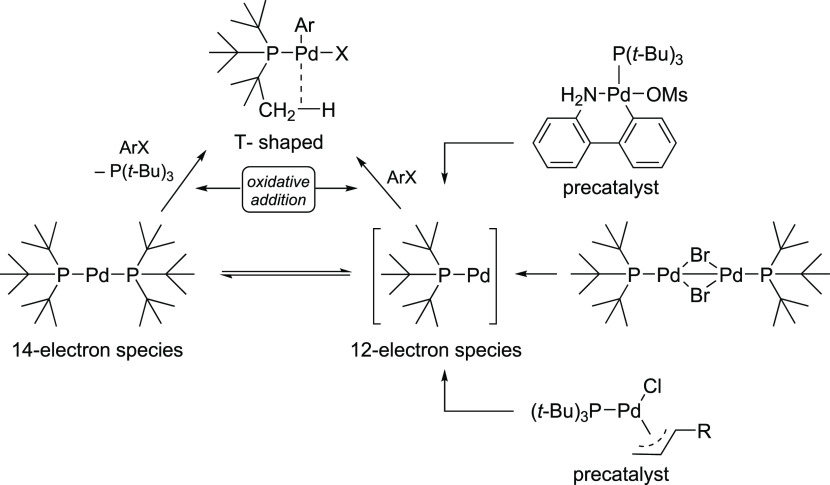
Formation of Monoligated, T-Shaped L_1_Pd(Ar)X Species during the Oxidative Addition Step from L_2_Pd(0) and from L_1_Pd-Based Precatalysts with a Bulky Ligand such as P(*t*-Bu)_3_

Bulky ligands such as P(*t*-Bu)_2_(1-Ad) or P(*t*-Bu)_3_, with a cone angle of ca. 180°, are known to form both monoligated and bisligated complexes. However, based on kinetic and mechanistic studies by Hartwig and co-workers,^40,41^ even the isolable L_2_Pd(0) initially loses one of the ligands to form a monomeric oxidative addition complex, L_1_Pd(Ar)X, possessing a T-shaped geometry (Scheme 2).^40−42^ This T-shaped intermediate is typically stabilized by a weak agostic interaction between a C–H in the ligand L and the fourth coordination site of the palladium.

In certain cases, the oxidative addition product can be a monoligated dimer, [L_1_Pd(Ar)X]_2_, depending on the nature of the ligand L (e.g., L = P(*o*-Tol)_3_) and the nature of the aryl halide, ArX. Kinetic studies by Hartwig and Paul on the oxidative addition of aryl bromides to Pd[P(*o*-Tol)_3_]_2_ indicated an inverse first-order dependence of the observed rate constants on the concentration of P(*o*-Tol)_3_.^43^ This is in contrast to the classic understanding of the oxidative addition of ArI to Pd(PPh_3_)_4_, where dissociation of two PPh_3_ ligands leads to a 14-electron intermediate, Pd(PPh_3_)_2_, which then produces a four-coordinate (Ph_3_P)_2_Pd(Ar)I species.^44,45^

By using ion-trap mass spectrometry, McIndoe, Maseras, and co-workers demonstrated that, in the Pd(PPh_3_)_4_ catalyzed oxidative addition of bromobenzene, the reactivity ratio of bromobenzene with L_1_Pd(0) vs L_2_Pd(0) was at least 10^4^:1 by mass spectrometric measurements and 10^5^:1 by theoretical calculations.^46^ A computational (DFT) study by Norrby and co-workers on the oxidative addition of aryl chlorides to monoligated Pd complexes revealed that electron deficient aryl chlorides tend to interact strongly with Pd due to back-donation to form stronger prereactive complexes.^47^ Further DFT studies, combined with polarized continuum solvation models, carried out by Fu, Liu, and co-workers, revealed that for PhX substrates (X = Cl and Br), the transition state of the oxidative addition to 14-electron-based [Pd(PPh_3_)_2_] has a much higher free energy than the transition state of the 12-electron-based [Pd(PPh_3_)] species.^48^ Noticeably for the bulky P(*t*-Bu)_3_, the L_2_Pd transition state does not even exist. Hence, for both bulky P(*t*-Bu)_3_ and less bulky PPh_3_ ligands, oxidative additions seem to proceed via a 12-electron-based L_1_Pd(0) pathway with PhX (X = Cl and Br).

**Scheme 3:**
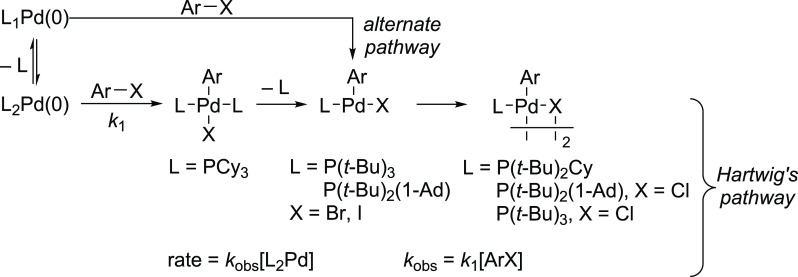
Hartwig’s Observations for the Direct Oxidative Addition of ArX to L_2_Pd(0)

Systematic studies by Hartwig and co-workers on the oxidative addition of Ar–I to a series of trialkylphosphine–palladium complexes having the general formula L_2_Pd, where L = P(*t*-Bu)_3_, P(*t*-Bu)_2_Cy, P(*t*-Bu)(Cy)_2_, and PCy_3_, indicated that bulky ligands such as P(*t*-Bu)_3_ and P(*t*-Bu)_2_Cy form the L_1_Pd(Ar)I species by ligand dissociation. In contrast, Pd complexes with the relatively less bulky P(*t*-Bu)(Cy)_2_ and PCy_3_ ligands give L_2_Pd(Ar)I by an associative pathway (Scheme 3).^49,50^ These findings are in agreement with those of Brown and co-workers.^51^ Further computational studies conducted by Harvey, Fey, and co-workers using dispersion-corrected DFT together with solvation models have corroborated Hartwig’s findings.^52^ The oxidative addition products of PhBr and PhCl are also known to form the stable, four-coordinate trans complexes just as in the case of the iodide.^24,42,53^

**Scheme 4:**
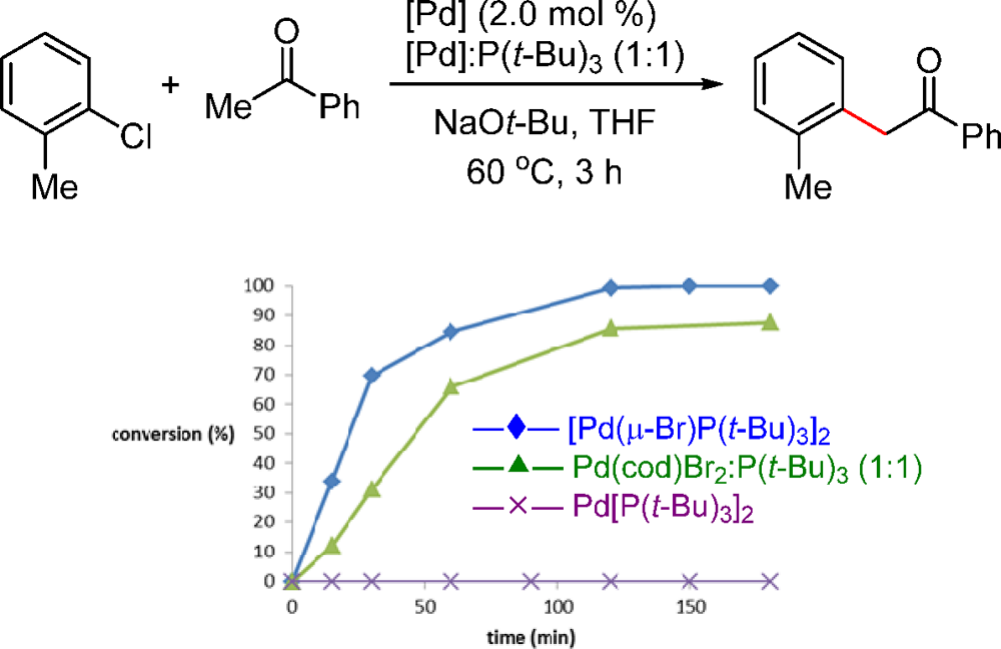
Activity Difference between Mono- vs Bis-Ligated Pd-Based P(*t*-Bu)_3_Complexes Adapted with Permission from ref (32). Copyright 2017 American Chemical Society.

These studies concluded that less bulky ligands, such as PPh_3_ and PCy_3_, tend to form L_2_Pd(Ar)X, while bulkier ones, such as P(*t*-Bu)_2_(1-Ad) and P(*t*-Bu)_3_, form L_1_Pd(Ar)X even from L_2_Pd(0). Although Hartwig’s pathway in Scheme 3 indicates that ligand dissociation takes place after oxidative addition, for bulkier ligands it could occur prior to oxidative addition, leading to equilibration beteeen L_2_Pd(0) and [L_1_Pd(0)]. Similar observations were made by Shaughnessy with Np-based L_2_Pd(0) complexes, which gave [LPd(Ar)(μ-X)]_2_ dimers upon reaction with ArX.^54,55^ Very bulky ligands, for example, some biaryl ligands such as *t*-BuBrettPhos, with very large cone angle may not form L_2_Pd(0); however, these ligands might form L_1_Pd(Ar)X during the oxidative addition even when the Pd complex is generated in situ. Nevertheless, kinetic studies by Colacot and co-workers point to a significant difference in activity between preformed monocoordinated and biscoordinated P(*t*-Bu)_3_ complexes of Pd (Scheme 4).^32^

**Scheme 5:**
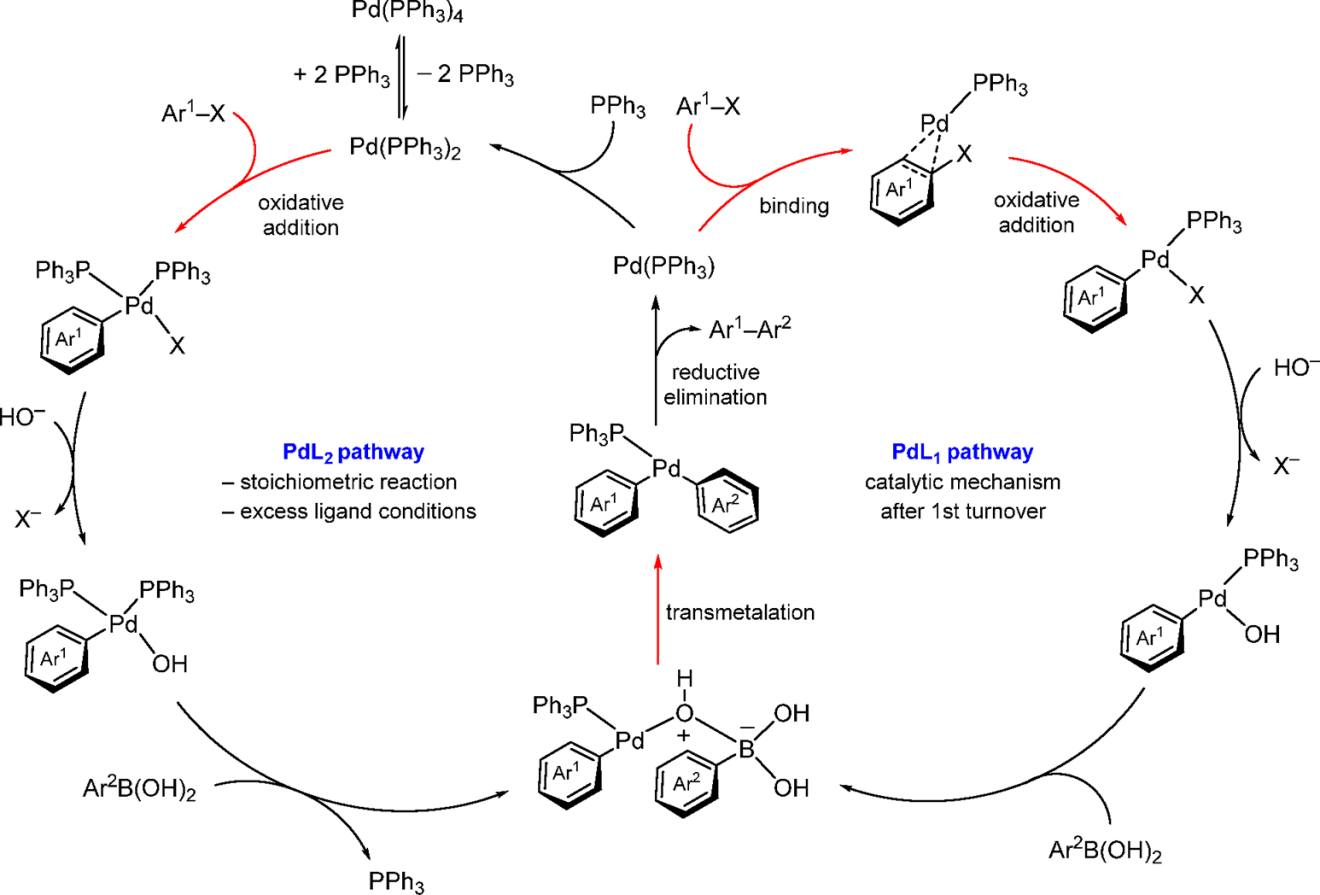
Herschi and Vetticatt’s Proposed Catalytic Cycle for the Suzuki–Miyaura Reaction with the Key OA and TM Steps in the Catalytic Cycle That Are Validated by This Study Highlighted in Red Adapted with permission from ref (56). Copyright 2022 American Chemical Society.

Very recently, Hirschi, Vetticatt, and co-workers carried out a combined study of theoretical and experimental ^13^C kinetic isotope effects to gain an understanding of the mechanism of the Pd(PPh_3_)_4_ catalyzed Suzuki–Miyaura cross-coupling of aryl halides with aryl boronic acids, where oxidative addition of the aryl halide takes place onto a 12-electron, monoligated palladium complex, [Ph_3_P–Pd]. The study revealed that the commonly proposed oxidative addition to the 14-electron Pd(PPh_3_)_2_ complex can happen only under stoichiometric conditions or in the presence of excess added ligand. However, after the first turnover and in the absence of excess ligand, the catalytically active species is the 12-electron, monoligated [Ph_3_P–Pd] (Scheme 5).^56^

### 2.2 Does Reductive Elimination in the Catalytic Cycle Involve L_1_Pd(Ar)(Nu), Where Nu = Nucleophile?

Several studies have been conducted on the reductive elimination step to form C–C bonds with palladium complexes containing bidentate phosphines and monophosphines with sterics similar to that of PPh_3_. It was found that reductive elimination is faster from three-coordinate than from four-coordinate complexes in Pd systems with sterically bulky ligands.^57−59^ The rates of reductive elimination from palladium complexes containing two monophosphine ligands is inversely dependent on the concentration of the added ligand, suggesting that reductive elimination occurs via a three-coordinate species by losing one of the ligands.^60,61^

**Scheme 6:**
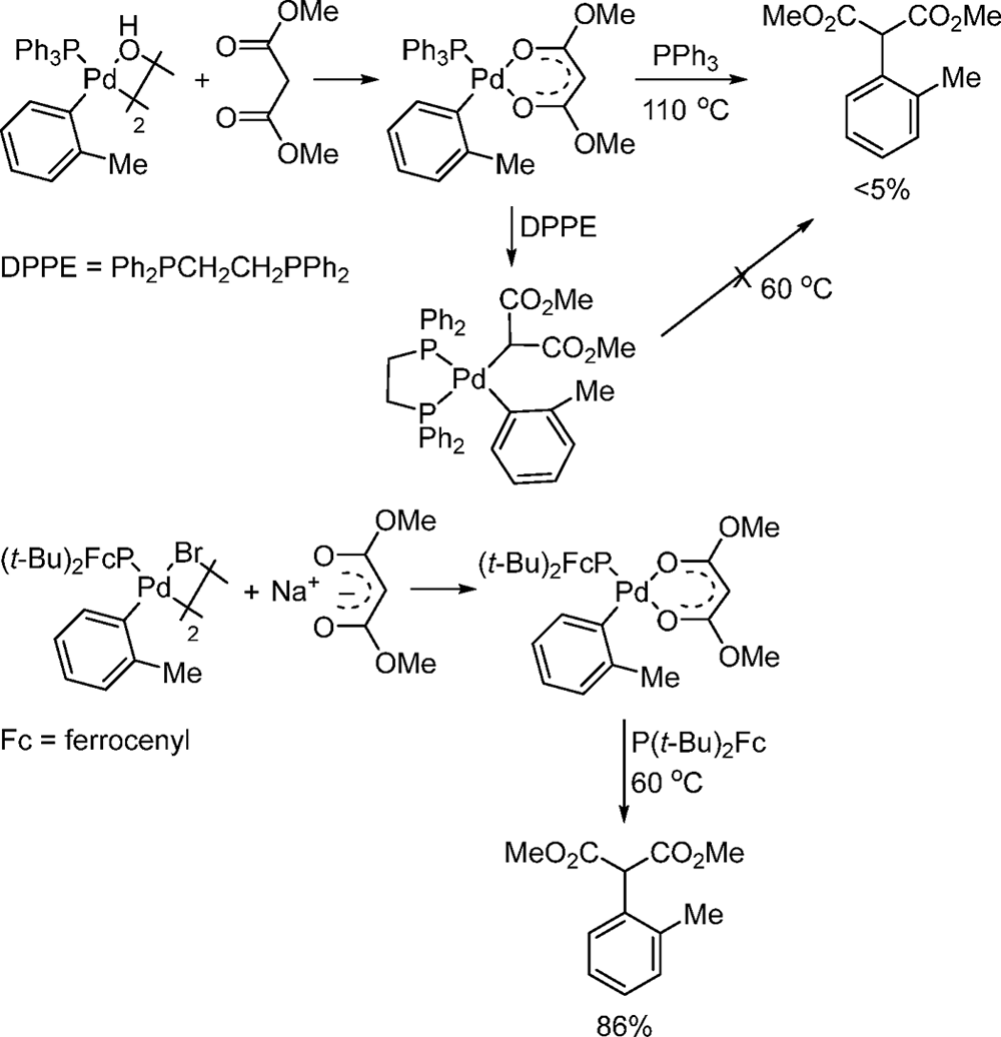
Ease of Reductive Elimination of Palladium–Malonate Complexes, Demonstrating the Effect of Bulky Ligands via L_1_Pd(Ar)(malonate)

Hartwig’s efforts to develop palladium-catalyzed α-arylations of carbonyl compounds and nitriles based on the reductive eliminations of C(sp^2^)–C(sp^3^) bonds have been reviewed.^62^ Reductive elimination from arylpalladium malonate ligated by a bulky alkylphosphine such as P(*t*-Bu)_2_Fc (Fc = ferrocenyl) occurred in high yield and at lower temperatures than from complexes of less bulky ligands such as PPh_3_ or bisphosphines. The faster reductive elimination from complexes of P(*t*-Bu)_2_Fc over those ligated by PPh_3_ or DPPE is attributed to the increased steric bulk of the alkylphosphine in the tricoordinate species (Scheme 6).^62^

**Scheme 7:**
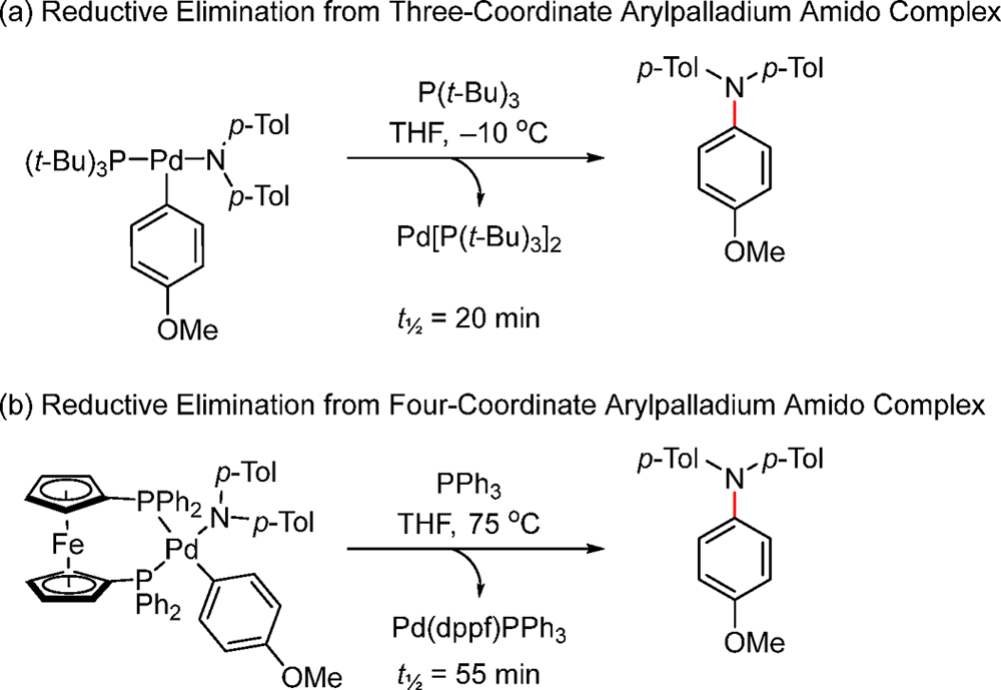
Relative Ease of the Reductive Elimination from Three- vs Four-Coordinate Arylpalladium Amido Complexes of the Type LPd(Ar)(NAr_2_), Where L = P(*t*-Bu)_3_ or dppf

Similar trends were observed for the C–N coupling as well. Yamashita and Hartwig reported the first examples of monomeric three-coordinate arylpalladium amido complexes and investigated the reductive elimination of arylamines from these species.^41^ The reactions of potassium arylamides with three-coordinate (oxidative addition) arylpalladium halide complexes, L_1_Pd(Ar)(Br) [L = P(*t*-Bu)_3_, QPhos, and P(*t*-Bu)_2_Fc], formed the corresponding three-coordinate arylpalladium amido complexes as stable species at room temperature when the aryl and amido ligands bore highly deactivating groups. Upon thermolysis, these complexes underwent reductive elimination to form arylamines. For complexes substituted with the bulkier phosphines, the yield of the coupled product increased in the order: P(*t*-Bu)_3_ > QPhos > P(*t*-Bu)_2_Fc. The bulky P(*t*-Bu)_3_ based three-coordinate Pd amido complex undergoes reductive elimination with a *t*_1/2_ of 20 min even at −10 °C, while the dppf-based four-coordinate amido complex undergoes the same but at a temperature of 75 °C and a *t*_1/2_ of 55 min, once again showing the preference of monoligated, tricoordinate Pd for facile elimination in the catalytic cycle (Scheme 7).^41^

**Scheme 8:**
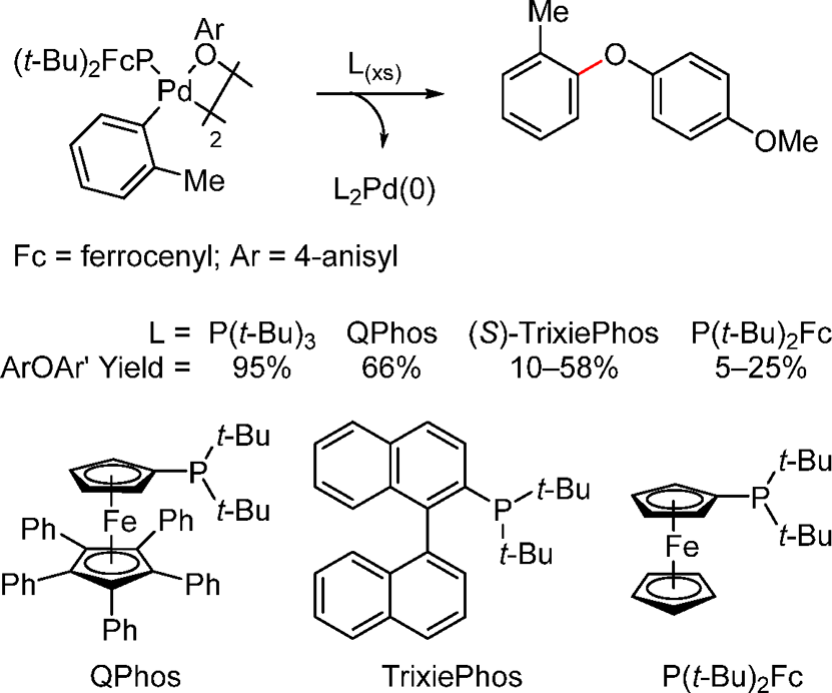
Ligand Effect on the Reductive Elimination from [L_1_Pd(Ar)(OAr)]_2_ Leading to Diaryl Ethers

To our knowledge, reductive elimination from arylpalladium thiolato complexes ligated by bulky monophosphine ligands has not been reported.^63^ However, for C–O coupling reactions, Hartwig found that the yields of diaryl ether formed by reductive elimination from isolated arylpalladium aryloxo complexes can be significantly increased by addition of bulky alkylphosphine ligands. The conversion to diaryl ether was highest in the presence of the bulkiest ligand (Scheme 8).^64^

In summary, palladium complexes ligated by bulky alkylphosphines are favorable for both the oxidative addition and reductive elimination steps in cross-coupling reactions via monoligated three-coordinate species.

### 2.3 The Role of Monoligated Pd in the Transmetalation Step

As discussed above, both the oxidative addition^65−67^ and reductive elimination^37,68−71^ steps have been studied in detail and are generally applicable to all cross-coupling reactions. However, the transmetalation steps are the least understood, as name reactions such as Murahashi (organo-Li), Kumada–Corriu (organo-Mg), Negishi (organo-Zn), Stille (organo-Sn), Hiyama–Denmark (organo-Si), and Suzuki–Miyaura (organo-B) involve different kinds of organometallic reagents (nucleophiles), where the intricate transfer of the organic moiety to Pd might differ from one metal to another. As early as 1983 and based on a limited study, Labadie and Stille provided some rationale for the transmetalation in the Stille coupling reaction.^72^

**Scheme 9:**
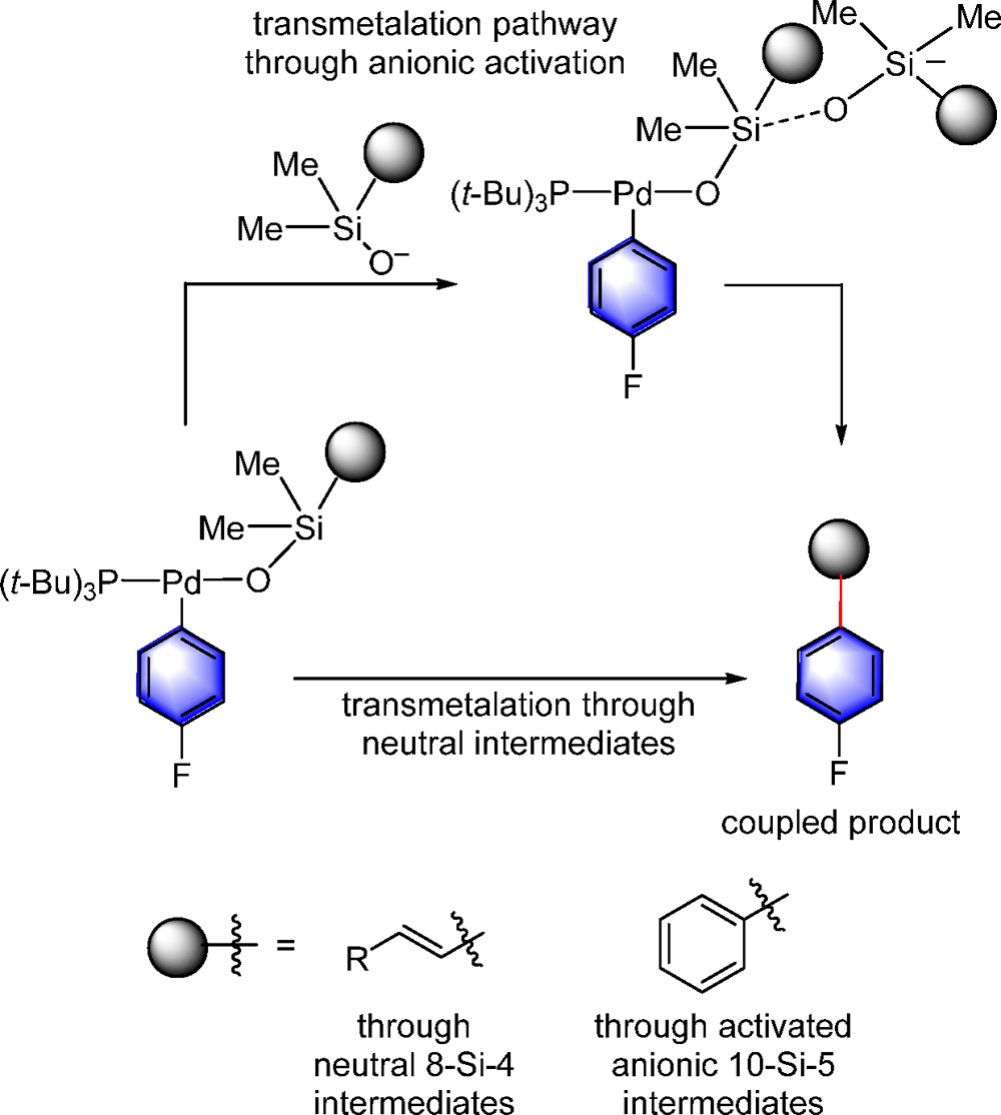
Denmark’s Identification of the Monoligated Three-Coordinate T-Shaped L_1_Pd Silanolate in the Silicon-to-Palladium Transmetalation Step in the Hiyama–Denmark Coupling

More recently, Denmark conducted comprehensive investigations of the transmetalation step in the Hiyama–Denmark coupling.^73,74^ These studies conclusively demonstrated that, in the cross-coupling with silanolate salts, two mechanistic regimes are operating, both of which involve a discrete Si–O–Pd linked intermediate in which the palladium atom is three-coordinate.^73,74^ In one pathway, a neutral, four-coordinate silicon unit (8-Si-4)^75^ undergoes intramolecular transmetalation, whereas in the second pathway, an anionic, five-coordinate silicon unit (10-Si-5) undergoes intramolecular transmetalation as well albeit at a significantly faster rate (Scheme 9).^73,74^ The demonstration that silanolate cross-couplings proceed by intramolecular transmetalation of discrete Si–O–Pd intermediates motivated the Denmark group to investigate whether the related Suzuki–Miyaura cross-coupling proceeds by a related mechanism (vide infra). The dominance of the Suzuki–Miyaura reaction in industrial applications provided additional motivation for these studies.^76,77^

Denmark’s studies sought to provide a fundamental understanding of the transfer of the organic fragment from boron to palladium and were enabled by low-temperature and rapid injection NMR spectroscopic analysis (RI-NMR), combined with a series of structural, kinetic, and computational (DFT) investigations. Prior to Denmark’s investigations to identify the “missing link” in the transmetalation step, the research groups of Suzuki and Miyaura,^78−80^ Soderquist,^81^ Amatore and Jutand,^82,83^ Schmidt,^84^ and Hartwig^85^ had provided justifications for one of two pathways, path A (“boronate”) and path B (“oxo-palladium”) prior to transmetalation (vide infra). Soderquist’s study gave indirect evidence for the Pd–O–B linkage, while Hartwig’s kinetic study revealed that path B is favored over path A kinetically by more than 4 orders of magnitude.^85^ However, the displacement of bromide with hydroxide from the oxidative addition complex has been found to have a transition-state barrier of 18.6 kcal/mol, which suggests that path A is more favorable than path B.^86−89^ This area has been thoroughly reviewed by Lennox and Lloyd-Jones.^90^ For PPh_3_-ligated complexes, Maseras, Ujaque, and co-workers carried out calculations on the displacement of bromide from the oxidative addition product of trihydroxyphenylboronate and palladium hydroxide complex and concluded that both pathways are capable of forming Pd–O–B linkages; however, this study had its limitations.^91^ Harvey and co-workers have also studied computationally the effect of the size of the ligand on the transmetalation step by considering ligands of various electronic and steric properties, such as P(*t*-Bu)_3_, P(CF_3_)_3_, PMe_3_, and PPh_3_, and found that the effect of steric bulk was twice as important as that of the electronic parameters.^92^

**Scheme 10:**
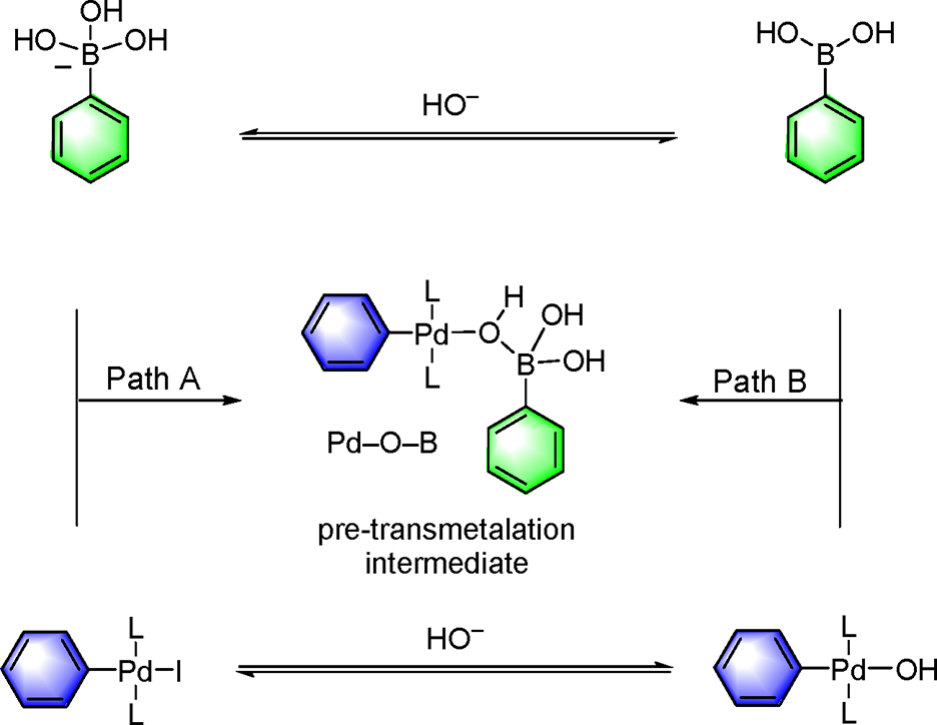
Two Known Transmetalation Pathways Involving “Boronate” (Path A) vs “Oxo-Palladium” (Path B) in the Suzuki–Miyaura Cross-Coupling

Denmark’s group has clearly shown that, in the reaction of *trans*-[P(*i*-Pr)_3_]_2_(4-FC_6_H_4_)Pd(OH) with ArB(OH)_2_ vs [P(*i*-Pr)_3_]_2_(4-FC_6_H_4_)Pd(I) with [ArB(OH)_3_]^−^, path B (oxo-palladium) is preferred over path A (boronate) when ArB(OH)_2_ is used as the nucleophile (Scheme 10).^93^ However, when boronate esters are employed instead of boronic acids, the “boronate” mechanism is also possible as the reaction takes place under anhydrous conditions.^94^

**Scheme 11:**
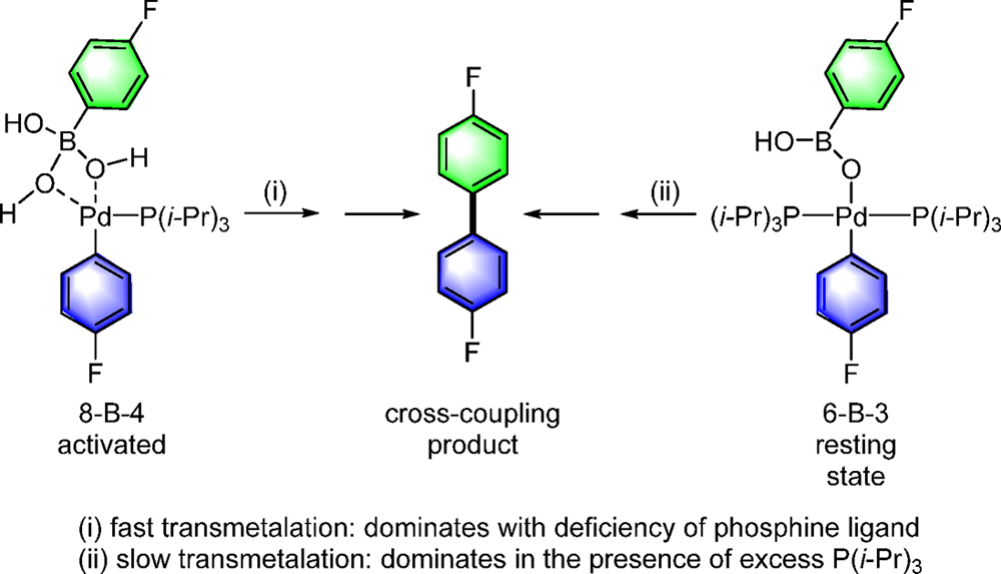
Denmark’s Proposed “Missing Links” 6-B-3 and 8-B-4 of the Transmetalation Step in the Suzuki–Miyaura Cross-Coupling

The primary conclusion from Denmark’s studies is that the Suzuki–Miyaura cross-coupling does indeed proceed via discrete B–O–Pd containing intermediates just as in the Hiyama–Denmark reaction. Here again, two pathways were identified in which both tri- (6-B-3) and tetracoordinate (8-B-4) Pd complexes serve as pretransmetalation intermediates with the latter again undergoing more rapid transmetalation (Scheme 11).^93,95^

**Scheme 12:**
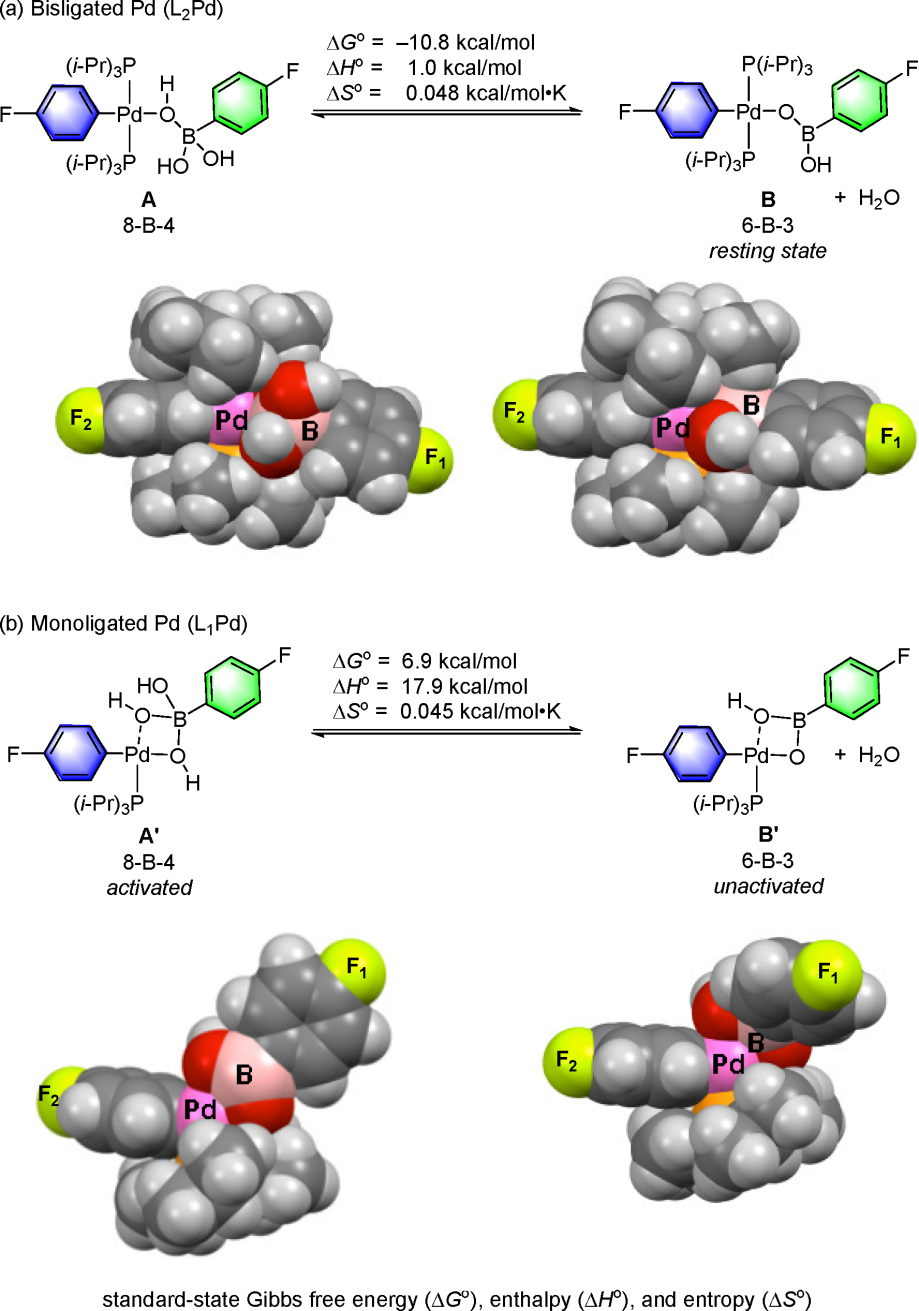
Calculated Equilibria for Loss of Water at 30 °C from 8-B-4 Pre-Transmetalation Intermediates (Bisligated A vs Monoligated A′) Adapted with permission from ref (93). Copyright 2017 American Chemical Society.

A key reason for the slower transmetalation of the 6-B-3 intermediate is that it exists as a diligated palladium species (and the kinetic equation for the transmetalation shows an inverse first-order dependence on the phosphine ligand, P(*i*-Pr)_3_). Calculated ground-state equilibrium energies for L_1_Pd and L_2_Pd based 8-B-4 complexes provided insight into the instability of the 8-B-4 complex during its synthesis with two ligands on Pd. The loss of water from an initially formed 8-B-4 complex (**A**), yielding 6-B-3 complex **B**, was found to be highly exergonic (Scheme 12).^93^ Space-filling models clearly show that L_2_Pd(II) intermediate 6-B-3 (**B**) prevents the binding of the water to access species 8-B-4 as the OH groups on boron penetrate the van der Waals radii of the isopropyl methyl groups on phosphorus, thereby destabilizing the four-coordinate geometry. This was substantiated by the failed attempt to isolate 8-B-4 (**A**) from 6-B-3 (**B**) by addition of water or base.^93^

This behavior illustrates the point that the steric bulk of the two P(*i-*Pr)_3_ groups on the palladium in **A** prevents the formation of the requisite 8-B-4 species for rapid transmetalation. Removal of a phosphine ligand from the complex results in a monoligated complex (**A′**). Calculation of the ground-state energies of monoligated complexes **A′** and **B′** indicated a reversal of the position of the equilibrium, substantially favoring the 8-B-4 species (**A′**) with a monoligated Pd. This insight was crucial for focusing research efforts on the study of monoligated arylpalladium(II) complexes in order to form the long-sought-after 8-B-4 activated intermediate.

**Scheme 13:**
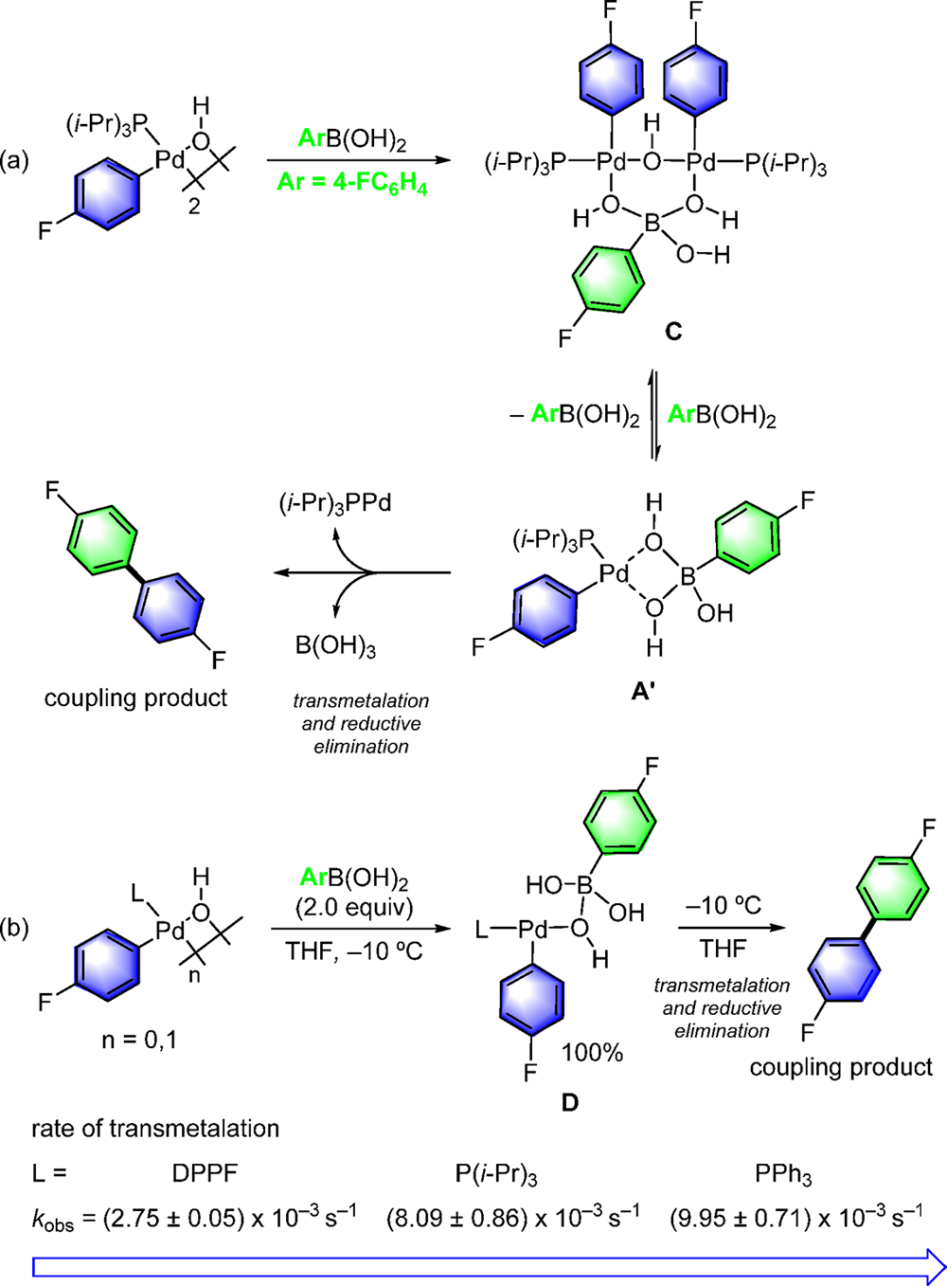
(a) Stepwise Synthesis of Pre-transmetalation (A′) and Transmetalation Intermediate (D) from [L_1_Pd(Ar)(OH)]_2_ (L = P(*i*-Pr)_3_); (b) Rate of Transmetalation of [L_1_Pd(Ar)(OH)]_2_ (L = dppf, PPh_3_, and P(*i*-Pr)_3_) Indicating Monoligation, Although dppf Is Slower Than the Monodentate Ligands

Denmark also provided experimental evidence for the formation of a dimeric palladium complex, **C**, from the monoligated precursor [(*i*-Pr)_3_PPd(4-FC_6_H_4_)(OH)]_2_ by mixing the latter with one equivalent of boronic acid (Scheme 13).^93^ This intermediate **C** was stable in the presence of additional boronic acid in THF. However, in THF–methanol solution, dimer **C** converted into the monomeric adduct in a 1:1 stoichiometry with ArB(OH)_2_ to give the long-sought-after monoligated 8-B-4 complex **A′** (see Scheme 11) as the pretransmetalation intermediate that is ready to undergo reductive elimination to the final coupled product.

This study also revealed that palladium complexes bearing various phosphine ligands such as PPh_3_, P(*i*-Pr)_3_, and DPPF also form Pd–O–B species as intermediates in cross-coupling reactions. The rate of the transmetalation is comparable for all ligands but decreases in the order Ph_3_*P* ≥ P(*i*-Pr)_3_ > DPPF, demonstrating a common mechanism involving a coordinatively unsaturated and electrophilic palladium atom during the transmetalation process.^93^

The ability to directly interrogate the transmetalation step kinetically enabled the investigation of the behavior of various boronic esters such as those of catechol and glycol.^94^ Interestingly, these boron reagents undergo direct transmetalation via similar transmetalation intermediates but with increased rates as compared to the arylboronic acids under anhydrous conditions. This behavior is distinct from the study by Lloyd-Jones and co-workers conducted in the presence of water with organoboron reagents such as trifluoroborates^90,96−98^ and MIDA boronates,^99^ wherein hydrolysis to the parent boronic acid is required prior to the transmetalation step. In fact, Denmark has recently described a preparatively advantageous method that employs neopentylboronate esters under anhydrous conditions to effect a broadly applicable Suzuki–Miyaura cross-coupling that obviated the need for protodeboronation.^100^

In summary, on the basis of comprehensive structural, kinetic, and computational investigations, it is clear that for both the Hiyama–Denmark and Suzuki–Miyaura cross-couplings, the transmetalation intermediate in the catalytic cycle is a monoligated L_1_Pd(II) species as opposed to the corresponding L_2_Pd(II) complex, irrespective of the size and coordination number of the palladium. The very recent study by Hirschi, Vetticatt, and co-workers^56^ employing experimental and theoretical ^13^C kinetic isotope effects showed that the transmetalation step likely proceeds via a tetracoordinate boronate (8-B-4) intermediate with a Pd–O–B linkage as already observed by Denmark. Having coordinatively unsaturated palladium is crucial for the rapid transfer of the organic group from silicon or boron to palladium.

## 3 Role of the Ligands in Forming the L_1_Pd(0) Precatalyst in Situ

One of the significant advances in cross-coupling over the last two decades was the development of new ligands that make the technology viable for organic synthesis in both academia and industry.^11,101−104^ The facile formation of the low-coordinate, monoligated Pd(0) species is the key factor in determining the efficiency and efficacy of the catalyst system. Until the late 1990s, the most commonly employed ligand for cross-coupling was PPh_3_, which was thought to form a 14-electron Pd(PPh_3_)_2_ complex as the active species during catalysis either in situ from Pd(OAc)_2_ or Pd_2_(dba)_3_ or from a precatalyst. However, recent computational^105^ and experimental^56,106^ studies invoke the involvement of monoligated [(Ph_3_P)Pd(0)] as the active species in the catalytic cycle. Based on ab initio studies, Vidossich et al. have proposed solvent stabilization of the monoligated [(Ph_3_P)Pd(0)] under catalytic conditions.^107^

The major breakthrough in this area happened when the known, pyrophoric, and least-explored ligand tri(*tertiary*-butyl)phosphine, P(*t*-Bu)_3_, was utilized independently by Fu^5,6,108^ and Koie^7,8^ in situ for aryl chloride C–C and C–N bond formation. Up until then, it was difficult to effectively couple an aryl chloride under palladium catalysis due to the large C–Cl bond dissociation energy.^109^ The unique role of P(*t*-Bu)_3_ in facilitating the cross-coupling was attributed to its steric bulkiness and electron richness, which are related to its cone angle and p*K*_a_, respectively.^11^ Today, P(*t*-Bu)_3_ is considered a privileged ligand in cross-coupling. Fu also used PCy_3_ in certain challenging cross-couplings, where PPh_3_ was not effective;^108^ however, P(*t*-Bu)_3_ was far superior for challenging coupling reactions, including those of unactivated aryl chlorides.^5^ In the same year, Buchwald reported a new type of biaryl ligand containing PCy_2_ (DavePhos) for the Suzuki–Miyaura cross-coupling of unactivated aryl chlorides^9^ as a modification to his BINAP systems, which had earlier been employed for the C–N coupling of aryl bromides.^110^ Interestingly, DavePhos clearly demonstrates the balancing role of the sterics and electronics of ligands in cross-couplings, as PCy_3_ alone cannot effect the analogous transformations for certain challenging substrates. If one considers steric factors alone, P(*o*-Tol)_3_, which has a larger cone angle than that of P(*t*-Bu)_3_, should facilitate aryl chloride couplings readily but does not. One year later, Buchwald incorporated the P(*t*-Bu)_2_ moiety into the biaryl scaffold to generate P(*t*-Bu)_2_R (R = biphenyl), known as JohnPhos, which allowed the room temperature amination of aryl chlorides to be carried out effectively.^111,112^ These findings were the impetus for developing a new class of biaryl ligands, commonly known as Buchwald ligands, with members such as SPhos, RuPhos, XPhos, *t*BuXPhos BrettPhos, *t*BuBrettPhos, AdBrettPhos, GPhos, and others. Today, these are some of the most privileged classes of commercially available ligands for cross-coupling.^102,113−115^

In 1998, Hartwig also created a new P(*t*-Bu)_2_R-type ligand called QPhos, in which R is a pentaphenylferrocene moiety.^10^ This is now a commercially available ligand that has proved superior in challenging coupling reactions. Beller also disclosed the bulky electron-rich ligand PAd_2_(*n*-Bu) in 2000,^116^ while Buchwald had reported a year earlier the synthesis of the P(*t*-Bu)_2_(biphenyl) for challenging cross-coupling reactions.^117^

**Figure 2:**
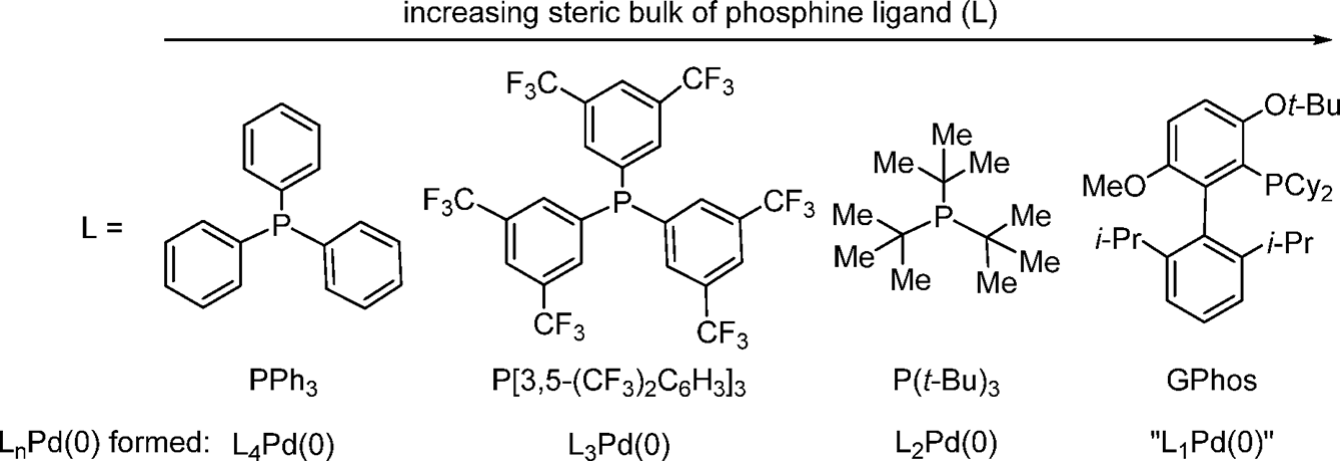
Effect of phosphine ligand size on the type of L_*n*_Pd(0) formed during in situ catalysis.

As the bulkiness of the ligand increases, the ligand tends to form coordinatively unsaturated Pd(0) species in the catalytic cycle, thereby increasing the oxidative addition and reductive elimination abilities, although the electronic properties of the ligand also play a key role in the oxidative addition step as we discussed above. The size of the ligand determines the “*n*” in L_*n*_Pd(0). That is why typically PPh_3_ forms L_4_Pd(0), while P[3,5-(CF_3_)_2_C_6_H_3_]_3_ forms L_3_Pd(0).^118^ The bulky P(*t*-Bu)_3_ forms L_2_Pd(0).^34^Figure 2 depicts the effect of ligand size on L*_n_*Pd(0) formation. As the steric bulk of the ligand increases further, as in BrettPhos, *t*-BuBrettPhos, GPhos, and others, the ligand tends to form “L_1_Pd(0)”, and this has been invoked as an intermediate by desorption electrospray ionization mass spectrometry under C–C and C–N bond-forming reaction conditions^119^ and by ESI MS under Suzuki–Miyaura coupling conditions.^120^

### 3.1 Computational Prediction of L_1_Pd vs L_2_Pd Formation in the Catalytic Cycle by Using Ligand Parameters

**Scheme 14:**
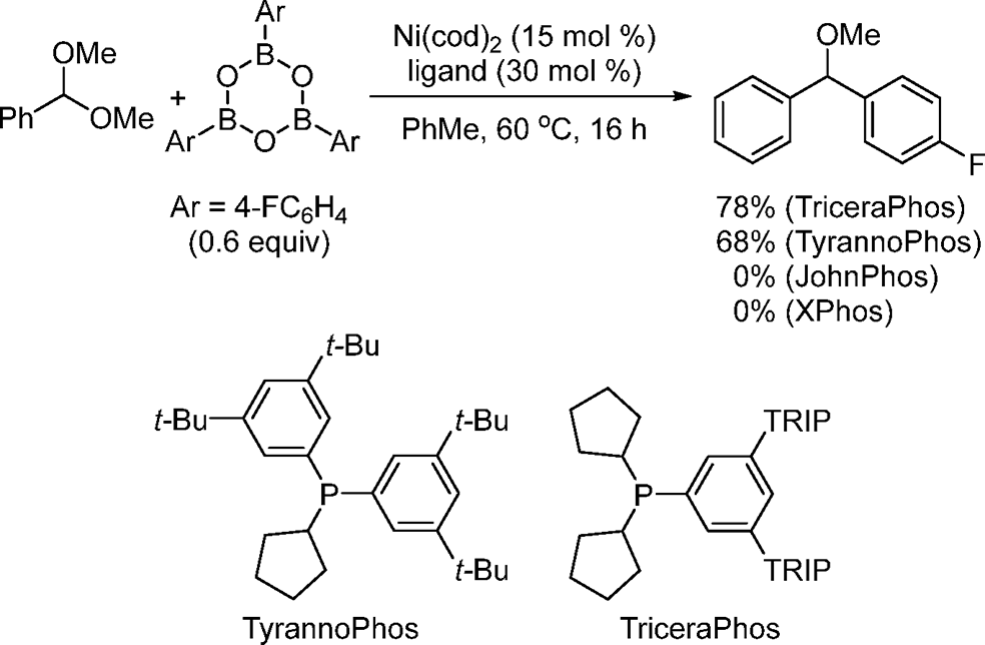
Reaction of an Acetal and a Boroxine Illustrating the Beneficial Remote Steric Effect of the DinoPhos Class of Ligands in the Ni-Catalyzed C_sp3_ Suzuki Coupling

**Scheme 15:**
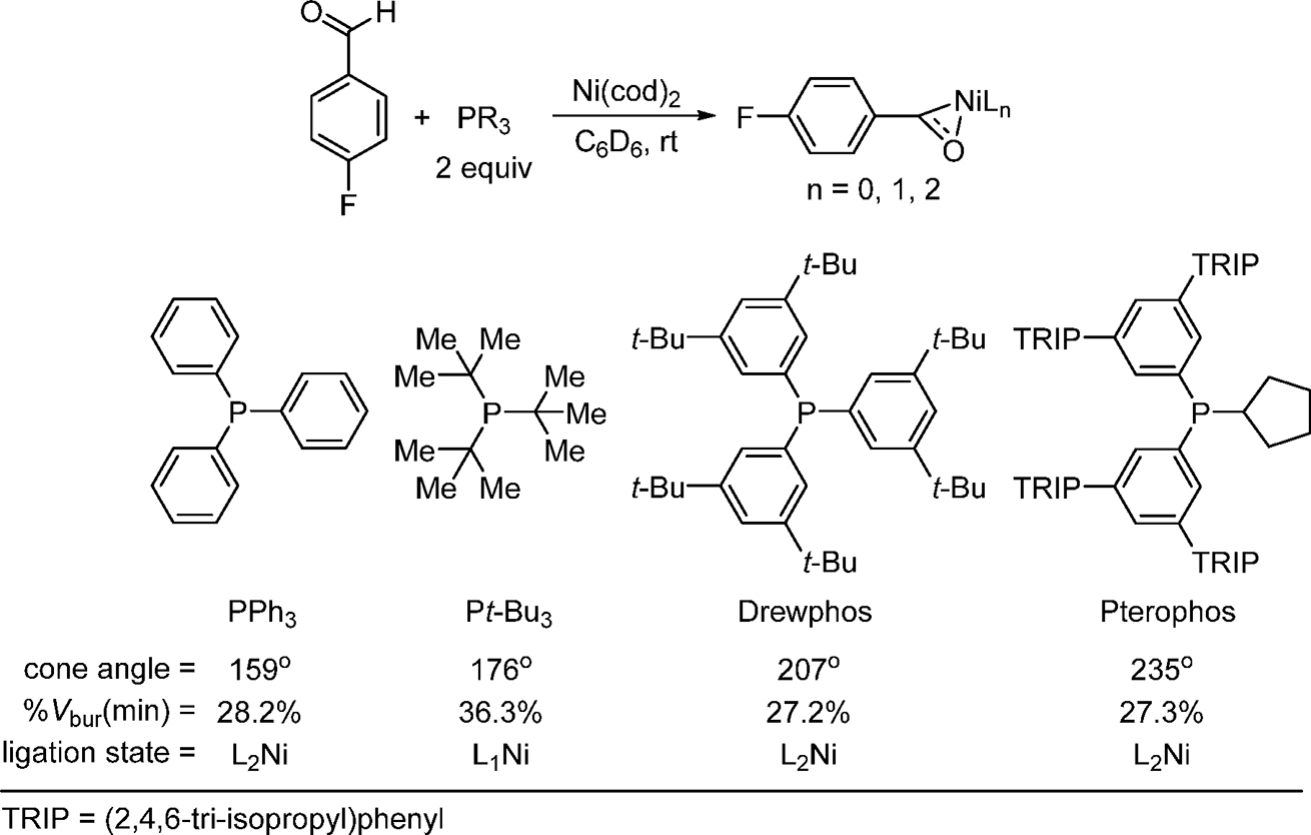
Descriptor Minimal Percent Buried Volume, %*V*_bur_(min), as a Ligation State Criterion

Ligand parametrization for predicting the outcome of a catalytic reaction with the help of data science is emerging as a new tool in organic synthesis.^121−123^ Although the Tolman cone angle^124,125^ descriptor has been of great value in understanding and predicting ligand properties in catalysis, it suffers from certain drawbacks, one of which is not taking ligand flexibility into consideration.^126−128^ Recently, the groups of Sigman and Doyle reported that the use of a single descriptor, minimum percent buried volume or %*V*_bur_(min), could nicely predict the ligation state of the catalytically active complexes in both Pd- and Ni-catalyzed reactions.^129^ Initially, the authors were inspired by the high efficiency of the DinoPhos ligand family (TyrannoPhos and TriceraPhos) in the Ni-catalyzed coupling reaction of acetal and boroxine (Scheme 14).^130^ Even though DinoPhos ligands feature large cone angles, similar to P(*t*-Bu)_3_, interestingly they have small %*V*_bur_, similar to PPh_3_. The authors studied the ligation state, which has been reported to influence the reaction outcome, of various ligands with 4-fluorobenzaldehyde (Scheme 15)^129^ and further discovered that the %*V*_bur_(min) descriptor would classify the ligands with a clear threshold. Any monodentate phosphine which has a %*V*_bur_(min) value below this threshold forms a bisligated complex and otherwise forms a monoligated complex. From a structural perspective, with the help of X-ray diffraction and DFT calculations, several L_*n*_Ni(4-fluorobenzaldehyde) (*n* = 1 or 2) structures were revealed. For example, although PteroPhos (see Scheme 15) has a large cone angle compared to P(*t*-Bu)_3_, it still forms the L_2_Ni(0) complex. As can be seen, cone angle does not reflect the overall topology of the ligand structure; however, %*V*_bur_(min) captures the flexibility of the steric bulk after the first ligand coordination.

**Scheme 16:**
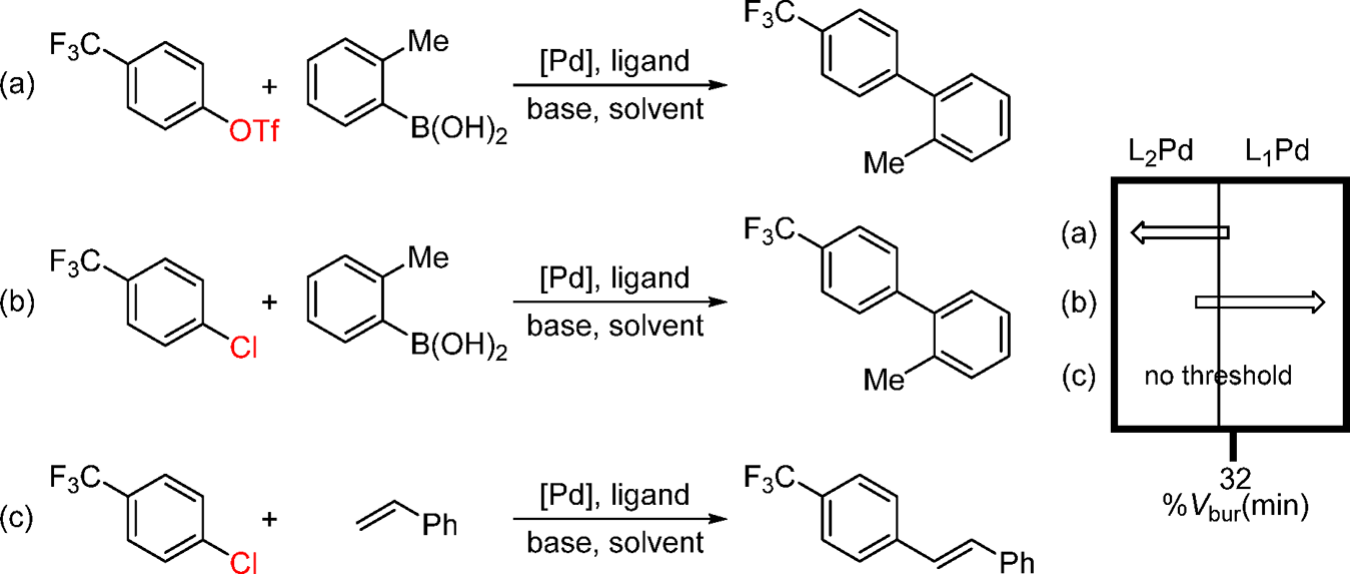
Determination of Ligation State with Descriptor %*V*_bur_(min) in Pd-Catalyzed Reactions

These studies indicated a possible region of %*V*_bur_ (min) values in which L_2_M is thermodynamically favored in the resting state, but L_1_M is also found in solution. Within this region, the equilibrium between L_2_M and L_1_M would be influenced by such factors as the temperature, solvent, and concentration of the reaction. The concept of %*V*_bur_(min) has also been successfully expanded into the Pd-catalyzed cross-coupling reactions. When plotting the reaction yield against the ligand descriptor %*V*_bur_(min), clear thresholds that separate active and inactive ligands can be obtained with the directionality of active and inactive regions determined by whether the active catalyst is L_1_Pd or L_2_Pd.^131,132^ In Scheme 16, part (a), L_2_Pd is identified as the catalytically active species, whereas L_1_Pd is identified as the catalytically active species in part (b). When the ligand sterics is not the decisive factor, e.g., Scheme 16, part (c), no threshold can be identified.^133^ The authors proposed that this approach should facilitate mechanistic studies of related organometallic reactions and enable reaction development by identifying active and inactive as well as mono- and bis-ligating phosphines before synthesis. Even though the authors acknowledge that %*V*_bur_ (min) may not capture reactivity trends across all phosphines, its ability to identify outliers (particularly false negatives) can spur the development of new descriptors and targeted mechanistic studies. Besides using a single descriptor to predict the ligation state of palladium, Schoenebeck and co-workers recently reported a compound descriptor method via an unsupervised machine learning process for predicting a rare situation of the palladium ligation state, Pd(I) dimer, rather than L_2_Pd.^134^

Various recent review articles and book chapters have listed some of the most sought-after phosphine and heterocyclic carbene ligands for cross-coupling applications. Colacot’s book chapter^11^ and Nolan’s book chapter^16^ provide an update on the phosphine and NHC ligands, respectively, up to 2014, while Shaughnessy’s^135^ and Hazari’s^103^ reviews highlight recent updates in this area.

## 4 Synthesis and Applications of L_1_Pd(0) Precatalysts

### 4.1 Introduction

As mentioned in the Introduction and in the section on mechanisms, it is clear that monoligated Pd(II) “T-shaped” intermediates are favored in all three steps of the catalytic cycle. The size of the ligand and the nature of the precatalyst play key roles in accelerating the formation of “L_1_Pd(0)” as the active catalytic species.

**Scheme 17:**
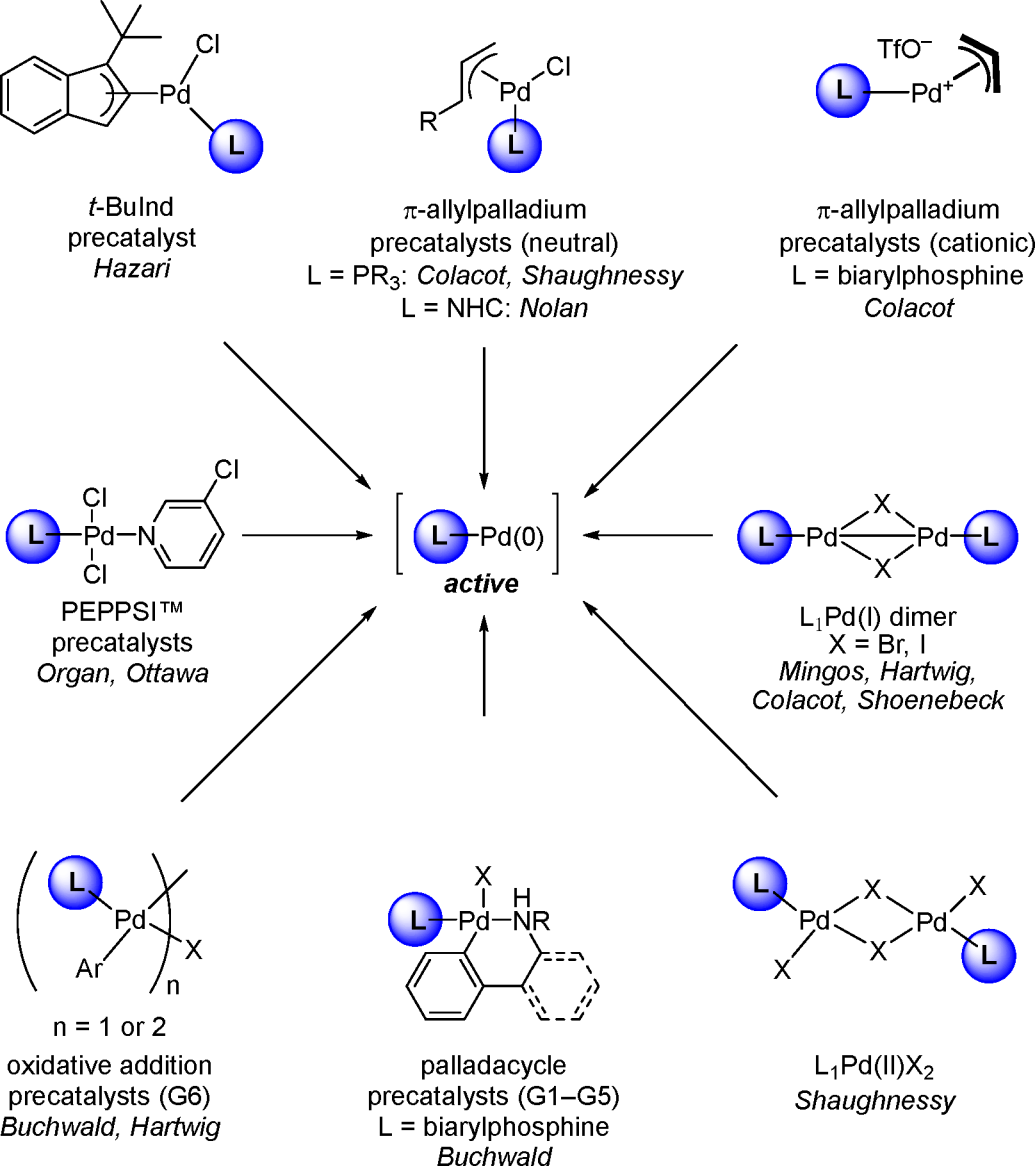
Active “L_1_Pd(0)” Can Be Generated from a Variety of Precatalysts

Although various precatalysts (Scheme 17) had been used to generate “L_1_Pd(0)”, its physical existence was not well-established until very recently. Once the coordinatively unsaturated monoligated “L_1_Pd(0)” is generated, it has to be trapped either as an oxidative addition complex, L_1_Pd(II)(Ar)(X) (see the section on mechanism), or be coordinated with a neutral ligand such as an olefin to keep it stable as a Pd(0) complex; otherwise, it can disintegrate into palladium black instantaneously. Vilar’s mini-review highlights the developments in this area until 2005;^136^ however, the majority of the precatalysts featured in Scheme 17 have appeared in the literature only in the past 5 years.

**Scheme 18:**
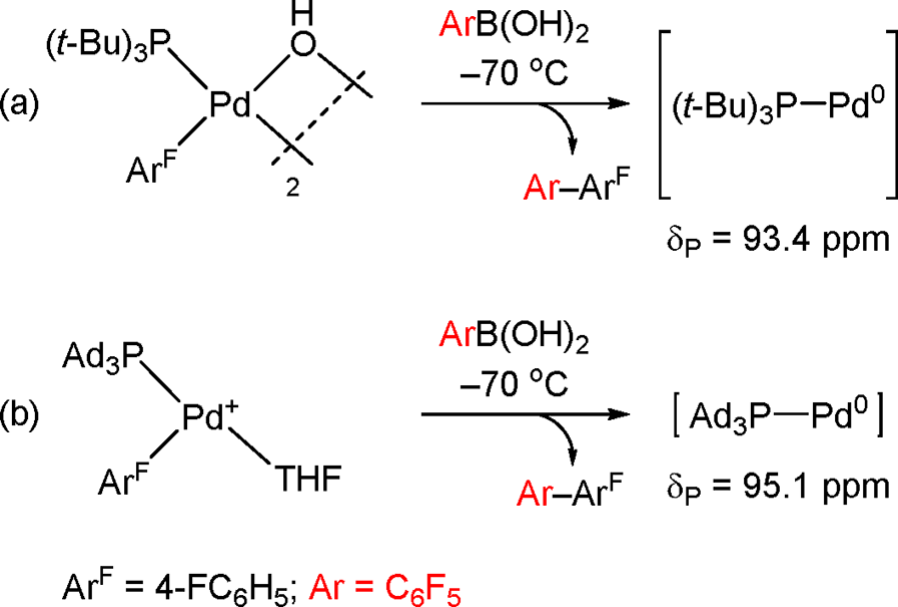
Synthesis and Low-Temperature ^31^P-NMR Identification of the Long-Sought-After “L_1_Pd(0)” with (a) L = P(*t*-Bu)_3_ and (b) L = P(Ad)_3_

Carrow’s recent work has been crucial in establishing the existence of L_1_Pd(0) (L = P(*t*-Bu)_3_; P(Ad)_3_) via the synthetic route outlined in Scheme 18.^137^

### 4.2 [L_1_Pd(I)X]_2_ and [L_1_Pd(II)X_2_]_2_ Dimers as Precatalysts

#### 4.2.1 [L_1_Pd(I)X]_2_ Dimers (X = Br, I) as Precatalysts

Palladium typically forms complexes with 0, +2, or +4 oxidation states. Palladium(I) is relatively rare even in catalysis. The presence of unpaired electrons in Pd(I) potentially favors the formation of a dimeric complex, considering the thermodynamic stability of the dimer (Pd–Pd bond energy is usually worth about 25 kcal/mol).^138^ Although over 50 Pd(I) dimers have been reported in the literature over the past decades, only very few are known to be catalytically active,^139,140^ and a few good reviews of their structural diversity have been published.^141−144^

**Scheme 19:**
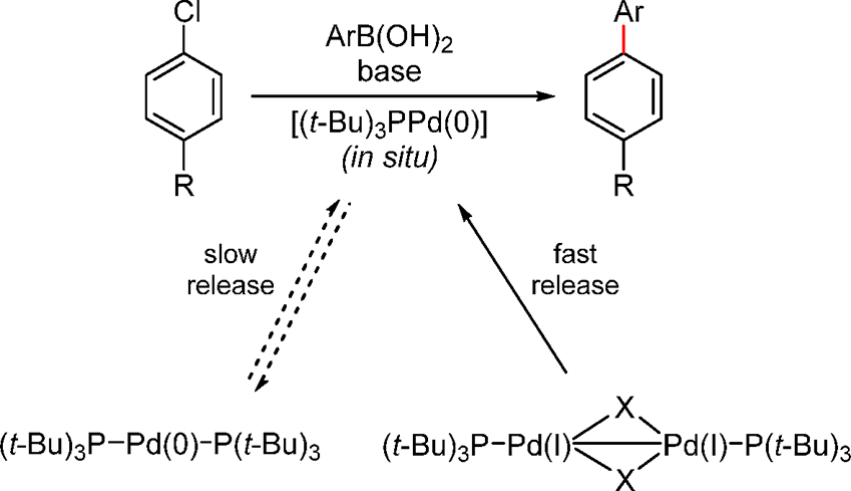
Fast Release of [(*t*-Bu)_3_PPd(0)] in Situ from the [(P(*t*-Bu)_3_)Pd(μ-Br)]_2_ Dimer vs Its Slow Release from Pd(P(*t*-Bu)_3_)_2_

**Figure 3:**
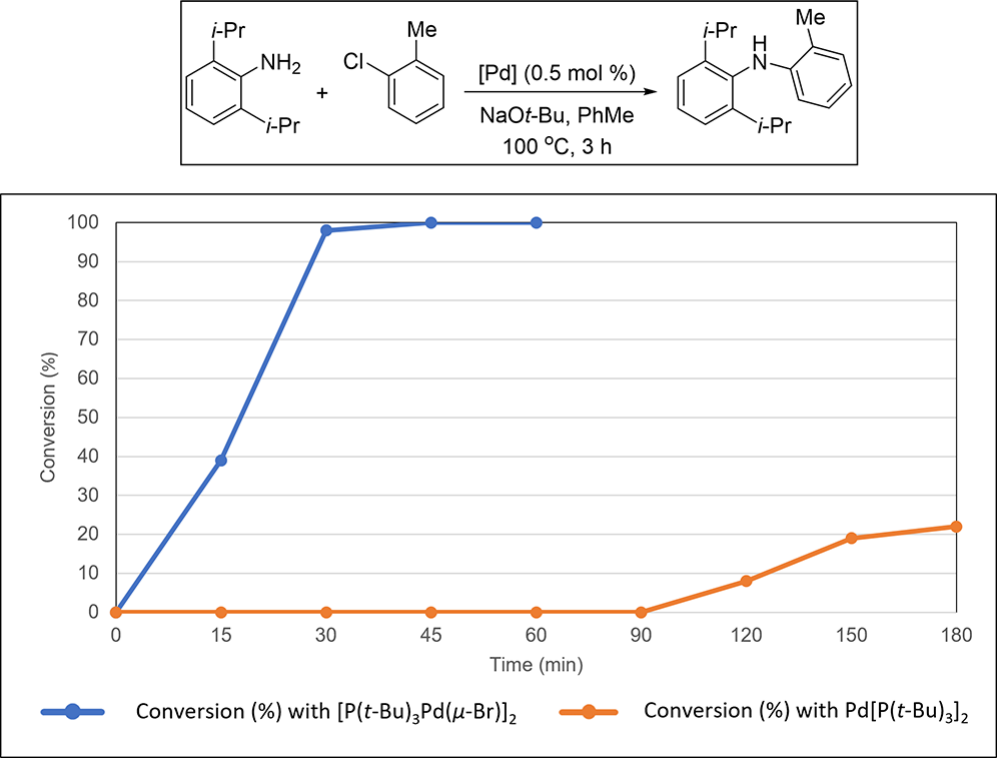
Fast reactivity of [P(*t*-Bu)_3_Pd(μ-Br)]_2_ dimer vs slow reactivity of Pd[P(*t*-Bu)_3_]_2_ in the C–N cross-coupling.

Based on our knowledge in this area, [(P(*t*-Bu)_3_)Pd(μ-Br)]_2_ is the first example of a well-studied monoligated Pd(I) precatalyst.^145^ Although, as early as 1996, Mingos and co-workers had synthesized [(P(*t*-Bu)_3_)Pd(μ-Br)]_2_ as an air-sensitive, dark-green 16-electron complex,^39,146,147^ its superior catalyst activity was disclosed by Hartwig and co-workers in 2002,^148,149^ followed by Prashad and coauthors in 2003.^150^ In 2012, Gooßen’s group identified it as a highly active isomerization catalyst for the synthesis of enol esters from allylic esters.^151^ [(P(*t*-Bu)_3_Pd(μ-Br))]_2_ has been employed as a precatalyst,^145,152,153^ which was assumed, and later confirmed,^154^ to function as an in situ reservoir of the highly reactive 12-electron complex [P(*t*-Bu)_3_)Pd(0)], that readily activates aryl halides (Scheme 19).^139^ This reactivity difference between Pd[P(*t*-Bu)_3_]_2_ and [(P(*t*-Bu)_3_Pd(μ-Br))]_2_ was clearly demonstrated in the C–N cross-coupling reactions as well (Figure 3).^32^

The major applications of this unique catalyst in coupling reactions such as C–N cross-couplings, cyanation, thiolation, α-arylation, and the Suzuki–Miyaura and other cross-couplings have been reviewed elsewhere.^2,135,139,145^

**Scheme 20:**
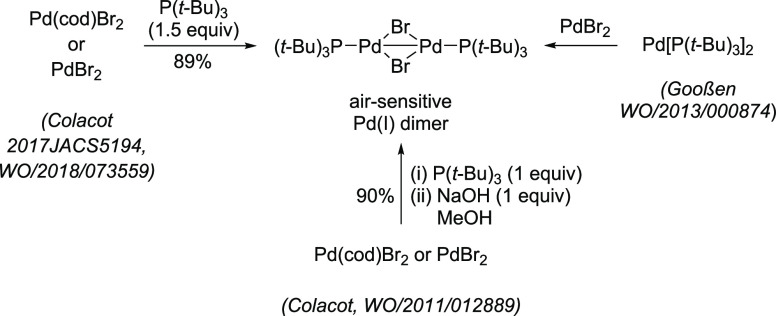
Practical Routes for the Synthesis of Bromo-Bridged Pd(I) Dimer, [P(*t*-Bu)_3_Pd(μ-Br)]_2_

Despite its unique and superior reactivity in challenging cross-coupling reactions, when compared to the in situ system or to Pd[P(*t*-Bu)_3_]_2_, making this air-sensitive precatalyst commercially available by using literature procedures^146,147,155^ had been a significant challenge until 2010–2011, when Colacot et al. discovered an atom-economical way to make it by reacting Pd(cod)Br_2_ or PdBr_2_ with 1 equiv of P(*t*-Bu)_3_, followed by addition of NaOH (1 equiv) in methanol.^156^ In 2017, while studying, with the aid of DFT calculations, the mechanism of this atom-economical and very interesting transformation in collaboration with Shoenebeck et al., it was found experimentally that the same transformation is possible with excellent selectivity by using 1.5 equiv of P(*t*-Bu)_3_ as well.^157^ Moreover, Goossen et al. published their findings in this regard in 2013.^158^ As of today, and although all three processes are patented, these remain the best methods for preparing [P(*t*-Bu)_3_Pd(μ-Br)]_2_ in very high yield (Scheme 20).

**Scheme 21:**
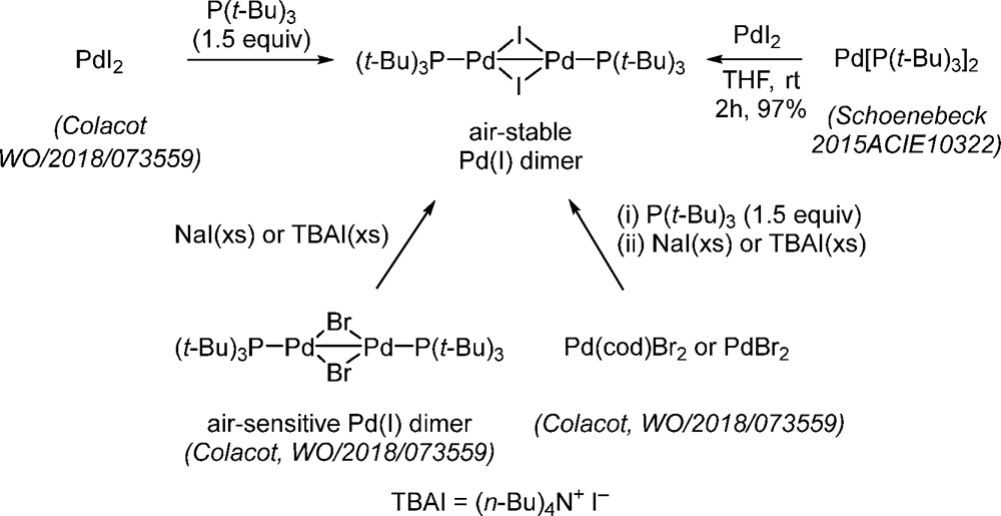
Practical Routes for the Synthesis of the Iodo-Bridged Pd(I) Dimer, [P(*t*-Bu)_3_Pd(μ-I)]_2_

The corresponding iodo dimer, [P(*t*-Bu)_3_Pd(μ-I)]_2_, has been investigated as a cross-coupling catalyst by Schoenebeck and co-workers.^139,159^ Schoenebeck’s group also developed a direct comproportionation method to synthesize the iodo-bridged dimer, [P(*t*-Bu)_3_Pd(μ-I)]_2_, while Colacot et al. developed three convenient routes either directly from PdI_2_ or from PdBr_2_/Pd(cod)_2_Br_2_ via the corresponding bromo dimer, [P(*t*-Bu)_3_Pd(μ-Br)]_2_ (Scheme 21).^157^

**Figure 4:**
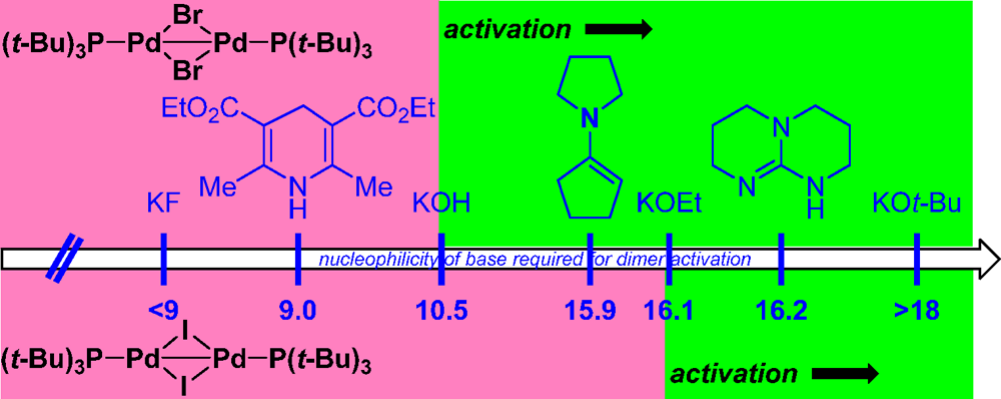
Schoenebeck’s chart for nucleophilic Pd(I) dimer activation to generate the active catalytic species, [Pd(0)P(*t*-Bu)_3_].

In contrast to the Pd(I) bromo dimer, the Pd(I) iodo dimer was not reactive under comparable conditions. This explains why it had only one known application relating to carbonylations of aryl halides,^160^ prior to the detailed investigations by Schoenebeck’s group, who showed that the in situ release of the 12-electron-based Pd(0) species [PdP(*t*-Bu)_3_] from these Pd(I) dimers is dependent on the adequate choice of additive.^161^ Schoenebeck’s study (Figure 4) using the *N* scale^162^ identified the minimum nucleophilicity required to effect the activation in each case. Using nucleophiles with *N* ≥ 16, Pd(I) iodide dimer was successfully activated for the Suzuki–Miyaura cross-coupling.^161^

**Scheme 22:**
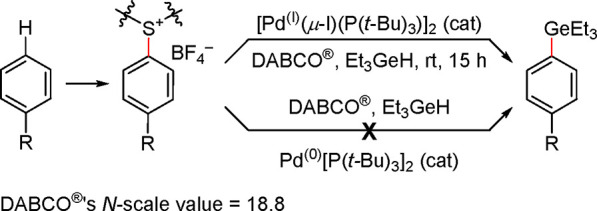
DABCO Activation of Dimer [Pd(μ-I)(P(*t*-Bu)_3_)]_2_ for a Mild and Selective Method of Direct Aryl C–H Activation Leading to Aryl Germanes

Schoenebeck’s group determined that DABCO (Evonik Operations GmbH), a nucleophile with an *N*-scale value of 18.80,^163^ is suitable for the activation of dimer [P(*t*-Bu)_3_Pd(μ-I)]_2_ in a mild and selective method for the direct aromatic C–H activation to form aryl germanes via the tetrafluorothianthrenium salt (Scheme 22).^139,164^ The corresponding L_2_Pd(0) based precatalyst, [Pd(P(*t*-Bu)_3_)]_2_, was ineffective in this transformation.

Based on computational and experimental studies, Schoenebeck’s group proposed a dinuclear mechanism during catalysis.^165,166^ Transition-state calculations suggested that bond activation occurs primarily at one Pd center of the Pd(I) dimer. After oxidative addition, a Pd(II) dimer was computationally obtained, suggesting that an overall Pd(I)–Pd(I)/Pd(II)–Pd(II) oxidative addition occurs involving both Pd centers vs a mechanism involving Pd(I)/Pd(III). Details of the application studies have been summarized in the mini-reviews by Schoenebeck^139^ and by Shaughnessy.^135^

#### 4.2.2 [L_1_Pd(II)X_2_]_2_ (X = Br and Cl) Dimers as Precatalysts

**Scheme 23:**
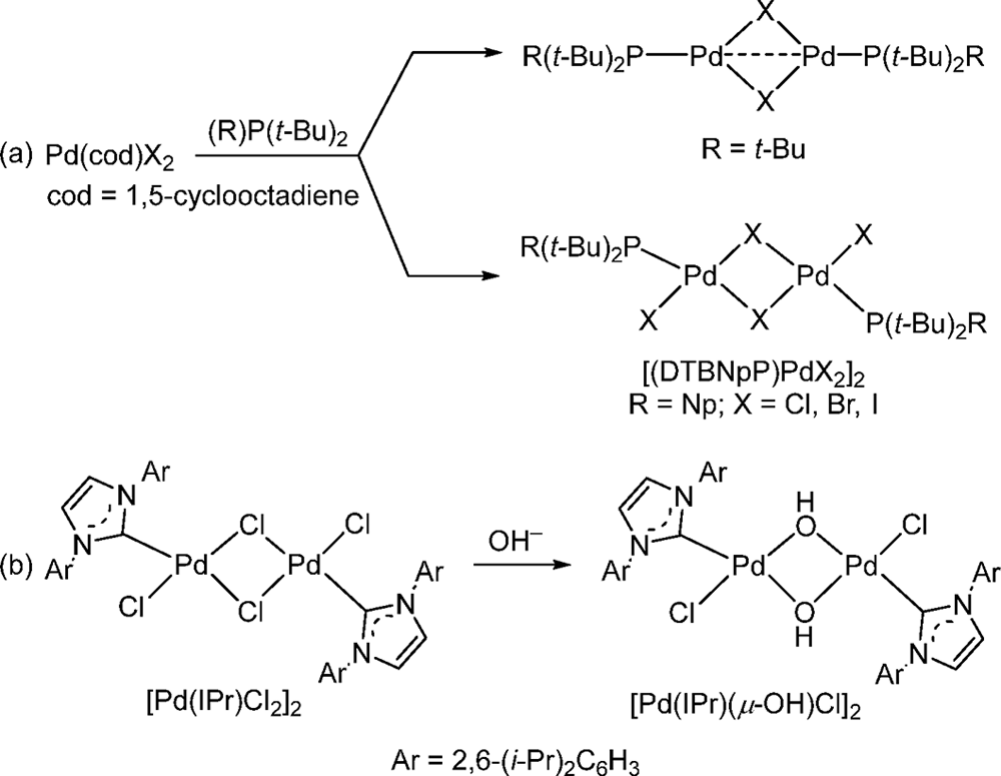
(a) Monoligated Pd(I) vs Pd(II) Complexes by Slightly Altering the Structure of the Phosphine Ligand R; (b) NHC-Based L_1_Pd(II) Complexes

Recently, Shaughnessy and co-workers carried out a detailed study using Np-based ligand systems to synthesize [L_1_PdX_2_]_2_ complexes, where changing the ligand from P(*t*-Bu)_3_ to neopentyl-based systems gave a monocoordinated Pd(II) in preference to a monocoordinated Pd(I) dimer.^135,167^ A year earlier, Watson’s group developed the Pd(II) dimer [(JessePhos)PdI_2_]_2_ (JessePhos = *tert*-butyldi(3,5-di-*tert*-butylphenyl)phosphine) for the coupling of silyl iodides and alkenes with yields comparable to those obtained with in situ catalysis using JessePhos in conjunction with Pd_2_(dba)_3_ but with significantly lower catalyst loadings.^168^ However, these types of complexes are not completely new; many examples using NHC had been synthesized and characterized by Nolan’s group as early as 2002.^169^ The NHC-based L_1_Pd(II) complexes (Scheme 23) were utilized effectively by Nolan and Cazin for various types of cross-coupling reactions such as aminations, Suzuki–Miyaura, Kumada–Corriu, and Heck–Mizoroki.^170−172^ The dimeric μ-hydroxide complex [(IPr)Pd(μ-OH)Cl]_2_ was formed under basic conditions during the Suzuki–Miyaura coupling. This complex promoted the coupling of aryl chlorides in the absence of an added base, whereas the parent [(IPr)PdCl_2_]_2_ gave no conversion under these conditions.^173^

### 4.3 Palladacycle Precatalysts

The development and application of palladacycles as precatalysts for cross-coupling reactions have been accomplished by several research groups; however, noteworthy original contributions came from the groups of Herrmann^174^ and Bedford.^175^ A few reviews have been published in this area.^176−178^ These precatalysts were popular in academia during that period, mainly because of their stability to air and moisture and high TONs when employed in relatively easy coupling reactions. Shaughnessy’s recent mini-review^135^ offers an overview of this area; hence, we do not intend to duplicate the effort; rather, we wish to highlight the precatalysts that are utilized in the R&D and process chemistry laboratories.

#### 4.3.1 Biphenylamine-Based Palladacycles

**Scheme 24:**
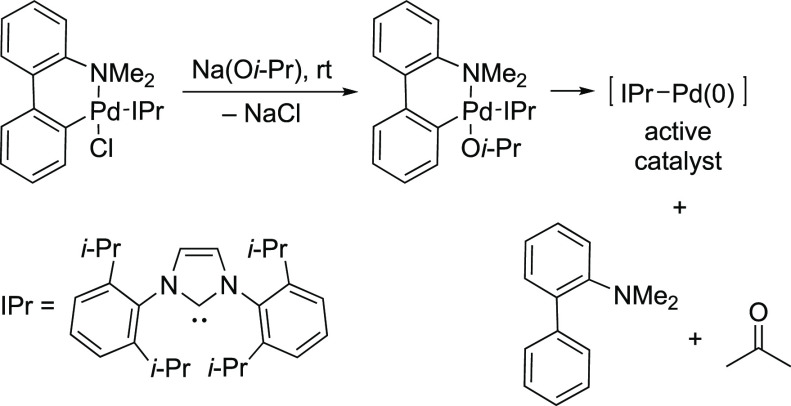
Nolan’s Proposed Mechanism for the Activation of NHC-Based Pd(II) Palladacycle to a 12-Electron-Based L_1_Pd(0)

Among the many examples of palladacycle precatalysts, amino-substituted-biphenyl-based palladacycles have recently become one of the most popular classes of precatalyst.^179^ Palladacycles derived from 2-(dimethylamino)biphenyl as a scaffold for use as precatalysts in cross-coupling applications such as the Buchwald–Hartwig, Suzuki–Miyaura, and enolate cross-couplings were originally developed by Nolan and co-workers by incorporating NHC-based ligands.^180−183^ Nolan also proposed a mechanism for the formation of the highly active monoligated L_1_Pd(0) from the stable Pd(II)-based palladacycle in the presence of a strong base such as sodium isopropoxide (Scheme 24).^182^

**Scheme 25:**
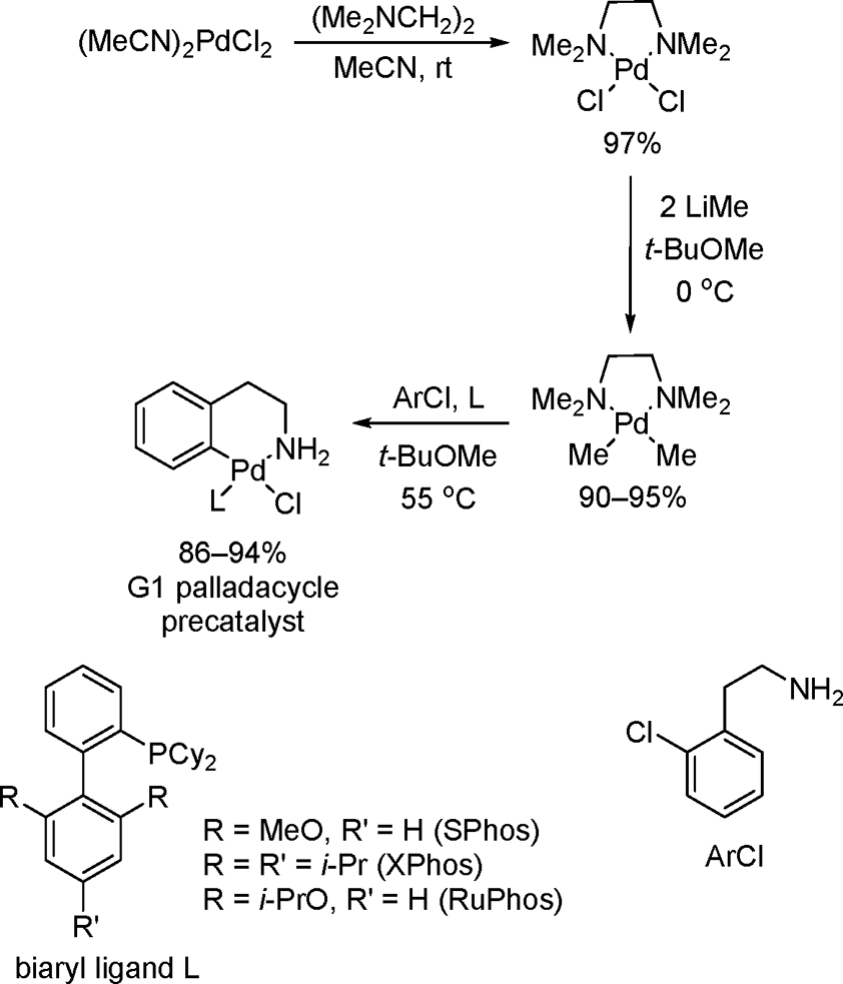
Buchwald’s Synthesis of the First-Generation (G1) Palladacycle Precatalysts

Buchwald and co-workers have developed several generations of palladacycles using similar or the same amine scaffold as in Nolan’s system. The first two generations of Buchwald palladacycle are based on Pd–Cl systems with κ^2^-*N*,*C* phenethylamine (G1)^184^ and 2-aminobiphenyl as the chelating *N*,*C*-ligand (G2),^185^ respectively. Although the G1 and G2 classes of precatalyst are superior to the in situ generated systems that use precursors such as Pd_2_(dba)_3_ or Pd(R-allyl)Cl, they nevertheless have many limitations. The multistep synthesis and scale-up of G1 is tedious, mainly because of the synthesis of the thermally unstable (TMEDA)PdMe_2_ and its conversion into G1 (Scheme 25).^184^ Moreover, the G1 precatalysts are slow to initiate reaction at room temperature in the presence of weak bases such as carbonate or phosphate.

**Scheme 26:**
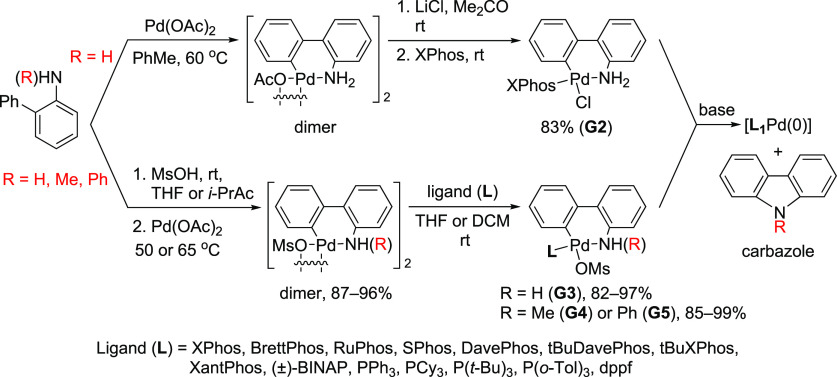
Buchwald’s G2–G5 Palladacycles and Their Activation to “[L_1_Pd(0)]” by Base

While one important drawback of the G1 precatalysts is their limited ability to accommodate bulkier ligands, they are nevertheless superior in certain coupling reactions to the G2^185^ and G3^186^ counterparts because they do not produce carbazole as inhibitor as Colacot and co-workers found in their comparison studies.^187^ In addition, 4-aminobiphenyl, present as an impurity in 2-aminobiphenyl, is very toxic; however, one can overcome this by using high-purity 2-aminobiphenyl as the starting material. Consequently, Buchwald’s lab developed the G4 and G5 versions of the palladacycle precatalysts.^188^ The general synthesis and activation of G2–G5 complexes are summarized in Scheme 26.

**Scheme 27:**
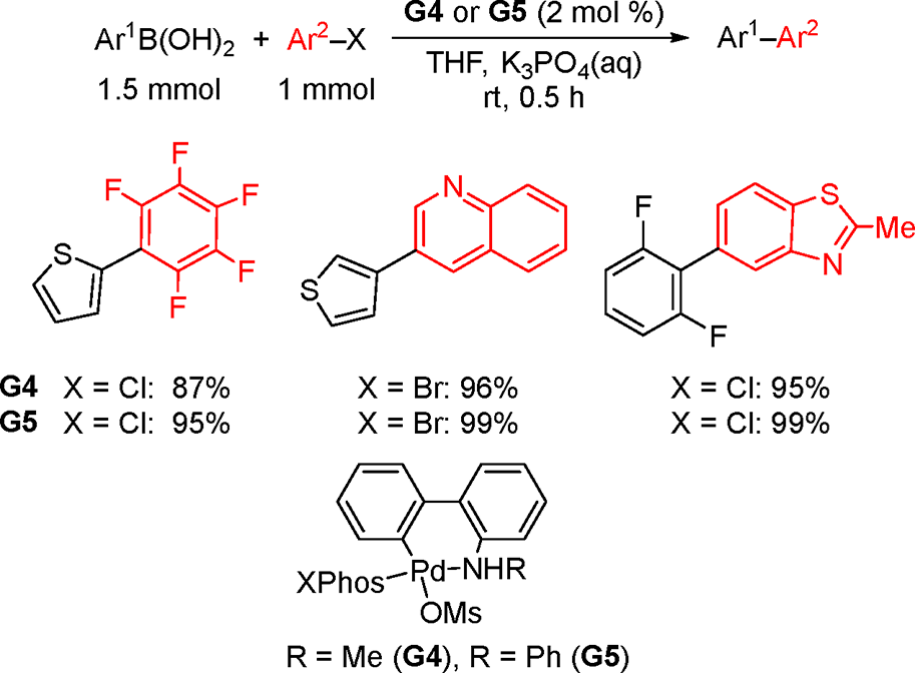
Applications of XPhos-Based Buchwald’s G4 and G5 Precatalysts in Challenging Suzuki–Miyaura Cross-Coupling Reactions

Although the Suzuki–Miyaura cross-coupling is well established, the coupling of relatively unstable fluoroboronic acids and heterocyclic boronic acids is challenging because they tend to undergo protodeborylation as they cannot withstand high temperatures and longer reaction times. Therefore, the active catalyst in these reactions, [L_1_Pd(0)], has to be generated in a facile manner and needs to effect the coupling rapidly under milder conditions. The advantages of XPhos-based G4 and G5 precatalysts for a series of challenging cross-couplings of boronic acids with aryl bromides and chlorides are showcased in Scheme 27.^188^ It is worth noting that the XPhos-based G2 precatalyst had earlier been effectively utilized by Buchwald for the Suzuki–Miyaura cross-coupling of unstable polyfluorophenyl and five-membered ring 2-heteroaryl boronic acids with aryl bromides, chlorides, and triflates.^185^

There are overlaps in reactivity between generations of G2–G5 precatalysts. G3s, however, appear to be a broader class of precatalysts because of the ability of the framework to accommodate bulky ligands such as tBuXPhos and tBuBrettPhos. In addition, G3s appear to have wider applications in coupling reactions such as the selective monoarylation of CH_3_-CO-R compounds, the coupling of challenging boronic acids under milder conditions, and the C–N coupling of primary and secondary amines.^186^ These G2–G5 complexes are relatively air-stable and commercially available in milligram-to-bulk quantities, although purity and reliability can vary from supplier to supplier.

#### 4.3.2 Acetanilide-Based Palladacycles

**Figure 5:**
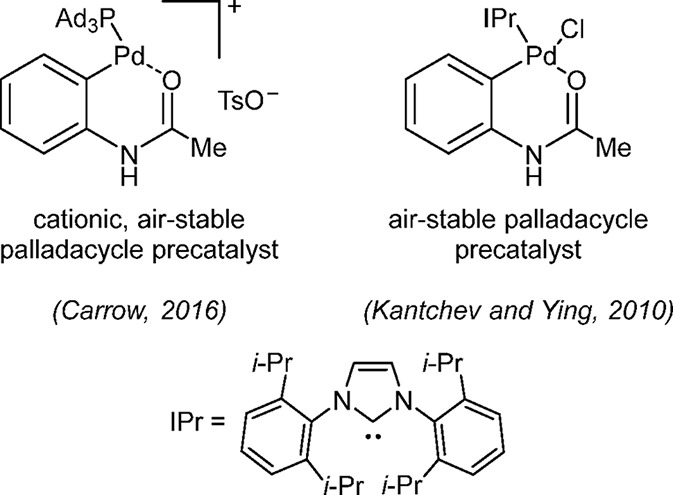
Acetanilide-based palladacycles with ligands such as tertiary phosphines (e.g., PAd_3_) and NHC (e.g., IPr).

Carrow and co-workers have reported the synthesis and applications of a tri(adamantyl)phosphine-based acetanilide palladacycle (Figure 5) and demonstrated its superior activity in the Suzuki–Miyaura coupling of aryl bromides and chlorides with the challenging polyfluorophenyl boronic acids including, C_6_F_5_B(OH)_2_.^189^ Although the same group did provide a synthetic route for the known ligand PAd_3_, its synthesis in bulk quantity is not trivial. Prior to this report, in 2010, Kantchev and Wing prepared an IPr complex and compared the activity of several IPr-coordinated palladacycle precatalysts in the Suzuki–Miyaura and C–N/P/S/O cross-couplings; they found that this catalyst system is superior to the corresponding PEPPSI, cinnamyl, and acac systems.^190^

### 4.4 L_1_Pd-Based Acyclic Precatalysts

This section describes a class of monomeric precatalysts that represents analogous technological advancements in the synthesis of monoligated Pd complexes and their applications to cross-coupling reactions, with an emphasis on activity, selectivity, and efficiency. However, the precursor to these Pd complexes can be a monomer or dimer. This section also explains how different types of precatalysts could be activated to L_1_Pd(0) while minimizing the “off-cycle species.” We hope that using the information provided below, the users from both academia and industry can make a decision in selecting a specific cross-coupling reaction by generating the most appropriate active catalytic species.

#### 4.4.1 Phosphine-Based π-(Allyl, Crotyl, and Cinnamyl) Precatalysts

**Scheme 28:**
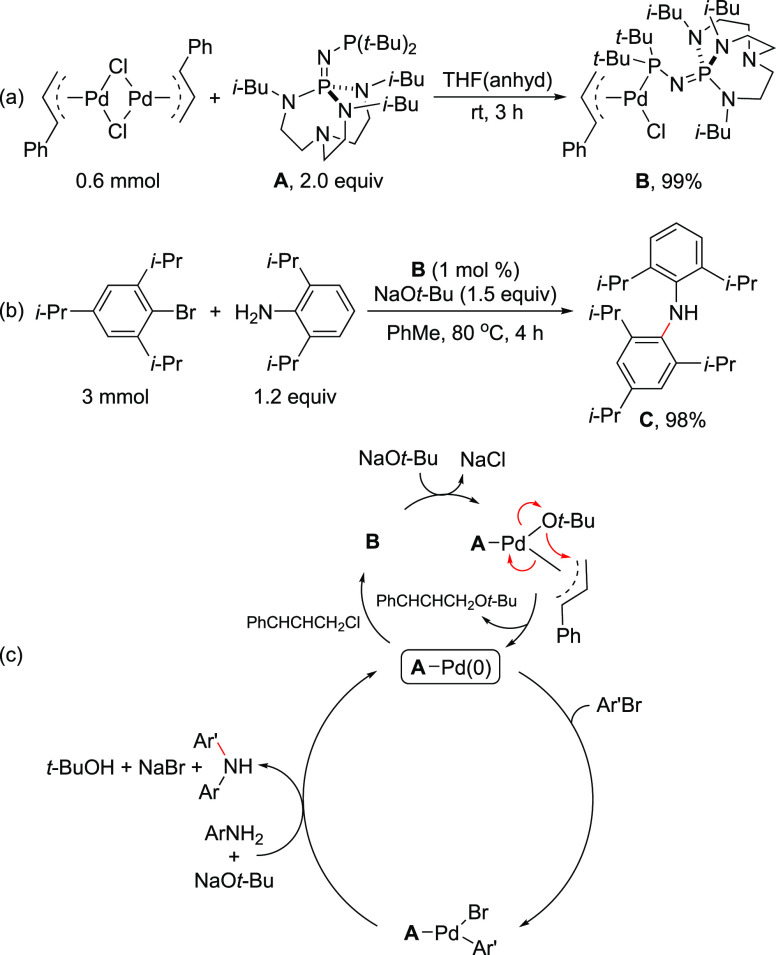
(a) Verkade’s Synthesis of η^3^-Cinnamyl-Based Monoligated Pd Complexes, (b) Their Application in Challenging Buchwald–Hartwig Aminations of Sterically Hindered Amines and Aryl Halides, and (c) Proposed Mechanism for the Amination

A notable report on the synthesis and applications of the tertiary-phosphine-based Pd(π-allyl)(L)Cl, where L = di(*tert*-butyl)(neopentyl)phosphine (DTBNpP), was published jointly in 2010 by Shaughnessy’s and Colacot’s groups.^191^ Prior to this report, an important publication from Verkade’s group described the synthesis and full characterization of moisture- and air-stable monoligated (η^3^-R-allyl)Pd(L)Cl complexes, where R is Me or Ph and L is the bulky phosphinimine ligand (*t*-Bu)_2_PN = P[N(*i*-Bu)CH_2_CH_2_–]_3_N (Scheme 28).^192^ These complexes were successfully employed by Verkade for the amination of aryl bromides and chlorides, including challenging Buchwald–Hartwig aminations of sterically hindered amine and halide coupling partners.

To clarify the influence of phosphine ligand L and the R-allyl moiety on the reactivity of these complexes, Verkade’s team conducted a series of control experiments. These investigations revealed that the ancillary cinnamyl moiety leads to reduction of the ligand loading by half in comparison to the in situ generated catalyst whereby 2 equiv of L are required to achieve high yields, regardless of whether the Pd source is the acetate or the chloride salt. This may be attributed to the need for an extra equivalent of the ligand to reduce Pd(II) to Pd(0) during the in situ catalysis involving both Pd(OAc)_2_ and PdCl_2_, whereas preformed complexes of type **B** (see Scheme 28) are activated to L_1_Pd(0) in the presence of a base. The analogue of complex **B** in which the ancillary cinnamyl ligand is replaced with crotyl (possessing the smaller Me group) resulted in a slightly lower isolated yield of the Buchwald–Hartwig coupling product **C** (87%) compared with 98% for the cinnamyl analogue depicted in Scheme 28(192) In spite of the unique and superior reactivity of **B** and its crotyl analogue, they are, to our knowledge, not commercially available, presumably owing to the time-consuming and tedious synthesis of the requisite ligand **A**.

**Scheme 29:**
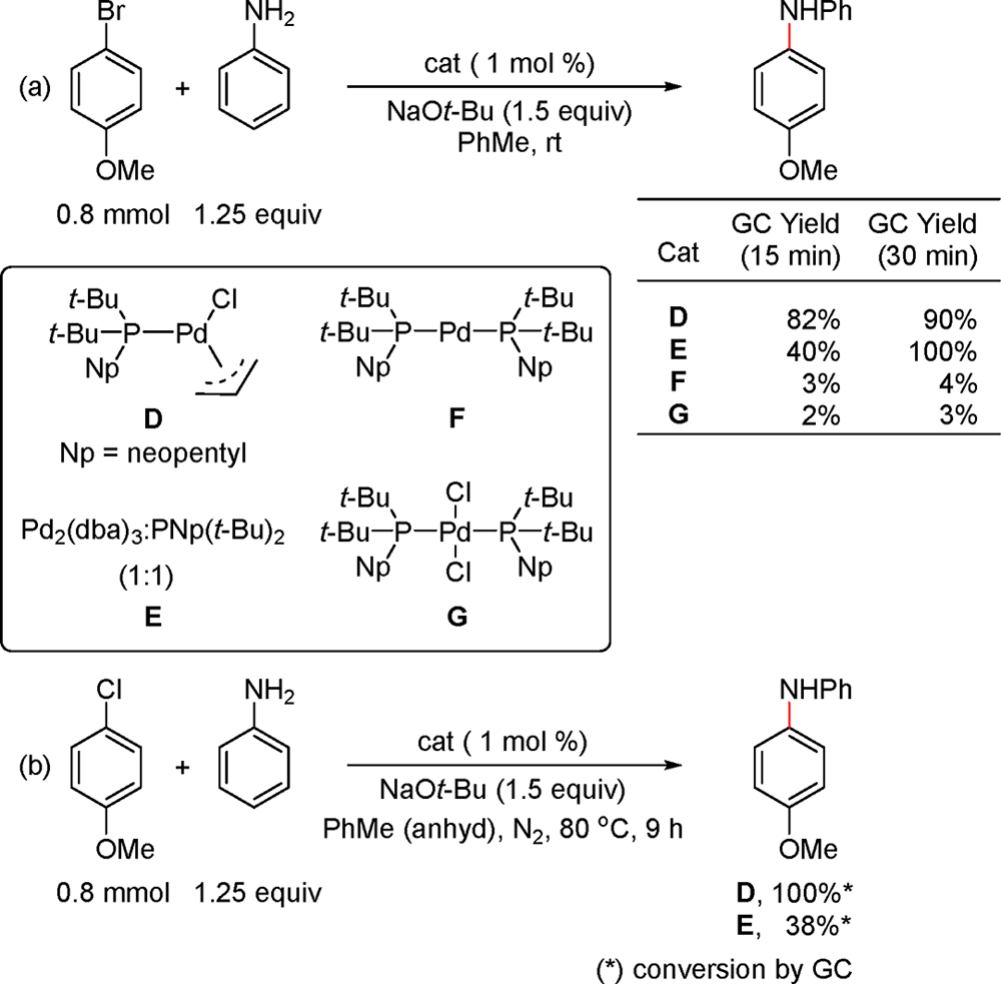
(a) Performance of Precatalysts **D**, **F**, and **G**, and in Situ Generated Catalyst from E in the Buchwald–Hartwig Coupling of Aniline with 4-Bromoanisole; (b) Control Experiments in Which the Ligand:Pd Ratio Is Kept at 1:1

**Figure 6:**
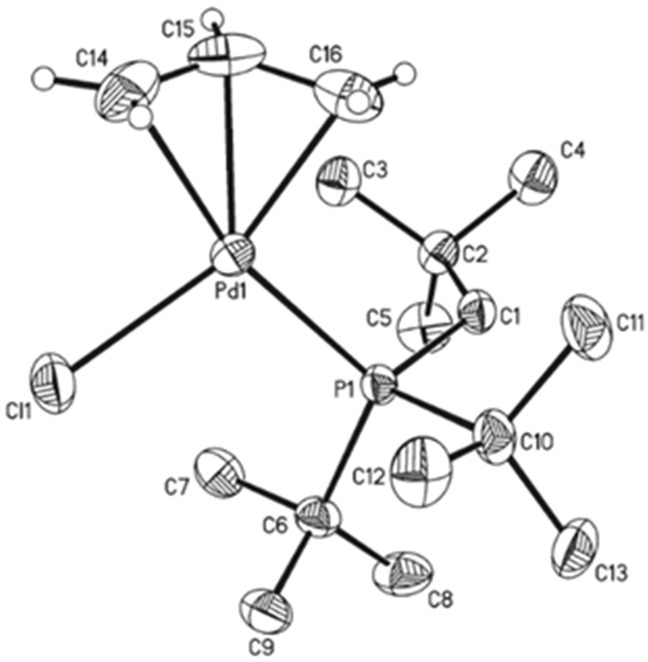
Thermal ellipsoid plot (50% level) of the molecular structure of Pd(π-allyl)(DTBNpP)Cl (**D**). Hydrogen atoms on the DTBNpP ligand have been omitted for clarity. Reproduced with permission from ref (191). Copyright 2010 American Chemical Society.

In the aforementioned report,^191^ Shaughnessy and Colacot established a clear reactivity difference between the L_2_Pd based precatalyst and the monocoordinated L_1_Pd complex of DTBNpP [(*t*-Bu)_2_NpP], Pd(π-allyl)[P(*t*-Bu)_2_(Np)]Cl in the Buchwald–Hartwig coupling. The L_1_Pd complex (**D**) exhibited superior reactivity vis-à-vis the L_2_Pd based precatalysts **F** and **G** as well as the catalytic system generated in situ from Pd_2_(dba_3_)–DTBNpP (1:1) (Scheme 29 and Figure 6).^191^

##### 4.4.1.1 Mechanism of Pd(R-allyl)(L)X Precatalyst Activation

**Scheme 30:**
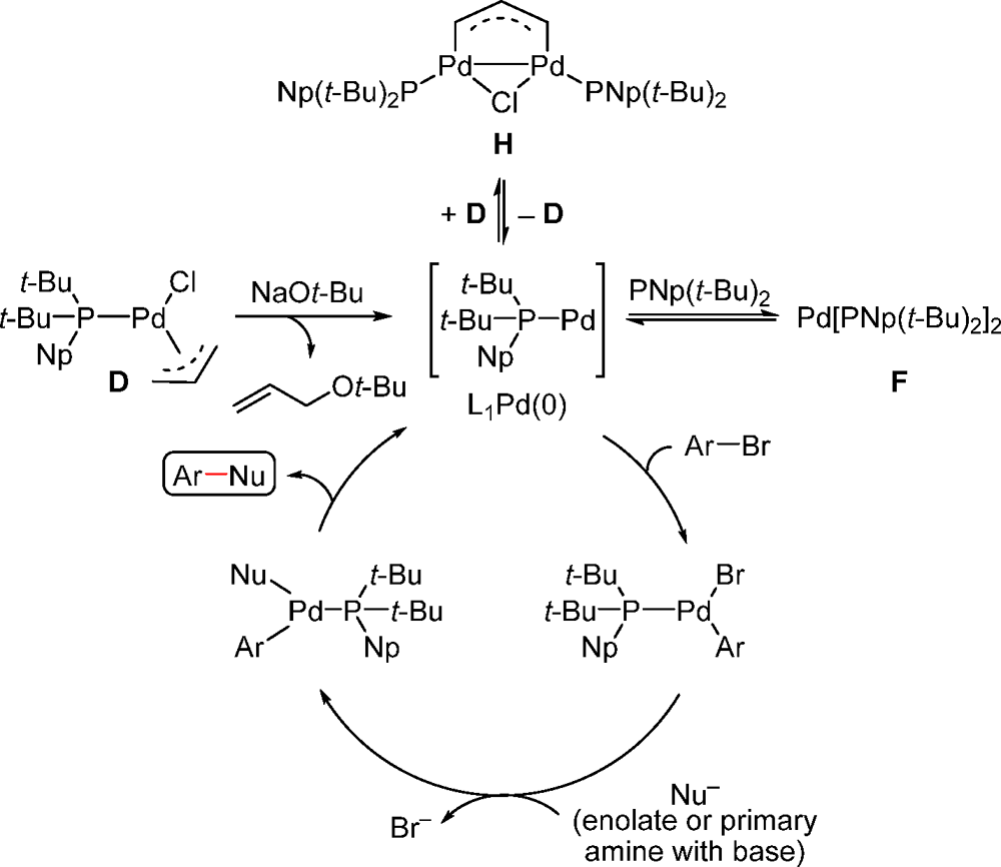
Proposed Mechanism of Activation of Pd(π-allyl)(*t*-Bu)_2_(Np)Cl (D) to the Catalytically Active L_1_Pd(0) Species and Subsequent Catalysis by the Latter of the Arylation of Ketones and Amines

Some of the most important findings from the work of Shaughnessy and Colacot are the activation mechanism of precatalyst **D**, the formation of the active 12-electron-based L_1_Pd(0) species, and the catalytic cycle of the arylation of ketones and amines (Scheme 30).^191^

Although L_1_Pd(0) has not been isolated or fully characterized, with the exception of Carrow’s very recent work,^137^ its presence has been well-documented by many (e.g., isolation and characterization of L_1_Pd(Ar)(X) by Buchwald^185^). Precatalyst **D**, in the presence of a base such as NaO*t*-Bu gets activated to L_1_Pd(0), which can further react with the starting precatalyst **D** to form a “comproportionation dimer” (**H**) (see Scheme 30). The thermodynamic stability of the dimer, **H**, determines the overall activity and efficiency of precatalyst **D** because the concentration of L_1_Pd(0) dictates the overall activity in the catalytic cycle. Moreover, the presence of excess ligand can lead to L_2_Pd(0) (**F**). NMR investigations indicated that formation of L_2_Pd(0) (**F**) is still observed during the in situ catalysis even with a 1:1 molar ratio of Pd:ligand. Although L_2_Pd(0) (**F**) can dissociate to form L_1_Pd(0) in theory, it is a slower process than the generation of L_1_Pd(0) directly from a suitable precatalyst such as **D**.

##### 4.4.1.2 Role of the Conventional Monodentate Ligands and the R Group in R-allyl in Precatalyst Activation

**Figure 7:**
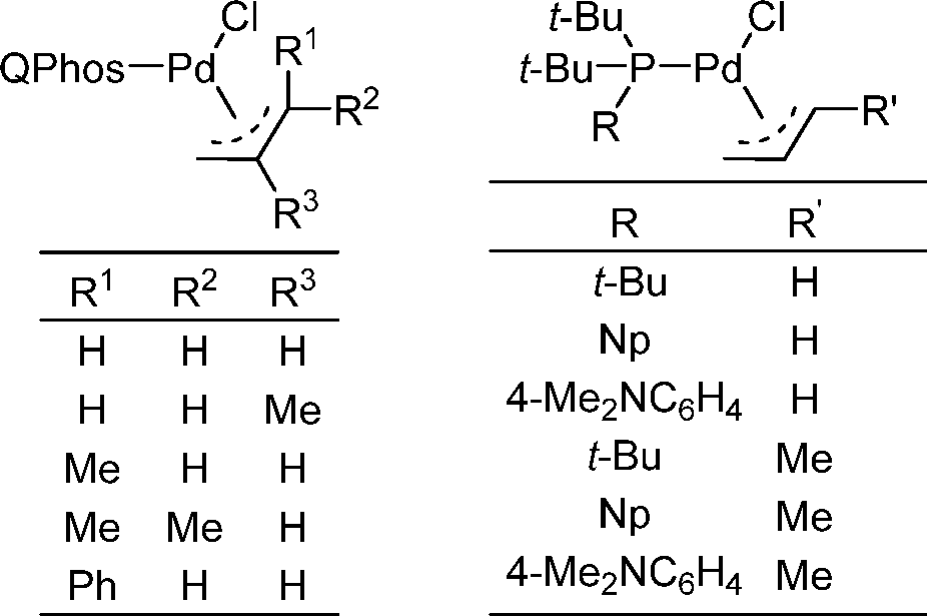
Varying the allyl group and the ligand L in Pd(π-R-allyl)(L)X complexes to study their effects on the reactivity of the complexes.

Colacot and co-workers further expanded the scope of their work with a wide range of Pd(R-allyl)LCl complexes by varying the ancillary R-allyl moiety (R = H, 1-Me, 2-Me, 1-*gem*-Me_2_, 1-Ph) and the electronics and sterics of the ligand [L = QPhos, P(*t*-Bu)_3_, P(*t*-Bu)_2_Np, (*t*-Bu)_2_(4-Me_2_NC_6_H_4_)P] to understand the role of the ligand and the substituents on the allyl group (Figure 7).^193^

**Figure 8:**
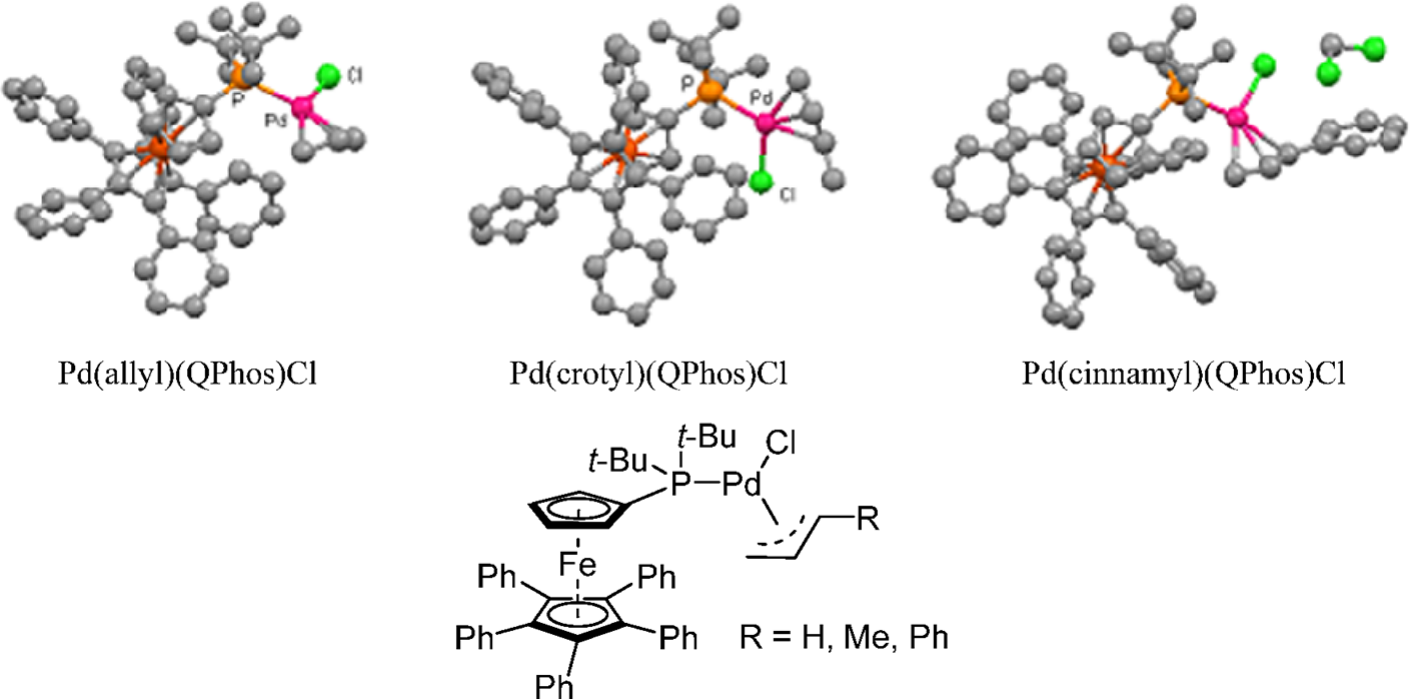
X-ray crystal structures of Pd(allyl)(QPhos)Cl, Pd(crotyl)(QPhos)Cl, and Pd(cinnamyl)(QPhos)Cl. Reproduced with permission from ref (193). Copyright 2011 American Chemical Society.

**Scheme 31:**
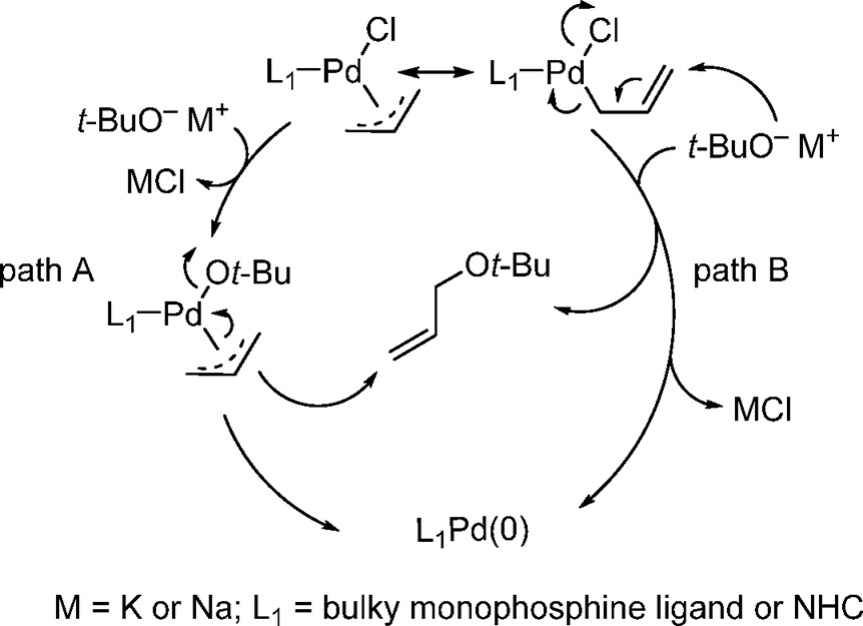
Suggested Activation Pathways for Pd(π-R-allyl)(L)Cl Complexes in Analogy to Nolan’s Proposed Activation Mechanism for Related NHC-Based Complexes

**Scheme 32:**
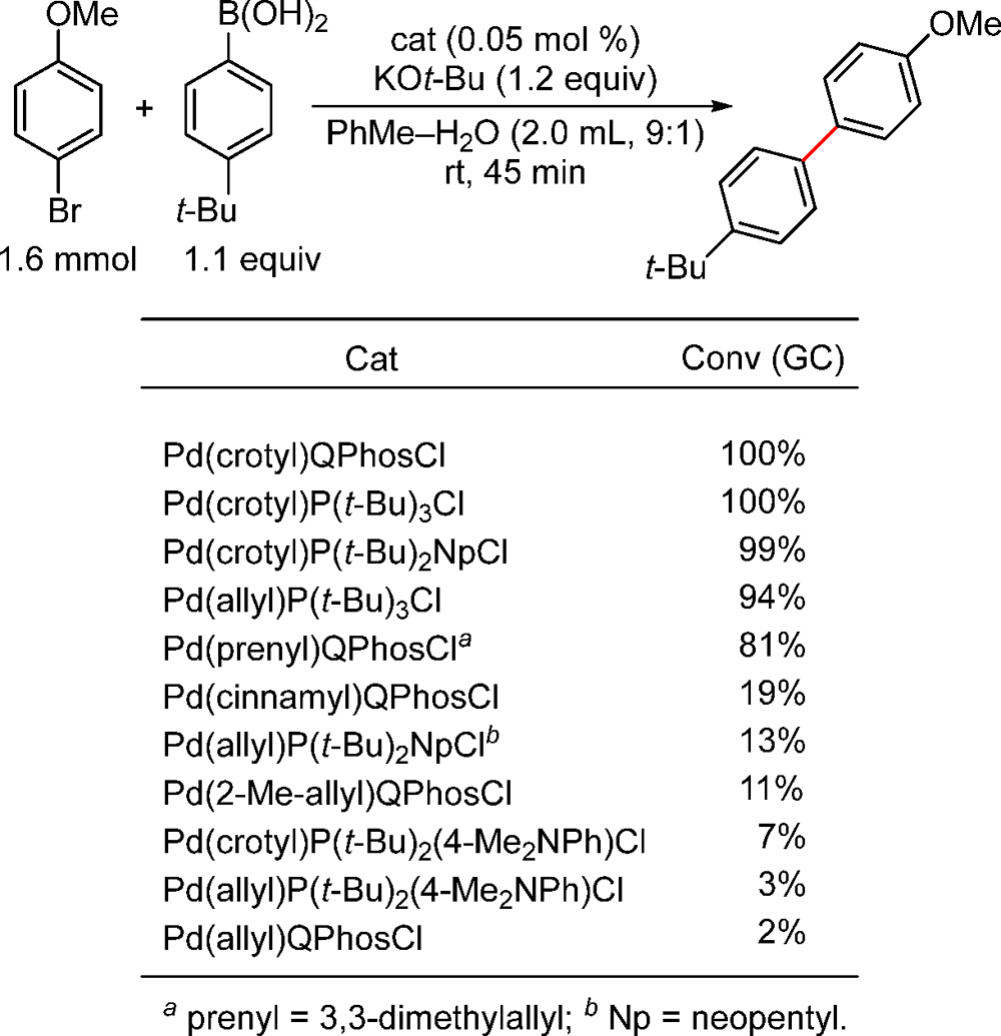
Evaluation of the Activity of (π-*R*-Allyl)Pd(L)Cl Catalysts in a Model Suzuki–Miyaura Coupling Reaction at rt

The X-ray crystal structures of the QPhos-based (π-R-allyl)Pd complexes (Figure 8)^193^ revealed an increase in dissymmetry of the π-R-allyl moiety in the order Pd(allyl)(QPhos)Cl < Pd(crotyl)(QPhos)Cl < Pd(cinnamyl)(QPhos)Cl, not unlike what Nolan observed for his NHC-based systems.^194^ The activation pathway involves either an alkoxide for chloride exchange (path A) or a nucleophilic attack by the *tert*-butoxide anion onto the π-allyl moiety (path B), leading to the L_1_Pd(0) active species (Scheme 31).^193,194^ In agreement with Nolan’s observation in his studies on the NHC systems, the dissymmetry could in theory affect the rate of the activation step and hence the rate of the coupling reaction. However, one should not extrapolate from these studies that the superior activity of the cinnamyl-based catalyst will apply to all cross-coupling reactions, because, in various cases, crotyl complexes give similar or better reactivities as exemplified by results from a model Suzuki–Miyaura cross-coupling reaction at room temperature (Scheme 32).^193^

It is clear from this study that the crotyl-based catalysts appear to be superior to the other R-allyl catalysts. Surprisingly, the cinnamyl-based precatalyst showed lower activity in this Suzuki–Miyaura coupling, in contrast to Nolan’s observation with the NHC systems.^194^ In general, the size of L and the substituents on the allyl group are important factors that influence the activity of this class of precatalysts: Pd(crotyl)(L)Cl, where L = QPhos, (*t*-Bu)_3_P, or (*t*-Bu)_2_NpP, stand out as performing the best.

**Scheme 33:**
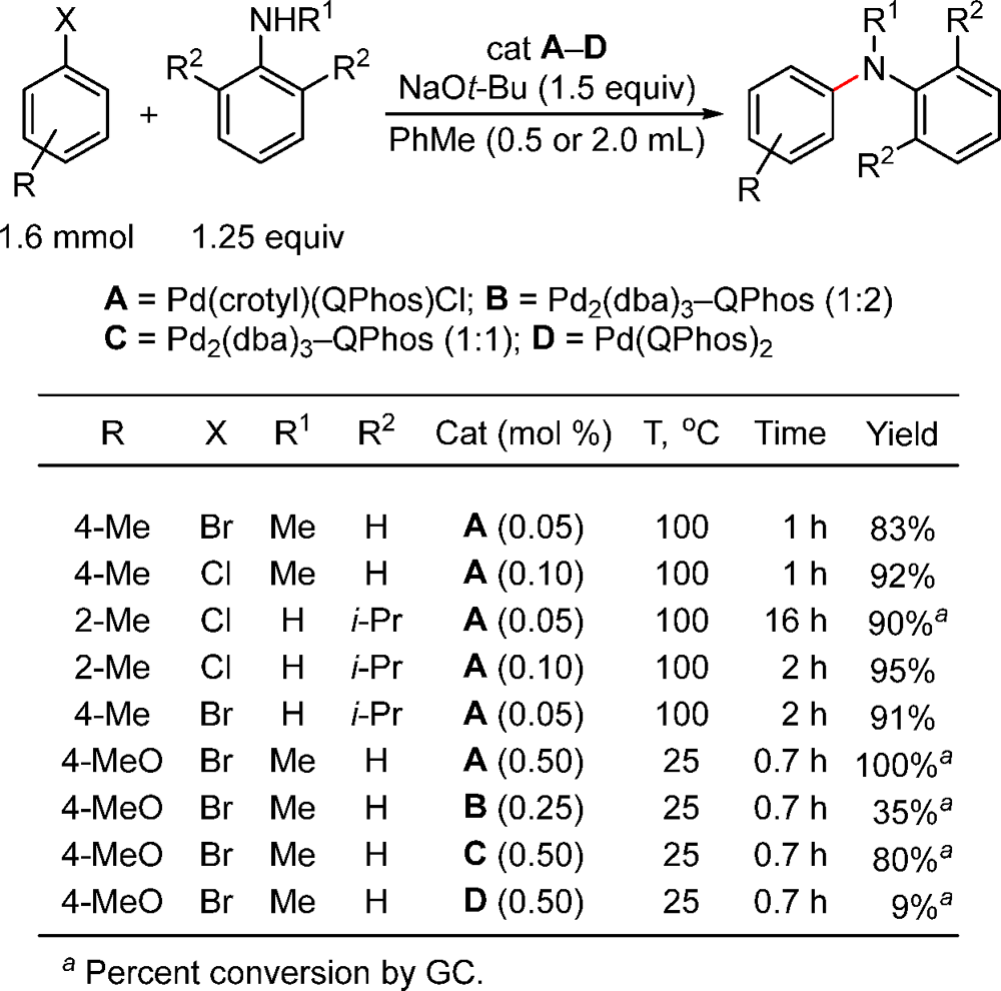
Superior Reactivity of Preformed Pd(crotyl)(QPhos)Cl in the Buchwald–Hartwig Amination at Low Loadings

In a related investigation of several catalysts in the Buchwald–Hartwig amination, Pd(crotyl)(QPhos)Cl performed the best, with very low loading (Scheme 33).^193^ In contrast, the α-arylation of 1-tetralone under analogous conditions (dioxane, 100 °C) proceeded best with Pd(allyl)-based catalysts, whereas use of the Pd(crotyl)-based ones resulted in poor yields.^193^

##### 4.4.1.3 Role of the L_2_Pd_2_(allyl)X “Comproportionation” Dimer in Catalysis

**Scheme 34:**
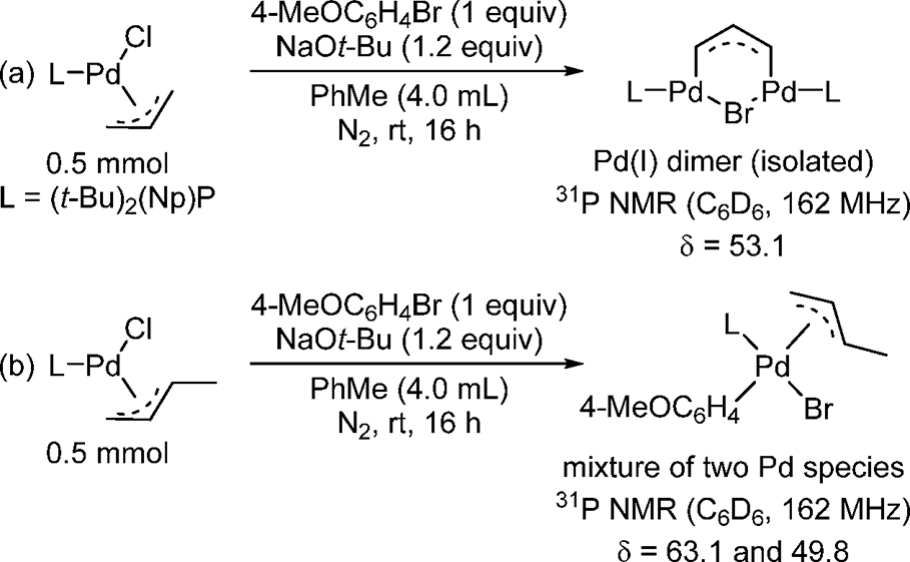
Formation of the Pd(I) Comproportionation Dimer from Pd(π-*allyl*)(L)Cl but Not Pd(*crotyl*)(L)Cl in the Oxidative Addition Step of the Buchwald–Hartwig Amination of *N*-Methylaniline with 4-Bromoanisole

One of the very interesting findings of Colacot’s work is that all of the Pd(π-allyl)(L)Cl precatalysts investigated [L = (*t*-Bu)_3_P, QPhos, (*t*-Bu)_2_(Np)P, (*t*-Bu)_2_(4-Me_2_NC_6_H_4_)P] produce the corresponding Pd(I)(allyl)-based “comproportionation” dimers upon treatment with *t*-BuONa as well as during the oxidative addition step, whereas no such major comproportionation dimer is observed with Pd(crotyl)(L)X (X = Cl, Br) under identical conditions (Scheme 34).^193^ The comproportionation dimer was proven, by isolation and X-ray crystal structure analysis, to be the main intermediate in the Buchwald–Hartwig amination of *N*-methylaniline with 4-bromoanisole.

#### 4.4.2 Pd(π-R-Allyl)(NHC)Cl Complexes

**Scheme 35:**
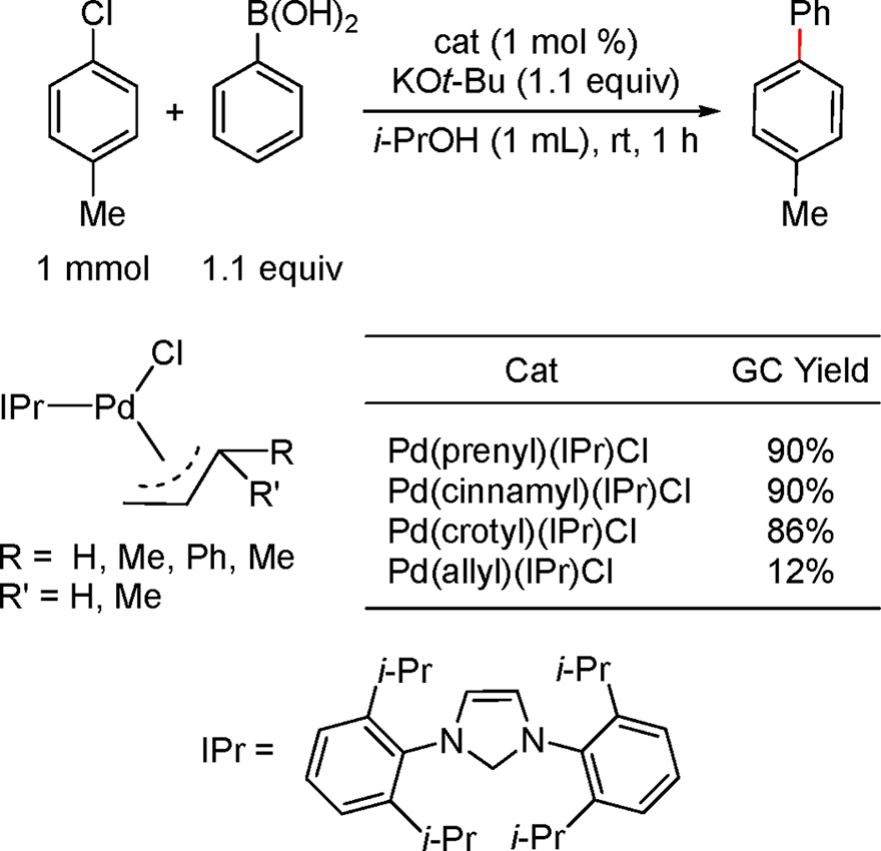
Nolan’s Model Suzuki–Miyaura Coupling Demonstrating the Catalyst-Activity-Enhancing Role of Bulky Substituents on the Allyl Group

Nolan’s group can be credited with developing and studying the applications of Pd(π-R-allyl)(NHC)Cl complexes as precatalysts that generate the monoligated species, (NHC)Pd(0), in the context of cross-coupling reactions. In their landmark publication in 2006,^194^ they screened the Pd(π-R-allyl)(NHC)Cl precatalysts in a model Suzuki–Miyaura coupling. This study demonstrated that precatalysts with an unsubstituted allyl are less active than those with a crotyl, cinnamyl, and prenyl analogues (Scheme 35).^194^ Although these authors did not provide a rationale as to why allyl resulted in lower conversions, subsequent studies by Hazari^195,196^ and later by Colacot^187^ identified Pd(I) allyl dimer formation as a detrimental “off-cycle” species that leads to lower activity (see section 4.4.1.3 above). Additionally, Nolan applied these precatalysts in Suzuki–Miyaura cross-couplings of aryl chlorides and bromides at rt and at 80 °C and in the Buchwald–Hartwig amination, including that of sterically hindered coupling partners.^194^

**Scheme 36:**
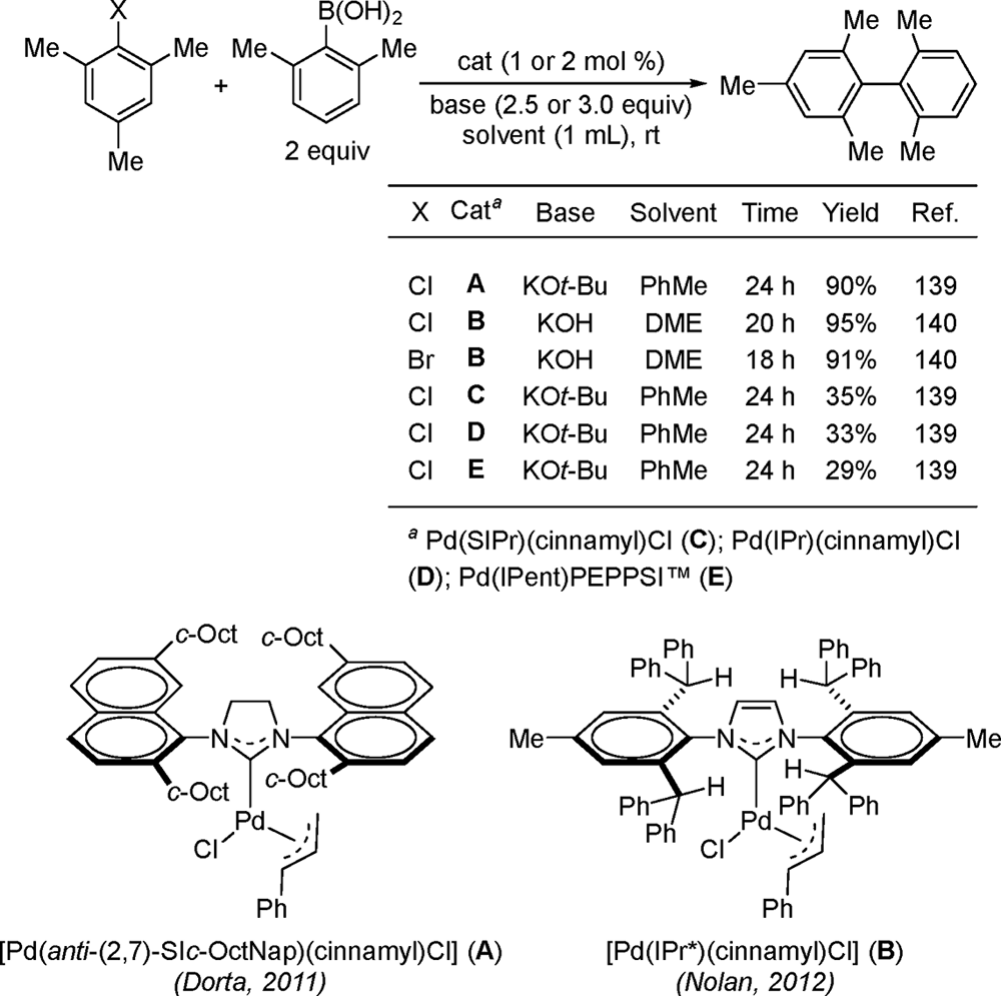
Performance of Dorta’s [Pd(*anti*-(2,7)-SI*c*-OctNap)(cinnamyl)Cl] (A) and Nolan’s [Pd(IPr*)(cinnamyl)Cl] (B) in Challenging Suzuki–Miyaura Cross-Couplings

In addition to the size of the R substituent on the allyl group, the size of the ligand also plays an important role in activating/deactivating the catalyst. For example, tetra-*ortho*-substituted biaryl synthesis was accomplished by using [Pd(*anti*-(2,7)-SI*c*-OctNap)(cinnamyl)Cl] precatalyst by Dorta and co-workers (Scheme 36).^197^ This catalyst was far superior (90% yield) than the corresponding IPr or SIPr precatalyst (33–35% yields) and Pd(IPent)PEPPSI)Cl (29% yield) for coupling of chloromesitylene with dimethylphenylboronic acid at rt with 2 mol % Pd loading. The superior activity of this catalyst was attributed to the percent buried volume (% *V*_Bur_) of 42.0 vs 36.7 and 37.0 for IPr and SIPr, respectively.^198^ Nolan applied this concept by utilizing the IPr* ligand (see Scheme 36 below), with a %*V*_Bur_ of 44.6%, to couple a variety of challenging ortho-disubstituted aryl and heteroaryl halides with ortho-disubstituted phenylboronic acid at rt using 1 mol % Pd loading. Nolan and Chartoire have published a detailed account of the use of various NHC-based precatalysts, including the synthesis and novel applications of Pd(R-allyl)(NHC)Cl complexes;^16^ hence, this chemistry will not be covered in this review and will be touched upon where warranted.

#### 4.4.3 Indenyl NHC Complexes

**Scheme 37:**
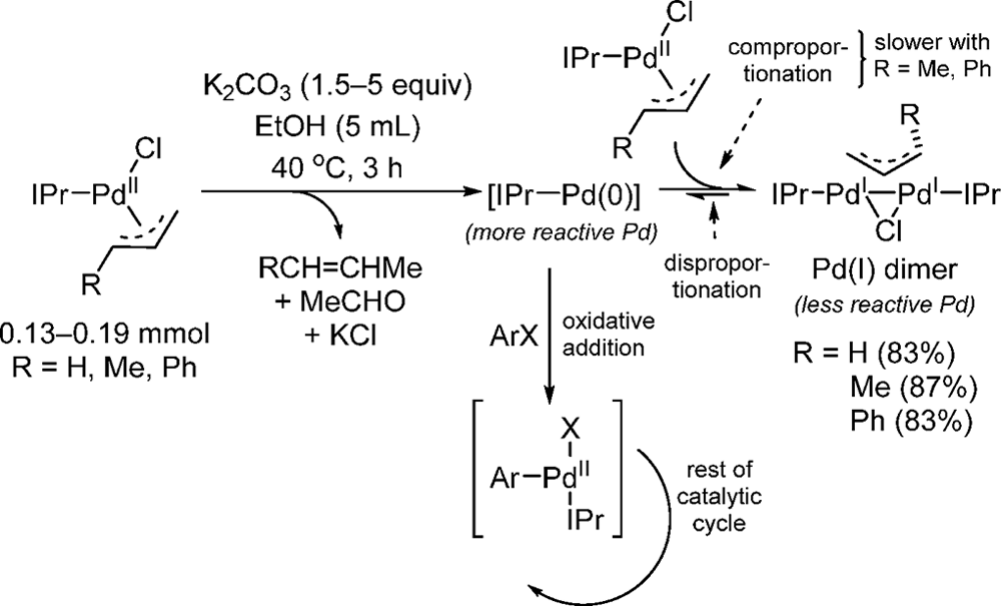
Balcells and Hazari’s Proposed Activation and Deactivation Pathways of Precatalysts of the Type Pd(η^3^-*R*-allyl)(NHC)Cl in the Suzuki–Miyaura Cross-Coupling

Balcells, Hazari, and co-workers carried out very detailed experimental and computational studies to understand the role of the Pd(I)(μ-allyl) dimer (“comproportionation” dimer) in catalysis by using NHC-based Pd systems.^195^ They were able to prove that the Pd(I)(μ-allyl) dimers are directly observed during catalysis in reactions that utilize Pd(II)-based Pd(allyl)(L)Cl precatalysts and concluded that Pd(I)(μ-allyl) dimer formation is detrimental because it removes the [IPr–Pd(0)] active species from the reaction mixture (Scheme 37).^195^ Their studies also clearly indicated that increased steric bulk at the 1 position of the allyl ligand in Pd(IPr)(η^3^-crotyl)Cl and Pd(IPr)(η^3^-cinnamyl)Cl results in a larger kinetic barrier to comproportionation. The slower rate of comproportionations in these two cases permits more of the active [IPr–Pd(0)] species to enter the catalytic cycle. Although Nolan’s, Verkade’s, and Colacot’s groups had noticed the effect of bulky substituents attached to the allyl group, Balcells and Hazari were able to establish the negative role of the “comproportionation” dimer during the catalyst activation process. They found that the increased catalytic activity of the (η^3^-1-R-allyl)Pd complexes was correlated to an increased barrier to dimer formation via comproportionation. In a related study of the effect of the electronic and steric properties of the C-2 substituent in precatalysts of the type Pd(η^3^-2-R-allyl)(IPr)Cl, Balcells and Hazari found that the catalytic efficiency of the precatalysts is inversely related to the thermodynamic stability of the corresponding (μ-2-R-allyl)-bridged Pd(I) dimers.^196^ Although (μ-allyl)-bridged Pd(I) dimers do function well as precatalysts in certain catalytic applications,^191,193,195^ dimer formation is generally a nonproductive off-cycle pathway, and disproportionation back to [L_1_Pd(0)] and the ligated allylpalladium(II) complex is required for catalytic activity.

#### 4.4.4 Catalyst Design Informed by the Mechanism of Precatalyst Activation

Guided by an understanding of the off-cycle pathway leading to the Pd(I)-based comproportionation dimer, and aiming to improve catalytic efficiency, Melvin et al.^199^ and DeAngelis et al.^187^ developed two entirely different approaches to designing precatalysts that would not form the undesirable dimer.

Nova, Hazari, and co-workers’ clever approach took advantage of the steric bulk inherent in the *tert*-butylindenyl motif to design and develop precatalysts of the type (η^3^-1-*t*-Bu-indenyl)Pd(L)Cl that do not form the corresponding inactive Pd(I) comproportionation dimers.^199^ The precatalysts are either produced in situ or are easily accessed from the precursor (η^3^-1-*t*-Bu-indenyl)_2_(μ-Cl)_2_Pd_2_ by reaction of the latter with a range of NHC or phosphine ligands, L. The higher activity observed for these precatalysts, when compared to the analogous ones generated from (η^3^-cinnamyl)_2_(μ-Cl)_2_Pd_2_ is attributed to the bulky *tert*-butylindenyl hindering construction of the chloride bridge necessary for Pd(I) dimer formation. To this point, when unsubstituted indenyl was used as the auxiliary and IPr as the ligand, the Pd(I) comproportionation dimer was isolated in 85% yield after treating the precatalyst with 2 equiv of K_2_CO_3_ in MeOH at rt for 2 h. In contrast, treating (η^3^-1-*t*-Bu-indenyl)Pd(IPr)Cl with 2 equiv of K_2_CO_3_ under the same conditions led to Pd(0) products such as Pd(IPr)_2_ and Pd black (the reactive [IPr–Pd(0)] is unstable in the absence of Ar–X and forms the Pd(0) products).

**Scheme 38:**
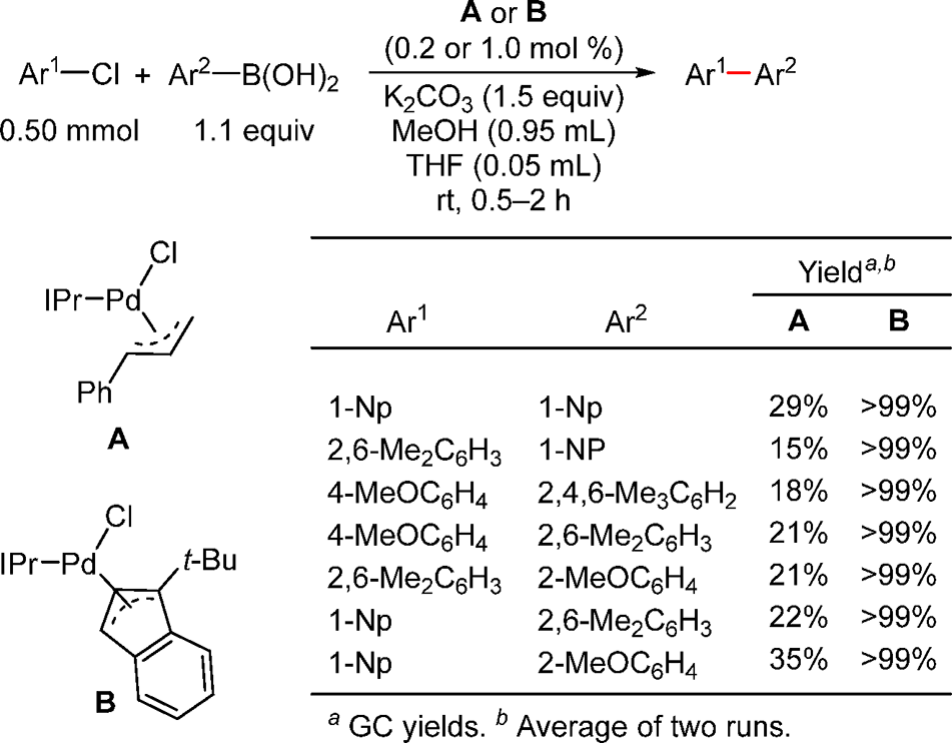
Selected Examples Highlighting the Superior Performance of (η^3^-1-(*tert*-butyl)indenyl)Pd(IPr)Cl Precatalyst vis-à-vis Its Cinnamyl-Supported Analogue, (η^3^-cinnamyl)Pd(IPr)Cl, in a Challenging Suzuki–Miyaura Cross-Coupling

The authors demonstrated the superior performance of these NHC-supported precatalysts vis-à-vis their cinnamyl-supported analogues in a challenging Suzuki–Miyaura cross-coupling (Scheme 38).^199^

**Scheme 39:**
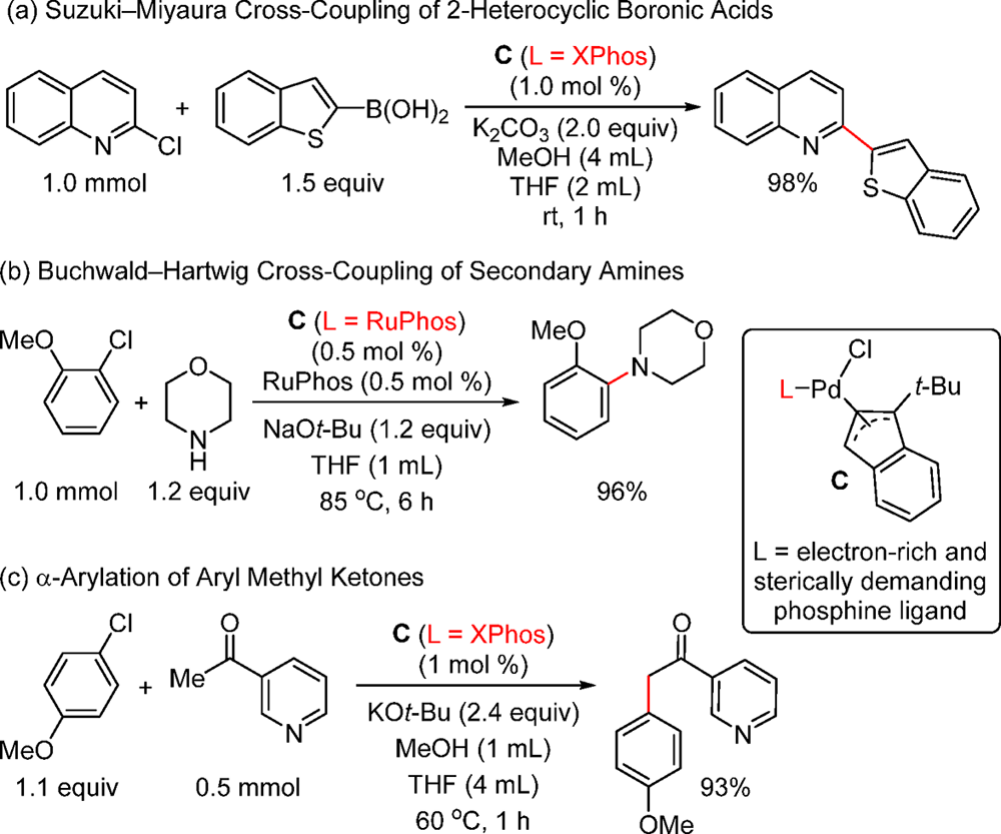
Representative Examples Showcasing the Superior Performance of Phosphine-Based (η^3^-1-(*tert*-butyl)indenyl)Pd(L)Cl (L = Phosphine Ligand) Precatalysts in a Number of Challenging Cross-Coupling Reactions

In addition to the (η^3^-1-*t*-Bu-indenyl)Pd(NHC)Cl precatalysts, Nova and Hazari’s team also successfully synthesized a series of complexes of the type (η^3^-1-*t*-Bu-indenyl)Pd(L)Cl, where L is an electron-rich and sterically demanding phosphine [L = SPhos, RuPhos, XPhos, DavePhos, P(*t*-Bu)_2_(4-Me_2_NC_6_H_4_), PPh_3_, P(*t*-Bu)_3_, QPhos, PCy_3_, and P(*o*-Tol)_3_].^199^ Similarly, these phosphine-based precatalysts did not form the inactive Pd(I) comproportionation dimer and proved highly active in a number of challenging cross-coupling reactions (Scheme 39).^199^

**Figure 9:**
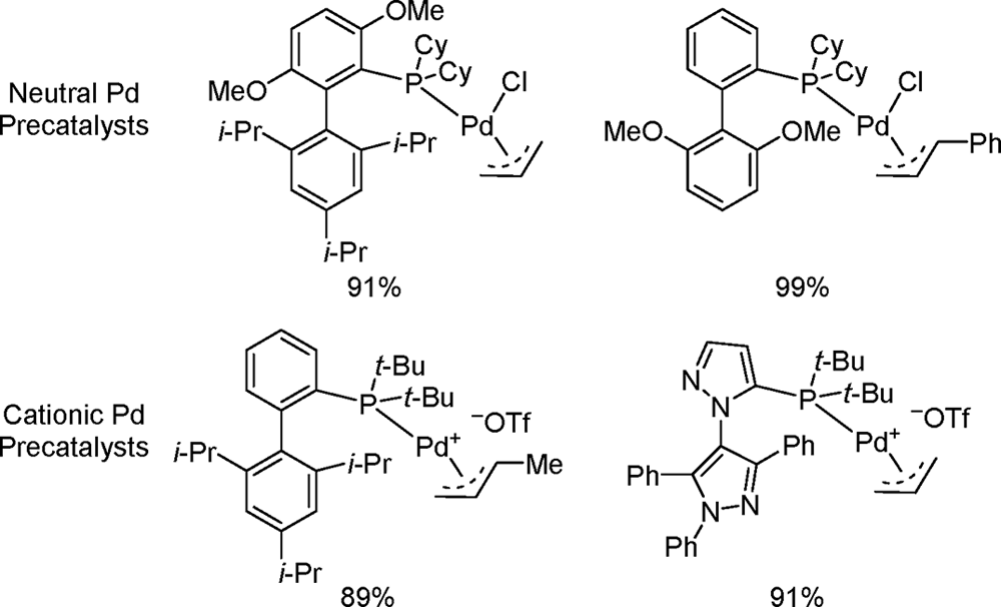
Selected neutral and cationic Pd(R-allyl)(L)X precatalysts (R = H, Me, Ph; X = Cl, TfO; L = biaryl ligand) synthesized and fully characterized.

**Figure 10:**
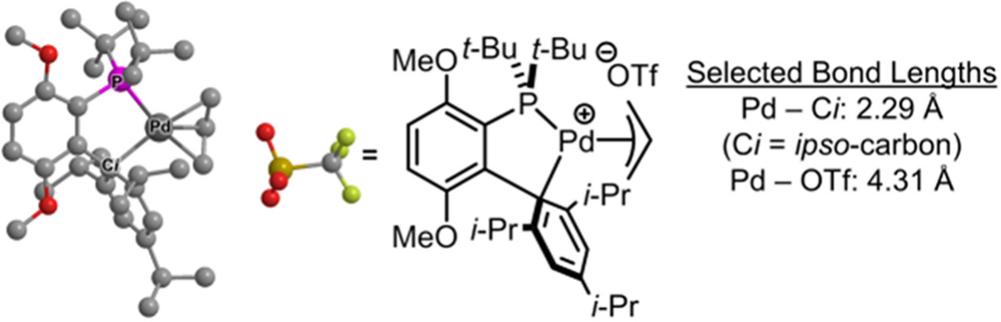
X-ray crystal structure of (π-allyl)(tBuXPhos)Pd(OTf) showing the presence of OTf out of the coordination sphere to accommodate the bulky tBuXPhos ligand. The corresponding chloride complex could not be formed due to the size of the tBuXPhos ligand. Reproduced with permission from ref (187). Copyright 2015 American Chemical Society.

Concurrently, Colacot’s alternative approach to minimizing or even preventing Pd(I) dimer formation involved using Buchwald-type biaryl ligands possessing larger cone angles than those of the conventional monophosphines.^187^ This research group carried out an extensive and systematic study of neutral Pd(R-allyl)(L)Cl complexes (R = H, Me, Ph; L = relatively less bulky biaryl ligand such as SPhos, RuPhos, XPhos, and BrettPhos). In the case of bulkier ligands, such as *t*BuXPhos, *t*BuBrettPhos, and AdBrettphos, precatalysts were designed and synthesized as cationic complexes in which the coordinating chloride anion was replaced with a noncoordinating anion such as TfO^–^. This substitution of the anion not only freed up more space in the coordination sphere of palladium, thus accommodating the bulkier ligands, but also prevented the formation of the “comproportionation” dimer (Figure 9).^187^ The X-ray crystal structure of one of these precatalysts is given in Figure 10.^187^

**Figure 11:**
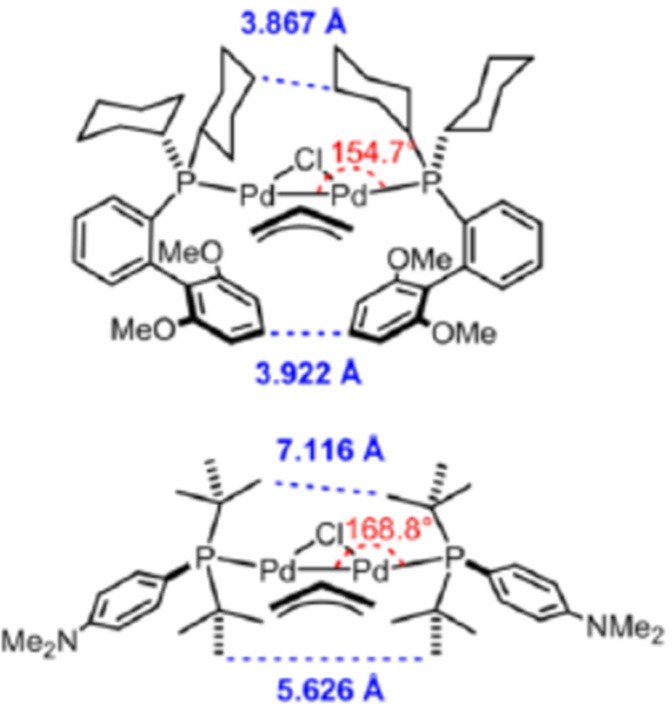
Structures of the Pd(I) dimers, Pd_2_(μ-allyl)(L_2_) (μ-Cl), with L = SPhos (top) and AmPhos (bottom). In the bottom structure, Pd(I) dimer formation is less favored based on a Pd–Pd–P bond angle of 168.8° (vs 154.7° for SPhos) and closest point of contact of the *tert*-butyl substituents of 5.626 Å (vs 3.922 Å for SPhos). Reproduced with permission from ref (187). Copyright 2015 American Chemical Society.

**Scheme 40:**
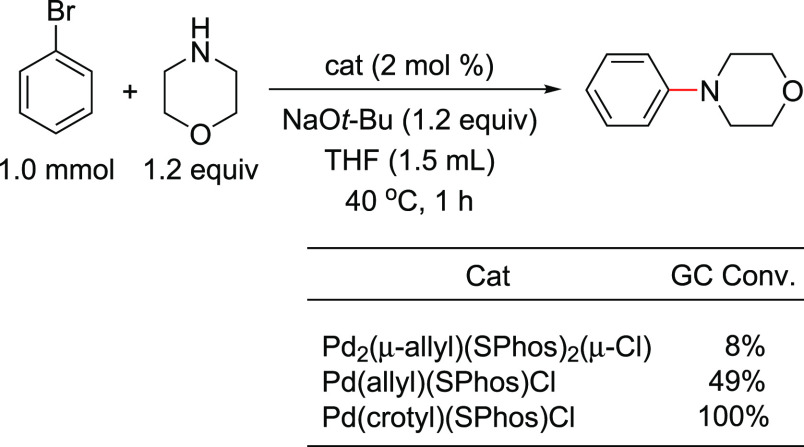
Even Allyl Complexes with Smaller Ligands Can Be Made More Catalytically Active by Preventing Pd(I) Dimer Formation

**Scheme 41:**
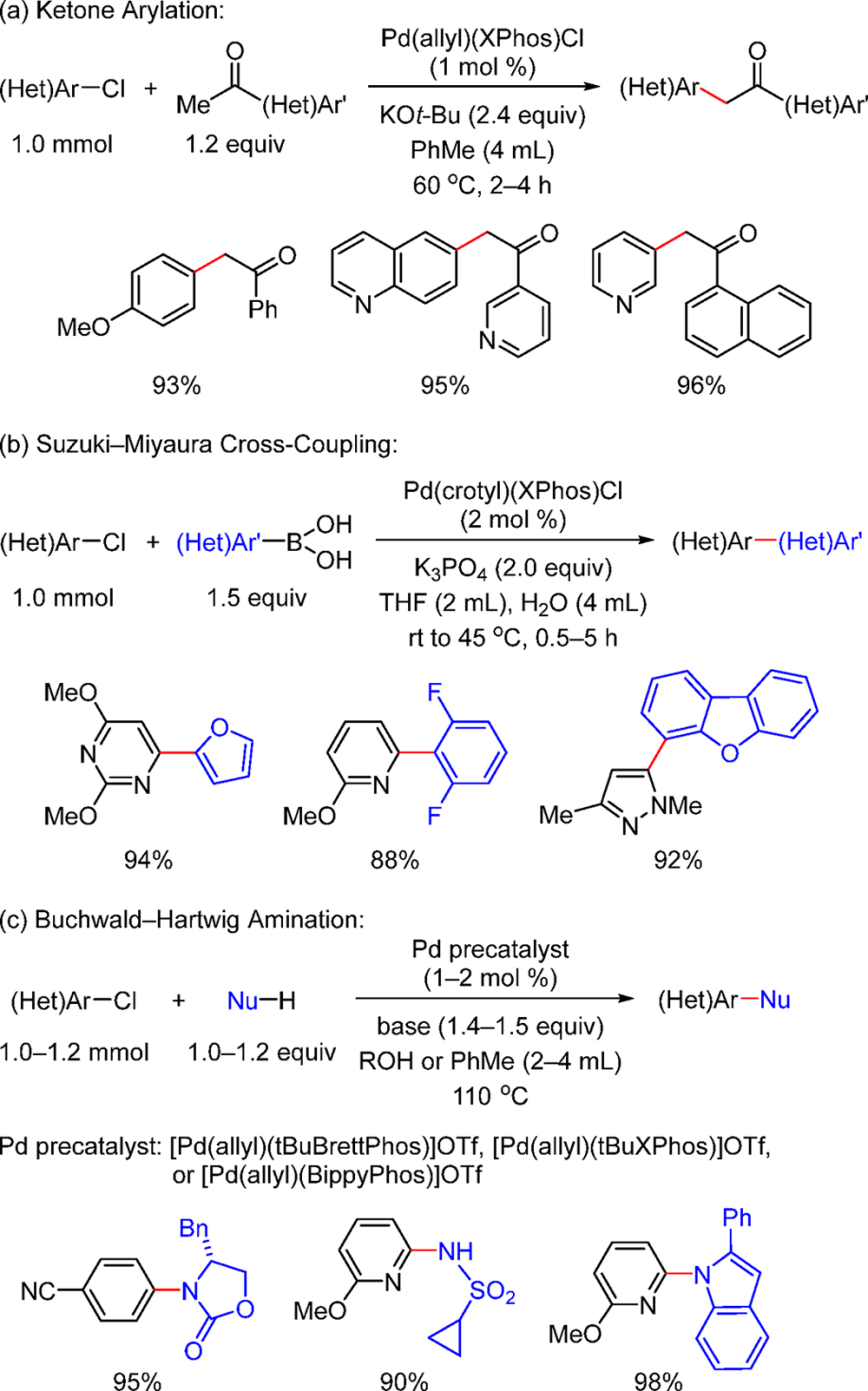
Representative Applications of Pd(R-allyl)(L)Cl and [Pd(allyl)(L)]OTf in Challenging Cross-Coupling Reactions

In this study, Colacot’s group demonstrated how to minimize the formation of the off-cycle allylPd(I) dimer by increasing the size of the R group on the allyl (similarly to Hazari’s work with the *t*-Bu-indenyl system) in conjunction with the size of the ligand, L, and, most importantly, by forming cationic complexes with a noncoordinating anion such as triflate, TfO^–^ instead of a bridging coordinating anion such as Cl^–^. The importance of the size of the ligand L in disfavoring formation of the Pd(I) dimer is seen by comparing the X-ray crystal structures of Pd_2_(μ-allyl)(L_2_)(μ-Cl), where L = (*t*-Bu)_2_(4-NMe_2_C_6_H_4_)P vs L = SPhos (Figure 11).^187^ Application studies confirmed that even allyl complexes with smaller ligands such as SPhos can be made active by this simple approach (Scheme 40).^187^ Examples of the applications of Pd(allyl)(XPhos)Cl and Pd(crotyl)(XPhos)Cl precatalysts in challenging ketone arylations, Suzuki–Miyaura cross-couplings, and Buchwald–Hartwig aminations are highlighted in Scheme 41.^187^

**Scheme 42:**
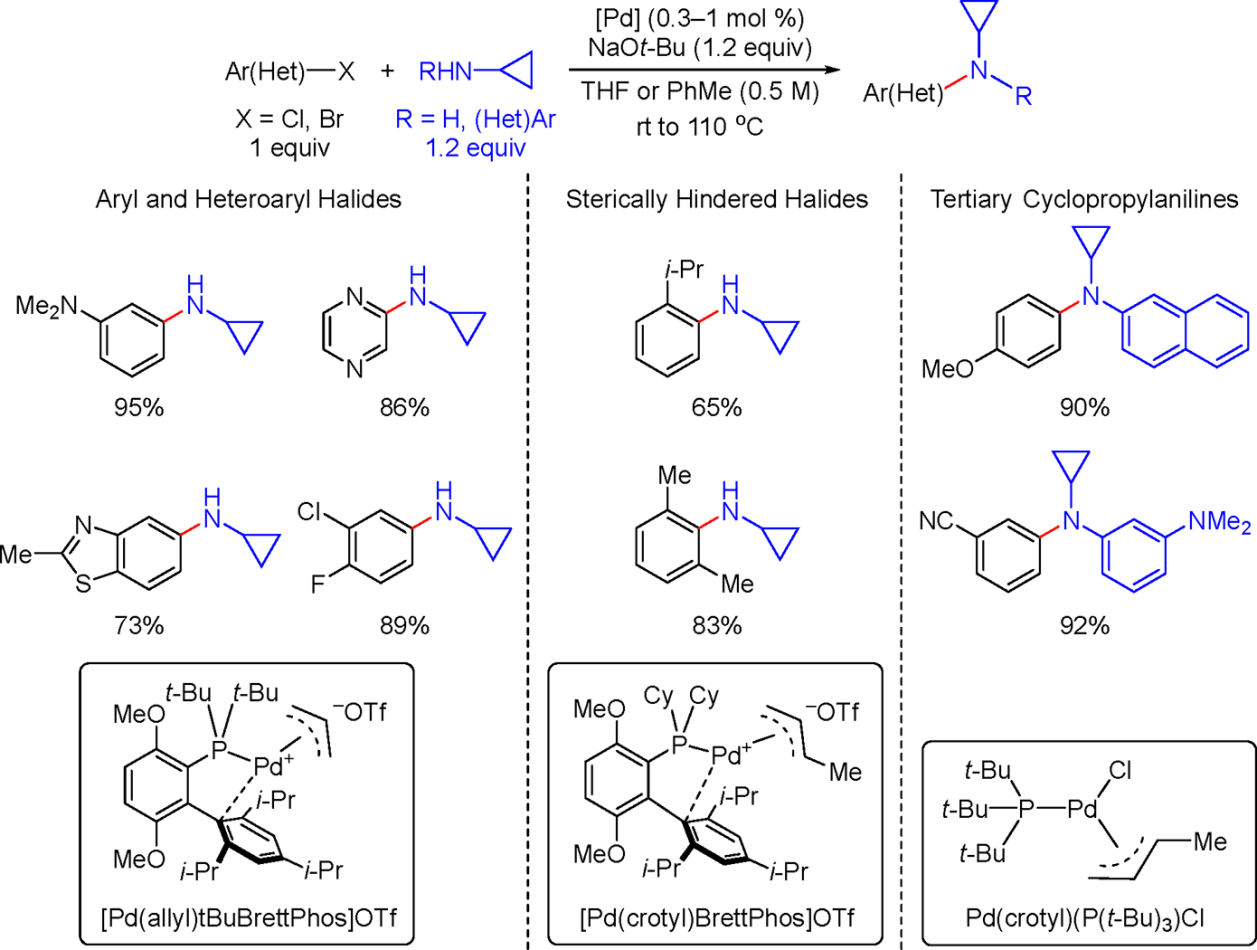
Examples of Challenging Mono- and Diarylations of Secondary and Primary Cyclopropyl Amines

It is worth mentioning in this context that, although the Buchwald–Hartwig amination is well established, the arylation of primary and secondary cyclopropyl amines had presented a significant challenge.^200−202^ Applying this approach, Gildner et al. successfully effected the mono- and diarylation of cyclopropyl amines using the Pd-based technology discussed in this section (Scheme 42).^203^ Subsequently, Stradiotto’s group developed a related nickel-based cyclopropylamine arylation using an *ortho*-phenylene-bridged bisphosphine bidentate ligand.^204^

**Scheme 43:**
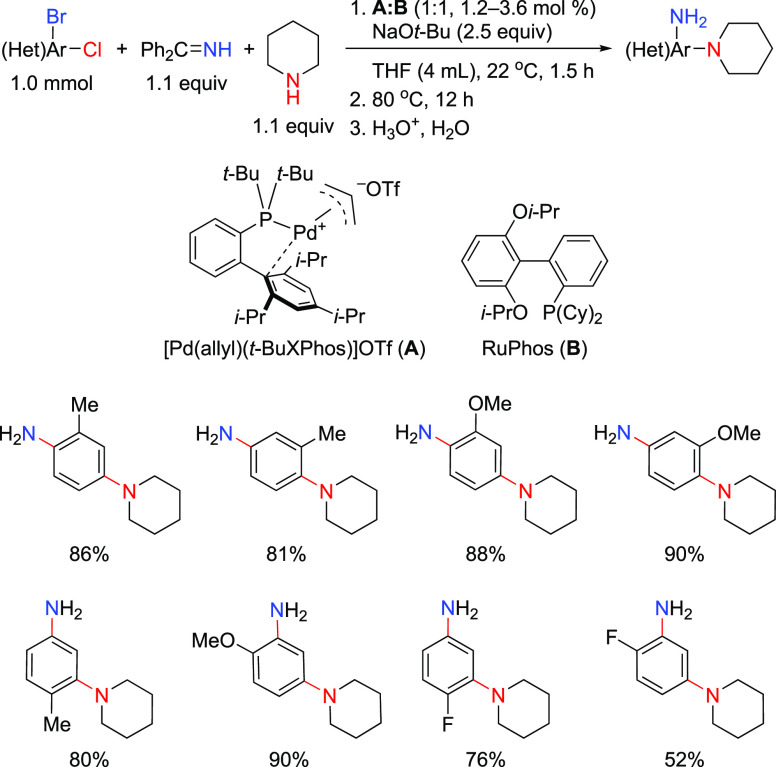
Three-Component, Chemoselective One-Pot Synthesis of Amine-Substituted Anilines Catalyzed by [Pd(allyl)(*t*-BuXPhos)]OTf in the Presence of RuPhos

In a related report, a tandem double amination protocol was described by Colacot and co-workers. The three-component, one-pot synthesis is catalyzed by [Pd(allyl)*t*-BuXPhos]OTf in the presence of RuPhos and provides amino aniline derivatives in high yields and chemoselectivity (Scheme 43).^205^ At room temperature, the chloro-substituted (hetero)aryl bromide couples with the benzophenone imine to give an aniline surrogate as an intermediate. Subsequent heating to ca. 80 °C, where ligand exchange presumably takes place, followed by hydrolysis provide the aniline derivative. This method is advantageous to keep the anilines protected, as some of them are susceptible to degradation with accompanying black color formation.

#### 4.4.5 PEPPSI-Type Catalysts

**Scheme 44:**
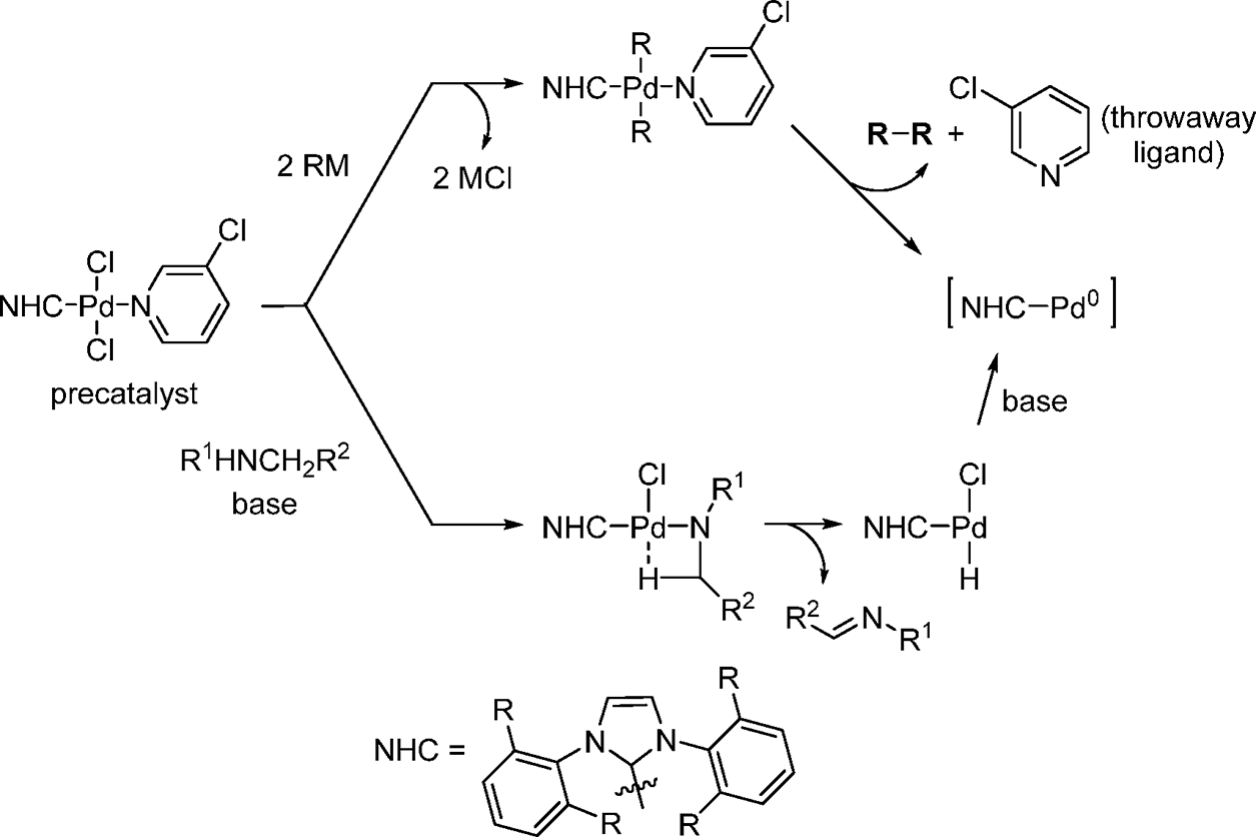
Activation of the PEPPSI Precatalyst to (NHC)Pd(0) through Reduction of Pd(II) to Pd(0) with an Organometallic or Amine Coupling Partner

In 2006, Organ and co-workers reported a unique class of NHC (N-heterocyclic carbene) based air- and moisture-stable Pd catalysts called pyridine enhanced precatalyst preparation stabilization and initiation (PEPPSI; Total Synthesis Ltd) that can generate monoligated LPd(0) upon activation by a suitable base.^206^ The PEPPSI precatalysts (commercially available from Sigma-Aldrich, now a part of Merck KGaA, Darmstadt, Germany)^207,208^ proved to have broad applications in cross-coupling reactions such as the Kumada, Negishi, Suzuki–Miyaura, Heck, Sonogashira, and Buchwald–Hartwig cross-couplings.^21,22^ The precatalyst, which has the general structure shown in Scheme 44, is synthesized by reacting an imidazolium salt with palladium chloride in the presence of K_2_CO_3_ and a pyridine ligand.^206,209^ These complexes are usually designated in short form based on the nature of the R group on the NHC unit; for example, PEPPSI-IMes, PEPPSI-IEt, PEPPSI-IPr, PEPPSI-IPent, and PEPPSI-IHept. However, Pd is sometimes used in front of the name.^209^ 3-Chloropyridine is a “throwaway” ligand that helps to stabilize the monoligated Pd by recoordination. The two chlorides cause the Pd to be in the +2 oxidation state, thereby making the complex stable to air and moisture. It is worth noting that the PEPPSI precatalyst requires an external reductant such as a strong nucleophilic coupling partner or a base to activate it to the [(NHC)Pd(0)] active state. The reduction mechanism is also shown in Scheme 44.

Early applications of the PEPPSI precatalysts were primarily in the cross-coupling of aryl halides and organometallic reagents such as organozincs. In these reactions, the organometallic reagent undergoes transmetalation with the precatalyst species to afford (NHC)PdR_2_(pyridine). Reductive elimination of R–R and dissociation of the pyridine ligand provide the active [(NHC)Pd(0)] species.^209^ In C–N coupling reactions of alkylamines, the amine can serve as the reductant through β-hydride elimination followed by deprotonation (see Scheme 44).^210,211^

Although Organ’s continuing contributions in this area are significant, newer versions of the PEPPSI precatalysts have also been developed by other groups by changing the sterics and electronics of the NHC ligands for specific substrates. For example, Nolan’s precatalyst, based on PEPPSI-IPr* (IPr is modified to IPr* by changing the isopropyl group to a diphenylmethyl group), exhibited superior activity in C–N coupling reactions in comparison to IPr- and SIPr-based PEPPSI precatalysts.^210^ Some of the important advances in the area of C–C and C–N couplings are only referenced here due to space limitations.^211−217^

#### 4.4.6 PEPPSI-Related NHC Catalysts

**Scheme 45:**
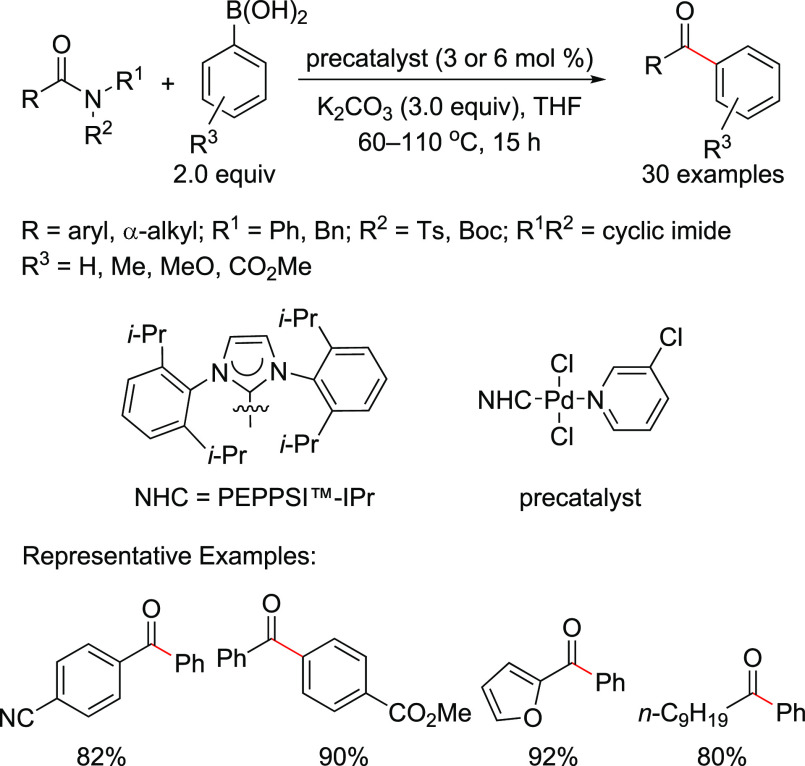
Szostak’s PEPPSI-IPr Assisted, Direct Suzuki–Miyaura Cross-Coupling of Aryl and α-Alkyl Amides with Arylboronic Acids to Produce Ketones

In addition to the conventional C–C and C–N coupling reactions, Szostak and co-workers reported PEPPSI-IPr as a highly active precatalyst in the direct Suzuki–Miyaura cross-coupling of a wide range of amides as substrates with various arylboronic acids to produce ketones in very good yields (Scheme 45).^218^ The same group employed IPr(cinnamyl)PdCl as a precatalyst for the same transformation just prior to this work and claimed that the NHC systems are superior to the PR_3_ systems.^219^

**Scheme 46:**
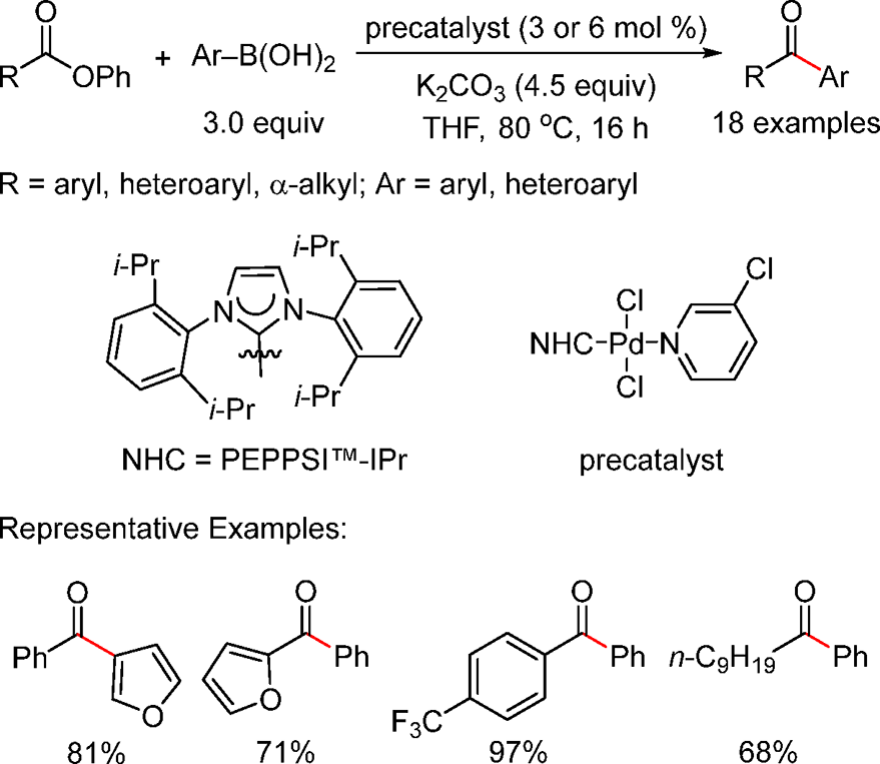
Szostak’s Application of PEPPSI-IPr to the Suzuki–Miyaura Coupling of Esters with Boronic Acids Leading to Ketones

Subsequently, Szostak’s group expanded the work to esters as substrates, where C–O bond cleavage takes place to form ketones under the Suzuki–Miyaura cross-coupling conditions (Scheme 46).^220,221^

**Figure 12:**
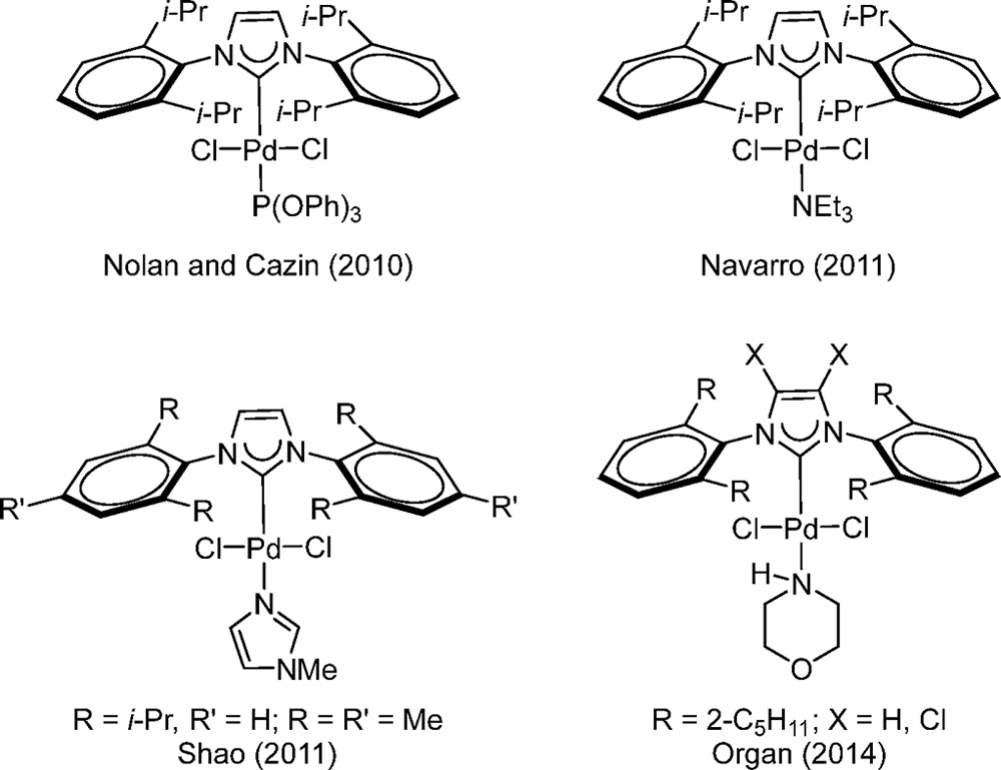
PEPPSI-type precatalysts containing “throw-away” ligands.

The same year, Szostak’s group reported a Pd-PEPPSI-IPr catalyzed Buchwald–Hartwig coupling of both common esters and amides via a highly selective C(acyl)–X (X = O, N) bond cleavage to rapidly access a variety of aryl amides.^222^ Very recently, the same research group disclosed a Pd-PEPPSI-IPr catalyzed Suzuki–Miyaura cross-coupling of *N*-acylcarbazoles and *N*-acylindoles with arylboronic acids by a highly selective N–C(O) bond cleavage to produce aryl ketones in moderate-to-excellent yields.^223^ To improve the activities of the Pd-PEPPSI precatalyst in coupling reactions, Nolan and Cazin,^224^ Navarro,^225^ Shao,^226,227^ and Organ^228^ have reported on efforts to replace 3-chloropyridine in Pd-PEPPSI with other throwaway ligands such as P(OPh)_3_, Et_3_N, methylimidazole (MeIm), and morpholine (Figure 12).

For example, Navarro’s precatalyst is more active at 25 or 50 °C than the corresponding PEPPSI-IPr in both the Suzuki–Miyaura and Buchwald–Hartwig cross-couplings of aryl chlorides.^225^ This higher activity might be due to the facile dissociation of triethylamine (TEA) to generate the [L_1_Pd(0)] species or the facile recoordination of TEA to the Pd(0) species, thereby imparting more stability to the Pd catalyst during the catalytic cycle.^225^ Organ’s morpholine adducts of IPent and IPent^Cl^ precatalysts provided a similarly efficient reduction pathway for catalyst activation through a β-hydride elimination.^228^ The IPent NHC, with morpholine as the throwaway ligand, gave a 96% yield in the C–S coupling of 1-chloro-2,6-dimethylbenzene with thiophenol at room temperature, while the highly active PEPPSI-IPent catalyst gave no conversion under these conditions.^228^ Nolan and Cazin’s precatalyst with P(OPh)_3_ as the throwaway ligand was also effective at rt in the Suzuki–Miyaura coupling of aryl chlorides, although an alcohol was required to reduce Pd(II) to [(NHC)Pd(0)] with the formation of acetone.^224^ These studies clearly reveal that a weak ligand is required to break up the dimer, [(NHC)PdCl_2_]_2_, in order to form a monomeric tetracoordinate Pd as the precatalyst. It is important to note that [(NHC)PdCl_2_]_2_ can also act as a precatalyst to generate [(NHC)Pd(0)], but it is less active than the PEPPSI-type complexes; although there have not been enough control experiments. Because of space limitation, the published work in this area has not been covered in detail; the interested reader should consult the relevant reviews and book chapters for further insight into this area.^15−20,229−234^

#### 4.4.7 Oxidative Addition Complexes L_1_Pd(Ar)X as Monoligated Pd

Several examples of palladium(II) oxidative addition complexes, Pd(II)OACs, have been synthesized, isolated, characterized, and employed as precatalysts in mechanistic and kinetic studies.^235−246^ In this section, we shall focus only on the applications of L_1_Pd(Ar)X as efficient isolable or in situ generated precatalysts that are valuable in a number of organic transformations.

As discussed in the section on mechanisms (section 2), transition-metal-catalyzed cross-coupling reactions involve three elementary steps, with the oxidative addition being the first step (see Scheme 1).^37^ Although oxidative addition of a d^10^ metal center and an aryl halide or pseudohalide can occur through three possible pathways (radical, S_N_Ar, and 3-center 2-electron), Pd(0)/Pd(II) catalytic cycles generally involve a 3-center 2-electron transition state to yield oxidative addition complexes of the general formula LPd(Ar)X as intermediates.

As discussed in previous sections, studies have shown that the generation of catalytically active and coordinatively unsaturated monoligated Pd(0) species is crucial for the success of modern cross-coupling reactions. From mechanistic studies, it is well understood that L_1_Pd(II) species are involved in all three catalytic steps, namely oxidative addition, transmetalation, and reductive elimination (see section 2). While the [L_1_Pd(0)] species have not been isolated (with the exception of Carrow’s work in detecting them),^137^ the air-stable OACs such as L_1_Pd(II)(Ar)X, have been isolated and used as precatalysts that can lead to [L_1_Pd(0)]. In one of the earlier applications of a Pd(II)OAC, (*t*-Bu_3_P)Pd(Ph)Br was used as a Pd(0) precatalyst to carry out a chain-growth polymerization via the Suzuki–Miyaura coupling in the synthesis of polyfluorene.^247^

**Scheme 47:**
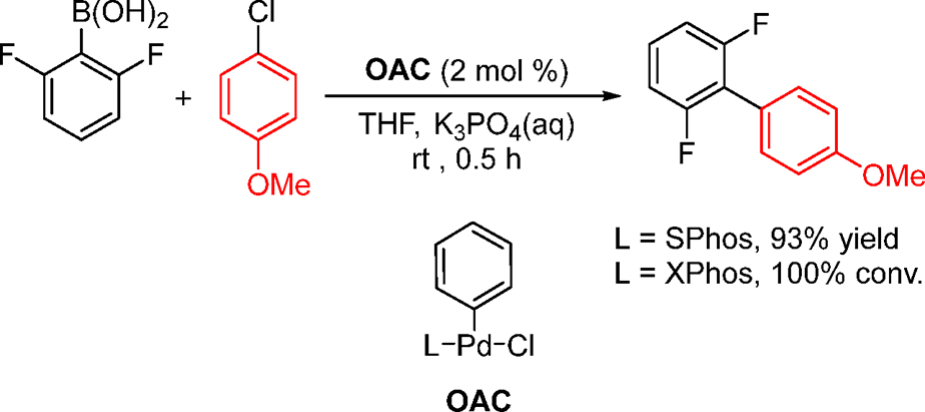
Buchwald’s Successful Application of a Palladium Oxidative Addition Complex (OAC) as a Precatalyst in a Fast Suzuki–Miyaura Cross-Coupling of Rapidly Deboronating 2,6-Difluorophenylboronic Acid with 4-Chloroanisole

Pioneering work by Buchwald’s group uncovered the potential of oxidative addition complexes (SPhos)Pd(Ph)Cl and (XPhos)Pd(Ph)Cl as precatalysts for a rapid Suzuki–Miyaura coupling of unstable polyfluorophenylboronic acids (Scheme 47).^185^ After demonstrating the stoichiometric reaction of 2,4-difluoroboronic acid with 4-chloroanisole using SPhos G6, the catalytic reaction was performed in a mixture of THF and 0.5 M aqueous K_3_PO_4_ (1:2 ratio) to obtain 93% of the coupled product within 30 min at room temperature. Increasing the reaction time did not improve the yield but resulted in protodeboronation, while increasing the temperature also gave a lower conversion. Complete conversion of 4-chloroanisole at room temperature was achieved by using XPhos G6 instead of SPhos G6. Although this was a significant result for the challenging Suzuki–Miyaura coupling involving time- and temperature-sensitive boronic acids, the authors commented at that time *“the preparation and isolation of an individual oxidative-addition complex for each substrate is clearly impractical and often impossible”*.^185^

Related work from Buchwald’s group has described the reductive elimination of L_1_Pd(Ar)F (L = bulky Buchwald ligand) for aryl fluoride synthesis.^248^ Using L = BrettPhos, this work unambiguously demonstrated that reductive elimination of ArF from Pd(II) centers is feasible in stoichiometric reactions. Subsequently, Buchwald and co-workers designed and developed new ligands, new precatalysts, and new reaction conditions to efficiently perform catalytic aryl fluorinations.

**Scheme 48:**
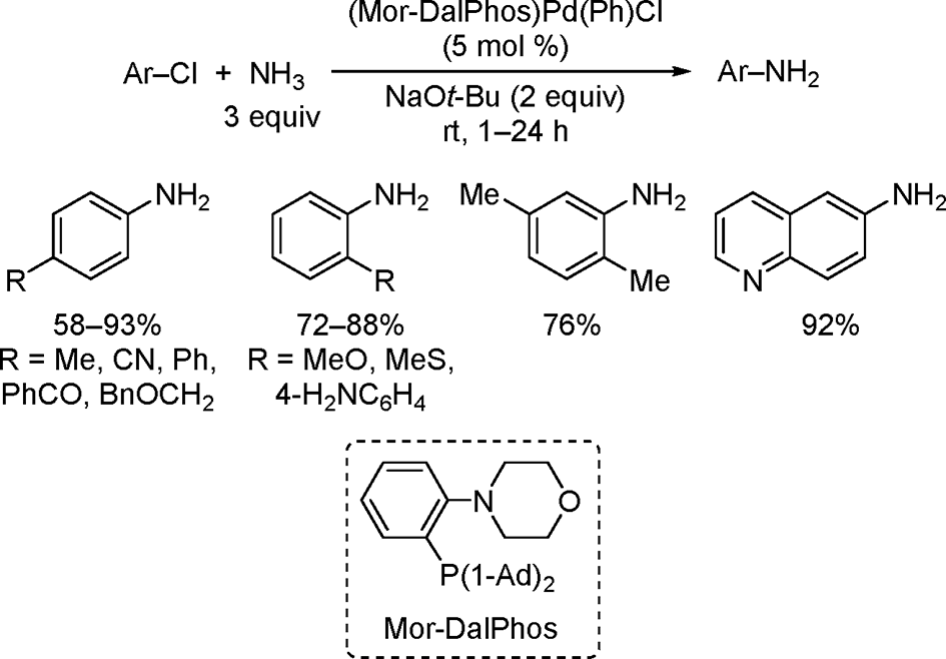
Stradiotto’s Application of a Mor-DalPhos-Based Pd(II) Oxidative Addition Complex as a Precatalyst in a Rapid and Mild Arylation of Ammonia with Deactivated Aryl Chlorides

The same year, Stradiotto’s group prepared OAC, L_1_Pd(Ar)Cl by using L = Mor-DalPhos and characterized it by single-crystal assays to be a square planar complex via N-coordination of the morpholine. However, the basic structure resembles the T-shaped geometry of oxidative addition complexes. The group also tested this as a precatalyst for the direct arylation of ammonia with deactivated aryl chlorides (Scheme 48).^249^

Later, Buchwald and co-workers demonstrated the broad applicability of L_1_Pd(Ar)X complexes as precatalysts,^250^ in which L_1_ is a Buchwald ligand, for C–C,^185^ C–N,^251−254^ C–O, and C–F,^251^ as well as C–S^255^ bond formation. These complexes are generally referred to as Buchwald Pd G6 precatalysts, and they share similar advantages with the prior generations of Buchwald precatalysts: quantitative generation of [L_1_Pd(0)]; air, moisture, and thermal stability, ease of handling, and high reaction efficiency.

In addition, the Pd G6 precatalysts offer several comparative advantages over the previous generations of Buchwald precatalysts. First, catalyst activation does not require a base and generates innocuous byproducts (as opposed to carbazole inhibitors as byproducts). Second, the Buchwald Pd G6 precatalysts are OACs, which means they are “on-cycle” intermediates, and typically provide higher reactivity and selectivity. Third, Buchwald Pd G6 precatalysts are prepared in a single step at room temperature. Fourth, the synthesis of Buchwald Pd G6 precatalysts allows for a versatile and tunable precatalyst design: (i) Each of the three ligands (L, Ar, X) can be independently fine-tuned. (ii) Improved solubility, greater stability, increased reactivity, and/or easier purification can be achieved by judicious selection of X, L, and Ar. (iii) Bulky ligands (e.g., L = *t*-BuBrettPhos, AdBrettPhos, and AlPhos) are easily accommodated in the structure of the precatalyst. While these incremental improvements in precatalyst technologies can be likened to the incremental improvements in smart phone technologies,^101^ the syntheses of G6 catalysts are relatively more difficult to scale up for commercial use and hence process chemists need to carefully weigh the selection of one catalyst over another.

**Scheme 49:**
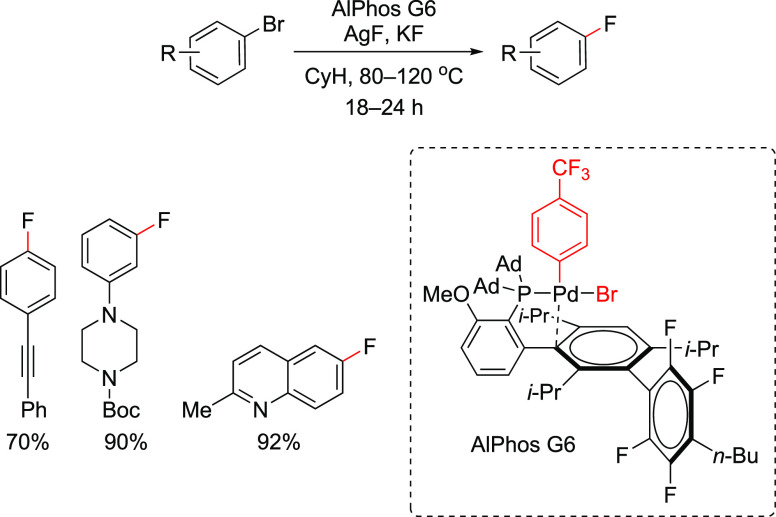
Applications of AlPhos G6 for the Effective Fluorination of Aryl Bromides

Ingoglia and Buchwald have described the synthesis of the oxidative addition complexes of very bulky biaryl ligands such as *t*-BuBrettPhos and AlPhos and demonstrated their applications as effective precatalysts for C–N, C–O, and C–F cross-coupling reactions.^251^ This technology is a convenient alternative to the previously developed technologies utilizing G1–G5 precatalysts, particularly in the case of the bulkiest biarylphosphine ligands, for which palladacycle-based precatalysts are difficult to isolate. The advantages of this technology are exemplified by the unique applications of AlPhos G6 to the effective fluorination of aryl halides and triflates (Scheme 49).^251^

Encouraged by these results, Cernak, Buchwald, and co-workers devised an alternative approach for carrying out Pd-catalyzed cross-couplings of densely functionalized molecules by using stoichiometric quantities of palladium OACs as substrates. These OACs were formed from drugs or drug-like aryl halides. In most cases, these stoichiometric cross-couplings gave better results under milder conditions than their catalytic counterparts. The OACs are remarkably stable under ambient conditions, maintaining their reactivity after months of storage on the benchtop. These workers validated the utility of OACs in various reactions, including automated nanomolar scale couplings between a rivaroxaban-derived OAC and hundreds of diverse nucleophiles and in the late-stage derivatization of the natural product k252a.^256^

Carrow and co-workers took a similar approach by utilizing (PAd_3_)Pd(4-C_6_H_4_F)Br as a highly efficient precatalyst for the room-temperature Suzuki–Miyaura coupling of aryl bromides and base-sensitive polyfluorinated arylboron nucleophiles that are very prone to protodeboronation.^257,258^ The study claims that this unique catalyst system is superior in terms of efficiency to the in situ generated catalysts formed from PAd_3_ and Pd_2_(dba)_3_ and to other precatalysts such as (PAd_3_)Pd(η^3^-cinnamyl)Cl, [(P(*t*-Bu)_3_)PdBr]_2_, SPhos G2, XPhos G3, and IPr PEPPSI, thereby demonstrating the unique role of PAd_3_ as ligand in the G6 technology.

**Scheme 50:**
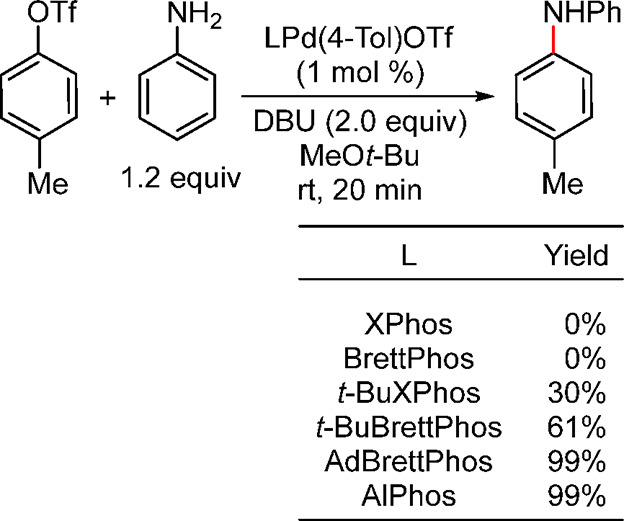
Mild-Base-Assisted C–N Coupling Using L G6, Demonstrating the Dramatic Effect of the Ligand on the Outcome of the Cross-Coupling

Typically, the Buchwald–Hartwig coupling requires harsh inorganic bases; however, by choosing the appropriate ligand of the G6 system or (cod)-coordinated (LPd)_2_, milder organic bases such as DBU can be used (Scheme 50).^252^

The same group expanded the scope of the work further to C–S cross-couplings at room temperature in the presence of soluble bases by using a G6-based biaryl ligand system.^255^ Subsequently, a collaboration between the Buchwald and Jensen groups at MIT’s chemistry and chemical engineering departments involved the utilization of an automated microfluidic optimization platform to determine the optimal reaction conditions for the cross-coupling of an aryl triflate with four types of commonly employed amine nucleophiles: anilines, amides, and primary and secondary aliphatic amines.^253^ By analyzing trends in catalyst reactivity across different reaction parameters, such as temperature and base concentration, they were able to develop a set of general protocols for C–N cross-couplings that rely on organic bases. The optimization algorithm revealed that AlPhos G6 was the most active system in the coupling of each amine nucleophile. Furthermore, their automated optimization showed that the phosphazene base BTTP [*tert*-butylimino-tri(pyrrolidino)phosphorane] could be employed to assist the coupling of secondary alkylamines with aryl triflates.

**Scheme 51:**
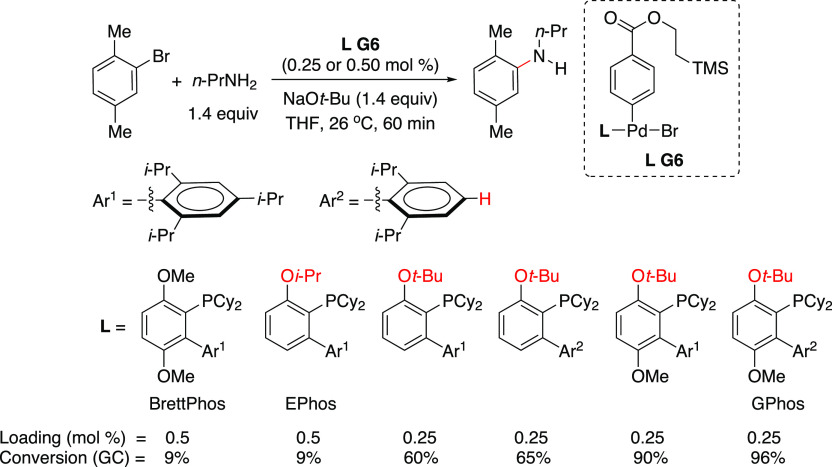
Reactivity of G6 Precatalysts Supported by BrettPhos-Derived Ligands, in Particular GPhos G6, in the Room-Temperature Amination of Aryl Halides Using NaO*t*-Bu

Very recently, Buchwald’s group modified BrettPhos by introducing minor alterations in the biaryl group (replacing the MeO group ortho to PCy_2_ with *t*-BuO and even replacing the *i*-Pr group at C′-4 with hydrogen), resulting in a new, monophosphine ligand, GPhos. The G6 complex of GPhos was utilized for the primary amination of aryl halides with loadings as low as 0.25 mol % (NaO*t*-Bu, THF, rt), and its activity was compared to those of Brettphos G6 and other new versions of G6 complexes (Scheme 51).^254^ This work clearly demonstrates how one can improve the efficiency of the Buchwald–Hartwig amination, even when using inorganic butoxide base at rt, by the judicial choice of ligand and precatalyst.

**Scheme 52:**
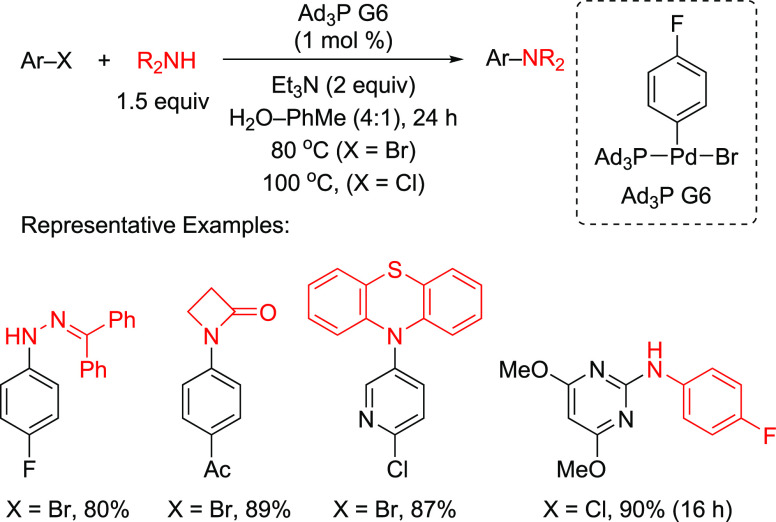
Carrow’s Application of Ad_3_P G6 in the Water-Assisted C–N Cross-Coupling Using the Mild and Soluble Base Et_3_N

In the same vein, Carrow and co-workers utilized an Ad_3_P G6 system to effect Buchwald–Hartwig aminations under mild conditions in which the beneficial roles that H_2_O plays were also highlighted. Thus, the cross-coupling of aryl amines, amides, and secondary amines with aryl bromides and chlorides was achieved in the presence of the weak, soluble base Et_3_N (Scheme 52).^259^ The advantage of this technology is that the scope of the C–N coupling can be expanded to substrates with base-sensitive functional groups and that it can be more suited for flow chemistry where the handling of solids is a major impediment.

Using SPhos G6, research groups at Cornell and BASF jointly demonstrated that the halide salt, formed as a byproduct in the cross-coupling reaction, causes the transmetalation step to be reversible, and leads to strong reaction inhibition in the case of (hetero)aryl iodides. Kinetic and stoichiometric studies showed that halide inhibition likely results from the formation of the highly reactive Pd–OH intermediate being disfavored. By changing the solvent in the biphasic reaction system from THF to toluene, this inhibition was effectively minimized. The study also revealed that inhibition by halide is likely a more general problem in metal-catalyzed cross-coupling reactions, in particular the ones that involve a reversible transmetalation step.^260^

In a joint effort by the Pentelute and Buchwald groups, OACs were employed for the modification of complex biomolecules via cysteine bioconjugation. Key features of the Pd(II)OACs employed are their ease of preparation, storage, and handling. In particular, these reagents enabled the synthesis of new classes of stapled peptides and antibody–drug conjugates.^261−263^

The synthesis of disulfide-containing polypeptides has been a long-standing challenge in peptide chemistry, and versatile methods for the construction of disulfides are always in demand. Furthermore, a limited number of strategies are known for on-resin formation of disulfides directly from their protected counterparts. Recently, Stockdill and co-workers disclosed a novel peptide modification method, whereby Pd-mediated on-resin disulfide formation proceeds directly from the protected peptide without loss of any acid-labile side chain protecting group.^264−266^

Pd(II)OACs could prove highly valuable in drug discovery efforts as demonstrated by Cernak, Buchwald, and co-workers, who developed a practical cross-coupling method that applies to densely functionalized targets, as would be required in late-stage diversification of pharmaceuticals and other biologically active compounds.^256^ These workers generated a library of stable, isolable, and easy-to-handle Pd(II)OACs from complex, drug-like aryl halides that can lead to the formation of C–C (alkylation and alkynylation), C–N, and C–S bonds, and to cyanation as well as carbonylative amination. While the substoichiometric reaction generally showed low-to-no conversions, the stoichiometric reaction provided moderate-to-high conversions.

#### 4.4.8 Applications of OACs as Tools for Mechanistic and Kinetic Studies

There are many examples in which arylpalladium(II) halide complexes are employed as tools for mechanistic and kinetic studies. Seminal work by Hartwig has provided a better understanding of the factors that affect the reversibility of the oxidative addition of Pd(0) to ArX.^267−269^ In particular, Hartwig showed that Pd(II)OACs of the type Pd(Ar)(L)X (Ar = *o*-Tol, L = P(*t*-Bu)_3_, and X = I, Br, and Cl) undergo reductive elimination to regenerate the starting ArX under certain reaction conditions induced by the addition of a ligand and reaction temperature.^269^ This study led to the following noteworthy conclusions: (i) While monomeric, three-coordinate arylpalladium(II) halide complexes had previously been proposed for cross-coupling reactions utilizing sterically demanding phosphine ligands,^108,270^ this was the first time these species were isolated for study.^269^ (ii) Reductive elimination of ArCl is more thermodynamically favorable than reductive elimination of ArBr and ArI, reflecting the Ar–X bond strength. (iii) The observed kinetics show that ArBr eliminates faster than ArCl due to the stability of the palladium haloarene intermediate.^269^ (iv) A ligand screen highlighted the positive impact of steric bulk on the efficiency of the reductive elimination.

**Scheme 53:**
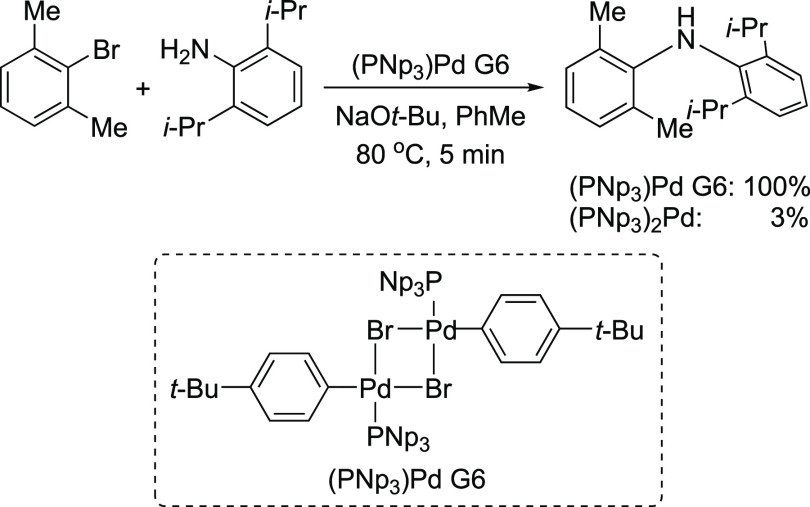
Shaughnessy’s Buchwald–Hartwig Amination of Aryl Halides with Anilines

In 2017, Shaughnessy and co-workers studied the mechanism of the Buchwald–Hartwig amination of aryl halides with anilines utilizing [(PNp_3_)Pd(Ar)(μ-X)]_2_ as the Pd G6 precatalyst (Scheme 53).^54,55^ The reaction of sterically hindered aryl bromides with derivatives of anilines occurred within 5 min, in contrast to that utilizing the L_2_Pd(0) based Pd(PNp_3_)_2_ system which required ca. 1 h at 80 °C for the same conversion with 1 mol % loading. These results offered a direct comparison of the turnovers (specifically TOFs) associated with the facile generation of [L_1_Pd(0)] species from the G6 complexes versus that from the L_2_Pd(0) complexes. They also indicated that the type of aryl halide, the steric demand of the aryl halide and aniline, and the choice of ligand affect reaction efficiency.

## 5 Aqueous-Phase Catalysis Using L_1_Pd(0) Species

**Figure 13:**
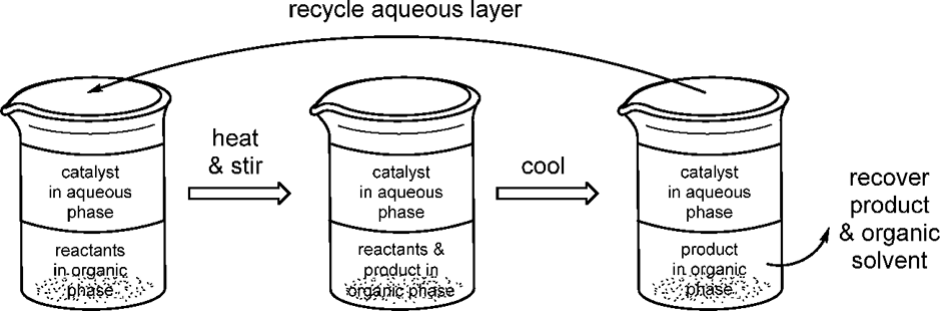
Schematic of aqueous biphasic catalysis.

The use of water as a solvent in organic synthesis for the large-scale application of green chemistry technology has been known for a few decades and is well documented.^271^ In the early days, water was introduced as part of a biphasic system, and the organometallic catalysts (preformed or generated in situ) employed were engineered to be water-soluble to interact with the reactants, while the products formed migrated to the organic layer and got separated (Figure 13). The catalyst in the aqueous phase was typically recycled, and the number of recycles depended on the life of the catalytic species. Commonly, these catalyst systems were simple metal salts with or without phosphine or related ligands and in which the ligands were typically made water-soluble by attachment of polar functional groups such as sulfonate, carboxylate, ammonium, phosphonium, or hydroxyl.^272,273^

Kobayashi’s recent review highlights the importance of creating a sustainable society by carrying out organic reactions in water, even though many reactants and catalysts are incompatible due to their immiscibility and/or degradation in water.^274^ Kobayashi states that, “After the “watershed” in organic synthesis revealed the importance of water, the development of water-compatible catalysts has flourished, triggering a quantum leap in water-centered organic synthesis”. He goes on to say, “Given that organic compounds are typically practically insoluble in water, simple extractive workup can readily separate a water-soluble homogeneous catalyst as an aqueous solution from a product that is soluble in organic solvents”.

### 5.1 Water-Soluble Ligands and Catalysts

Although numerous examples exist of water-soluble ligands and catalysts that are suitable for cross-coupling applications, this review will focus only on precatalysts that give rise to L_1_Pd(0) catalytic systems. This is important, as L_1_Pd(0) systems are expected to be extremely reactive to air and presumably to moisture under normal conditions. The following examples are intended to highlight the practicality of these catalytic systems in cross-coupling reactions.

#### 5.1.1 PEPPSI-Type Soluble Catalysts

**Scheme 54:**
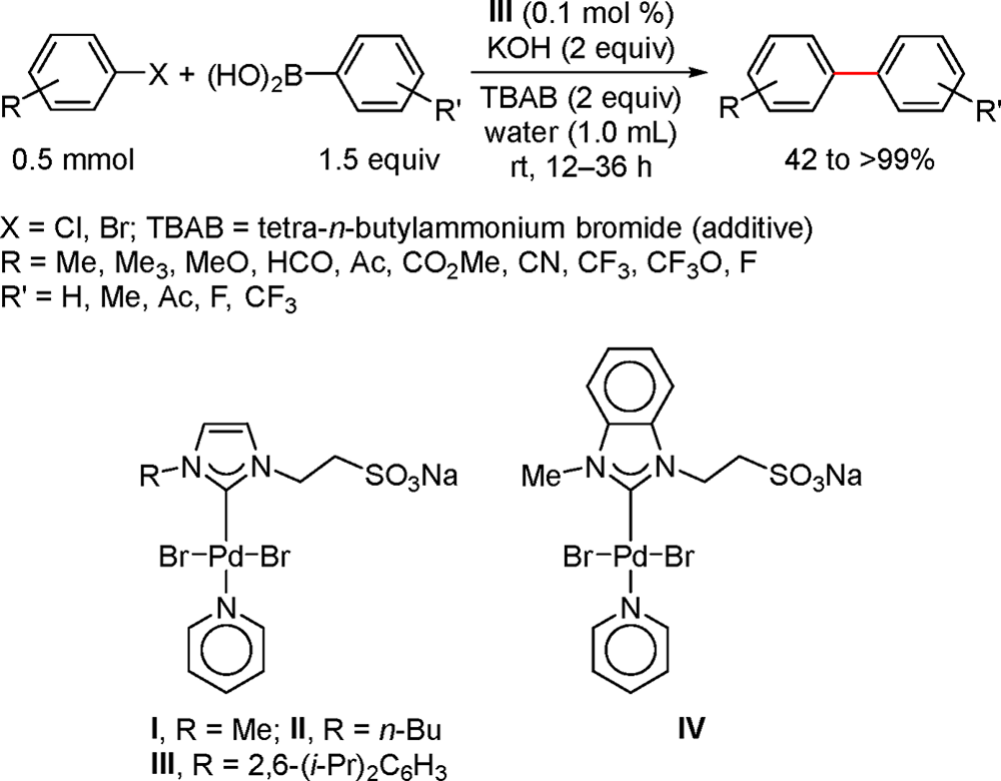
Water-Soluble, NHC-Based Precursors of L_1_Pd(0) for the Suzuki–Miyaura Cross-Coupling in Water and in Air

Examples of N-heterocyclic carbene (NHC) based precatalysts made water-soluble by incorporation of an SO_3_Na functional group were disclosed by Pöthig, Kühn, and co-workers in 2014 (Scheme 54).^275^ These PEPPSI-type precatalysts were utilized in the Suzuki–Miyaura cross-coupling at room temperature in water and in air. Palladium complex **III** exhibited the best catalytic activity in the cross-coupling of aryl bromides with boronic acids at a low catalyst loading of 0.1 mol %. Complex **III** could be recycled at least four consecutive times without significant loss of activity, thereby reducing the effective loading to as low as 0.025 mol %. Its higher catalytic activity was attributed to the bulky and electron-rich isopropyl groups on the benzene ring attached to the NHC. Based on TEM analysis and kinetic and mercury poisoning experiments, the authors posited that Pd nanoparticles formed during the reaction, presumably from L_1_Pd(0), are responsible for the observed catalytic activity.

A new air- and moisture-stable PEPPSI-type complex, [Pd(L)Br_2_(Py)] [L: 3-(2-fluorobenzyl)-1-(4-methoxyphenyl)-1*H*-imidazoline-2-ylidene] was utilized to catalyze the Mizoroki–Heck cross-coupling reaction of aryl bromides and iodides with styrene in water. According to the authors, this is the first report of a Pd-PEPPSI-type catalyst successfully employed in the aqueous-phase Mizoroki–Heck reaction. Good-to-excellent yields of the coupled products were obtained for a range of aryl bromides and iodides at 100 °C and with 1 mol % catalyst loading.^276^

### 5.2 Micellar Technology

**Figure 14:**
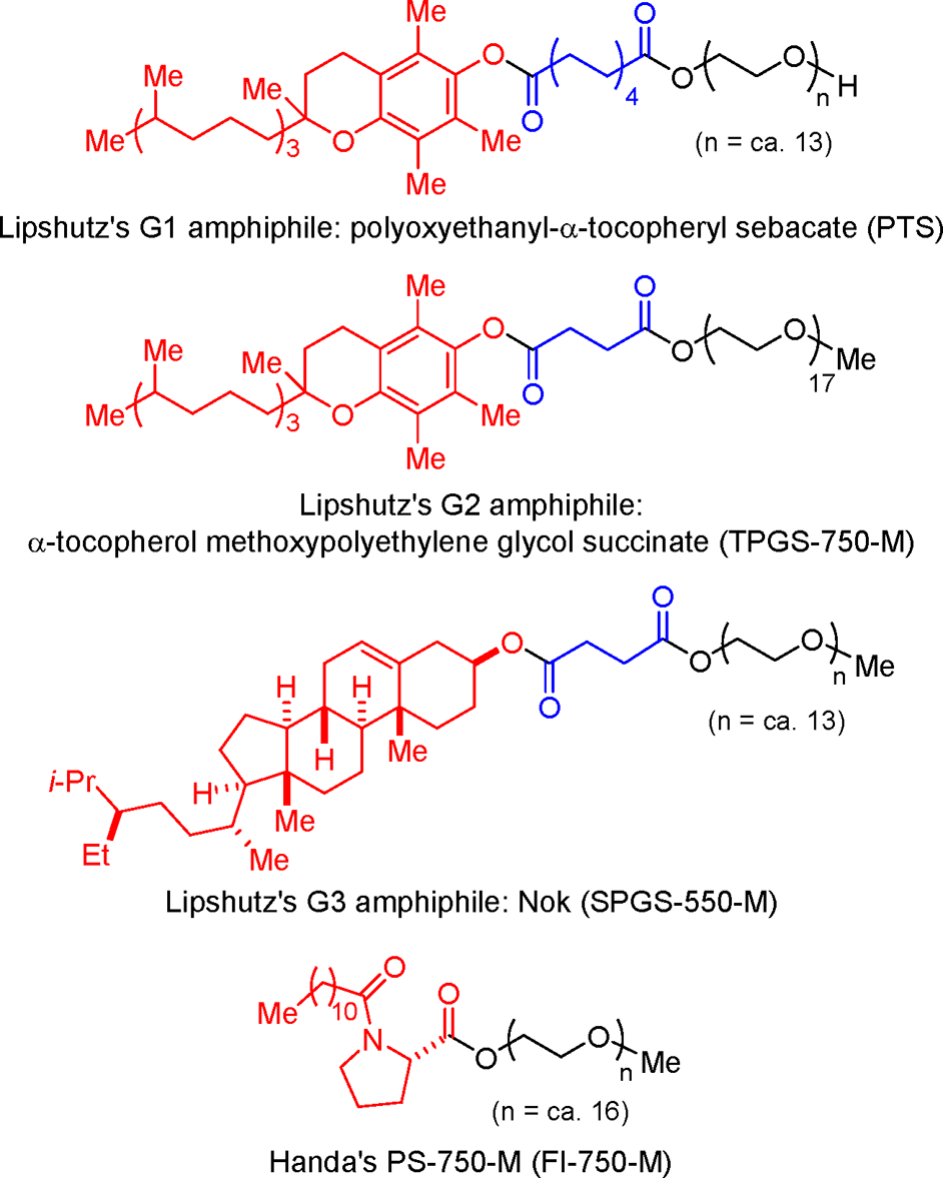
Popular surfactants developed for aqueous catalysis: (a) Lipshutz’s three generations of designer surfactants; (b) Handa’s PS-750-M surfactant.

As enumerated in Kobayashi’s review, there exist today several technologies for carrying out catalysis in water.^274^ Of these, surfactants have been an important part of a simple way to solubilize hydrophobic substrates in water by forming emulsions. The dissolution of the catalyst and reagents takes place in nanosized apolar aggregates formed by the surfactant in the aqueous medium via intermolecular interactions such as ion pairing and hydrophobic effects. These interactions mimic somewhat the biosynthesis that takes place in nature through enzymatic action in the aqueous medium. Consequently, the design and development of novel surfactants have been taking place in earnest for the purpose of engineering micelles that can promote organic synthesis in a way that competes favorably with traditional catalysis in organic solvents (Figure 14).^277−279^

#### 5.2.1 Application of Palladacycles in Cross-Couplings in Aqueous Media

**Scheme 55:**
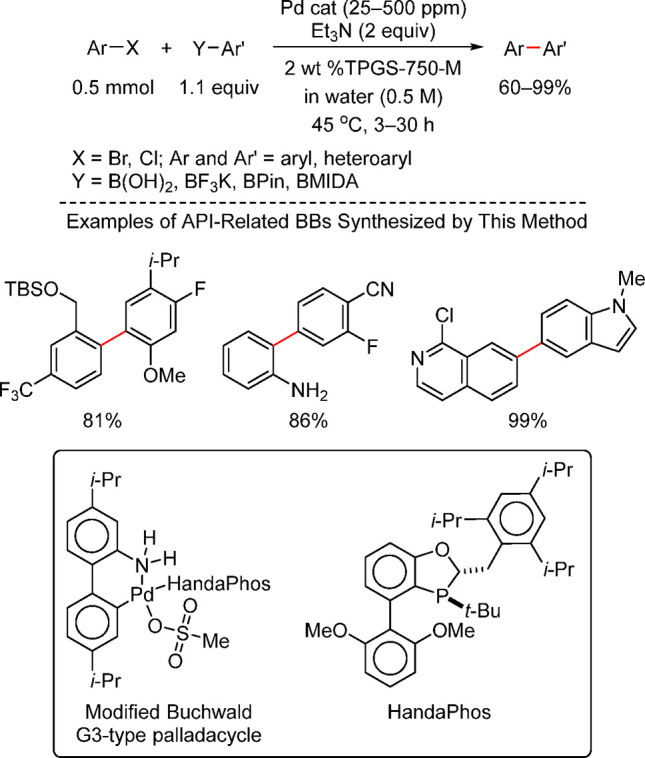
Lipshutz’s Suzuki–Miyaura Coupling in Aqueous Micellar Medium

Modifying the core of the Buchwald G3 palladacycle by introducing isopropyl substituents into the biphenyl moiety of the palladacycle and utilizing HandaPhos^280^ as ligand, Lipshutz and co-workers performed Suzuki–Miyaura cross-couplings in aqueous micellar media with a commercially available designer surfactant, TPGS-750 M, under mild conditions and with catalyst loadings most often as low as 300 ppm (0.03 mol %) (Scheme 55).^281^ This in stark contrast to the traditional Suzuki–Miyaura cross-coupling in organic solvents, which typically requires heating of the reaction mixture and catalyst loadings in the 5000 to 20 000 ppm range. The micellar Suzuki–Miyaura coupling can also be run on a multigram scale, and the aqueous reaction medium can be recycled and reused as often as four times; however, unlike in the case of the aforementioned water-soluble catalyst systems, the catalyst here could not be recycled.

In an aqueous system containing the proposed “micellar nanoreactors”,^282,283^ solubilization of the precatalyst is a crucial parameter to consider. In this regard, the underpinning of efficient Suzuki–Miyaura couplings in water is the binding constant of a reagent to the micellar inner core: the greater the incentive to enter the site of reaction, the more catalytic activity is to be expected and the lower the catalyst loading. Thus, the two isopropyl groups on the biphenyl moiety of the palladium complex make it more lipophilic, which leads to better activity and efficiency in the Suzuki–Miyaura cross-coupling (see Scheme 55). The low loading and the recycling of the aqueous reaction mixture, involving the same reactants or different coupling partners, were demonstrated several times. However, because the authors had to add catalyst in each recycling run, the recycling was done to reuse the surfactant and water only. The products were easily isolated by a very simple workup, and because no organic workup was needed, an E factor^284,285^ of zero was assumed based on the reaction conditions.^281^ Even when aqueous waste is factored in, the E factor remained considerably low at 1.7. Low-temperature microscopy (cryo-TEM) established the nature and size of the micellar particles acting as nanoreactors. ICP-MS analyses of residual palladium in the coupled products indicated very low levels of Pd that are within the U.S. FDA’s allowable exposure limits (<100 μg/day).^286−288^

**Scheme 56:**
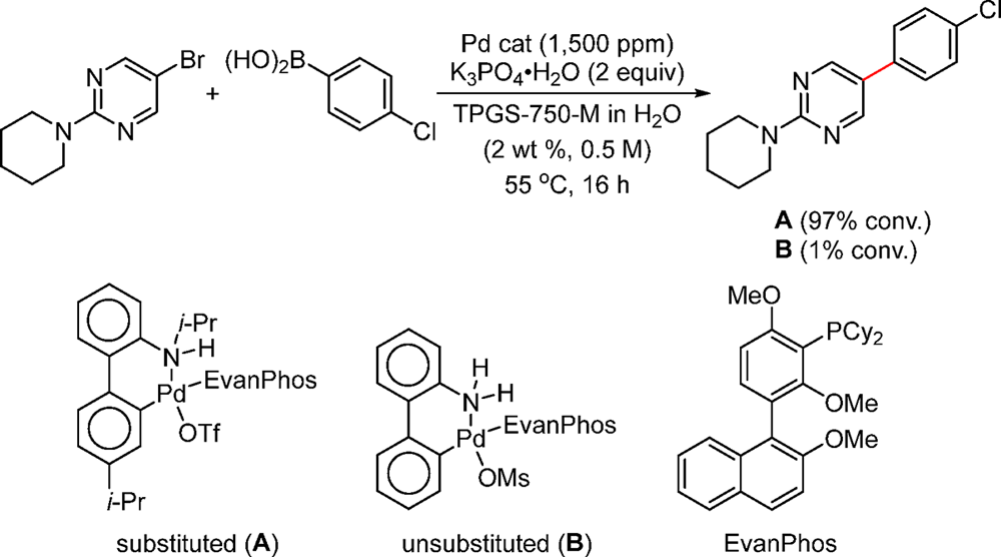
Performance of Substituted vs Unsubstituted EvanPhos Palladacyle in the Suzuki–Miyaura Coupling in Aqueous Micellar Medium

Lipshutz’s lab further modified the palladacycle precatalyst, whereby one N–H of the biphenyl amino group was replaced with *i*-Pr, while one of the *i*-Pr groups on the biphenyl moiety was removed, unlike in the previous system. Interestingly, the *N*-*i*-Pr substituted version of the precatalyst (**A**), with the new EvanPhos ligand that is relatively easy to make in comparison with the HandaPhos ligand,^289,290^ gave 97% conversion vs 1% conversion for the conventional unsubstituted palladacycle (**B**) (Scheme 56).^281,291^ However, the Pd loadings were a few orders of magnitude higher than those of the HandaPhos system described earlier.^281^ Surprisingly, the analogous EvanPhos–palladacycle complex, with an *i*-Pr group on each ring of the biphenyl moiety, gave much inferior results, presumably due to the mismatch of sterics to be able to fit into the “nanoreactors”.^291^ In addition, changing the isopropyl to a *t*-Bu group^291^ also gave inferior results.

#### 5.2.2 Application of L_1_Pd[π-(R-allyl)](X) Systems in Cross-Couplings in Aqueous Media

**Scheme 57:**
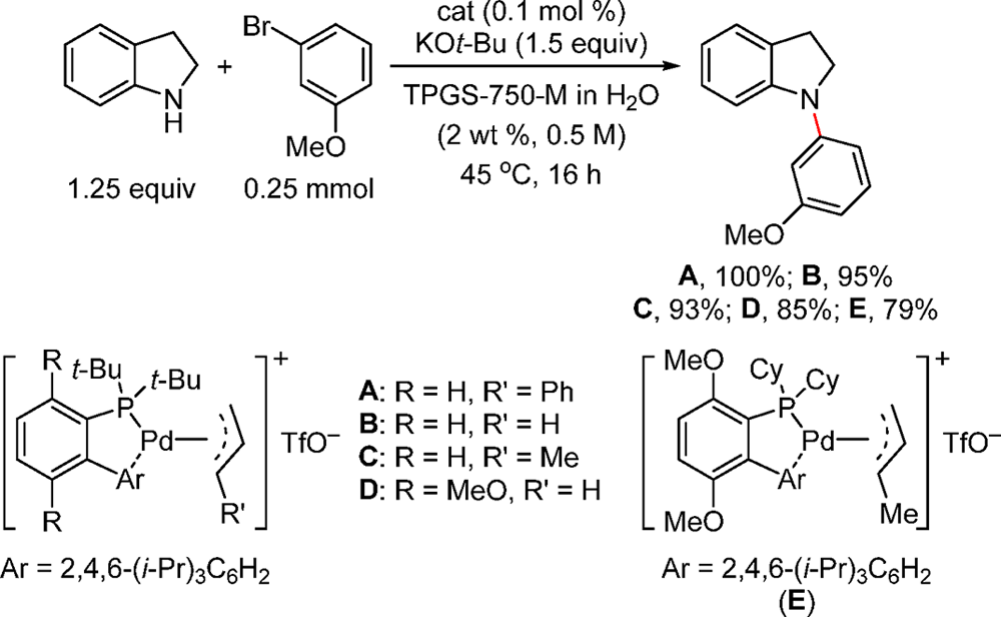
Cationic Biarylphosphine π-Allyl Pd Precatalysts Provide the Highest (NMR) Yields in the Difficult Indoline *N*-Arylation Reaction in Aqueous Medium

**Scheme 58:**
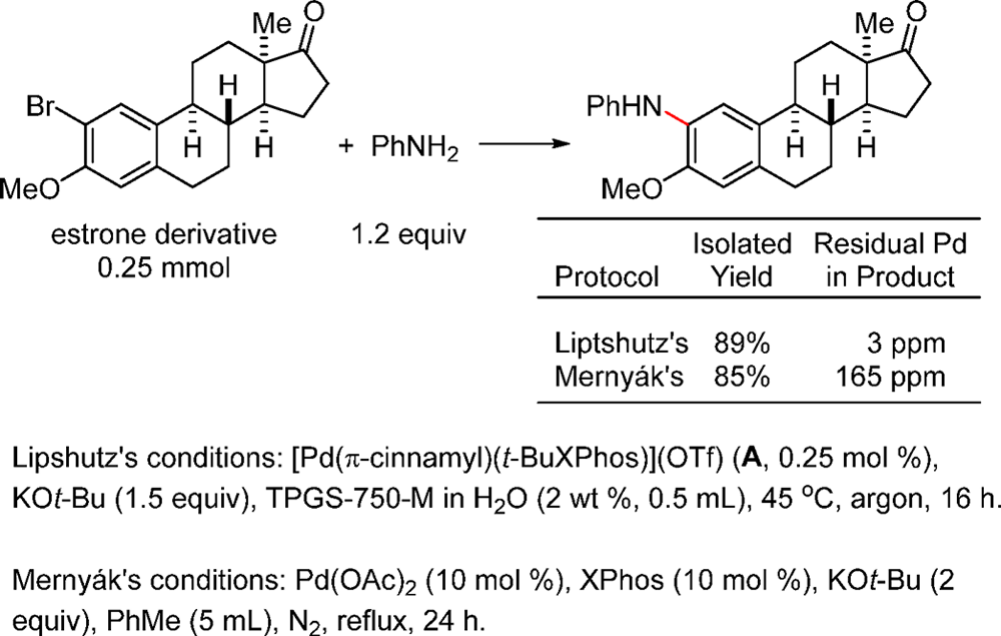
Example of the Application of Lipshutz’s Pd-Catalyzed Amination in Micellar Systems under Mild Aqueous Conditions to the Synthesis of Biologically Relevant Compounds

Surfactant-based micellar technology has also been very effective for conducting various cross-couplings in water using L_1_Pd[(π-(R-allyl)](X) precatalysts (R = H (allyl), Me (crotyl), Ph (cinnamyl); L = biaryl ligand; X= Cl, OTf) developed by Colacot and co-workers.^187^ Lipshutz and co-workers screened a variety of precatalysts, including mono- and biscoordinated Pd complexes, for the Buchwald–Hartwig N-arylation of indoline, a challenging model substrate, in aqueous medium. Among the various ligands tested, *t*-BuXPhos stood out as the best ligand, and the cationic (*t*-BuXphos)Pd(π-cinnamyl)(OTf) precatalyst led to a quantitative (NMR) yield of the C–N coupling product. Moreover, the cationic crotyl and allyl counterparts gave slightly lower (NMR) yields, 93% and 95%, respectively (Scheme 57).^292,293^ It is worth noting that the neutral Buchwald (*t*-BuXPhos)Pd G1 and G3 complexes also gave high yields. Interestingly, this protocol was applied by the same group to the efficient synthesis of a series of pharmaceutically important intermediates and derivatives, where Lipshutz’s aqueous conditions resulted in superior results vis-à-vis Mernyák’s classical conditions (Scheme 58).^292,293^

**Scheme 59:**
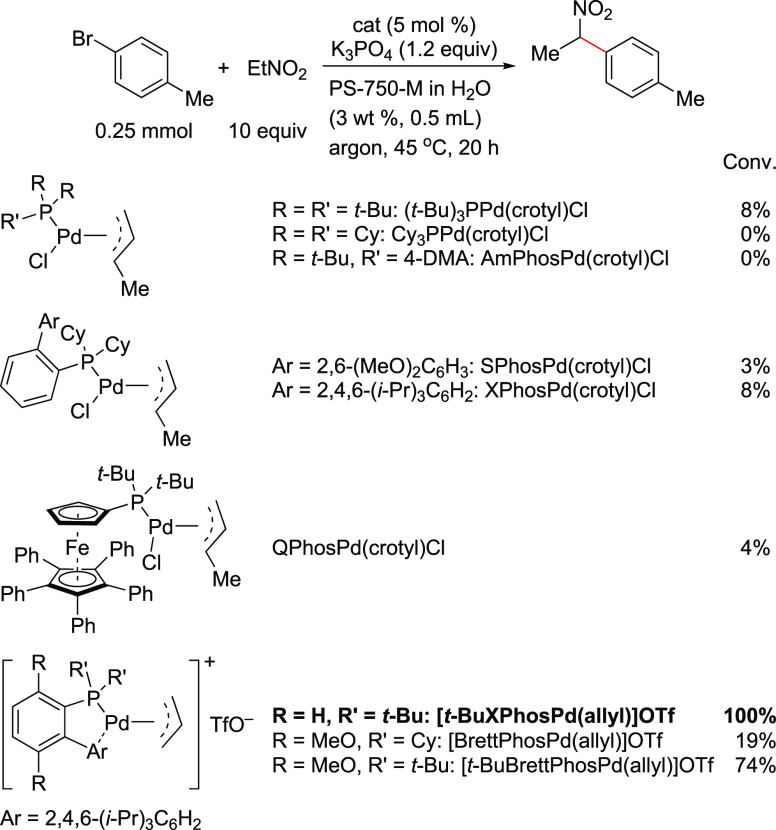
Handa’s Evaluation of the Performance of Various Precatalysts in the Cross-Coupling of Nitroalkanes with Aryl Bromides

**Scheme 60:**
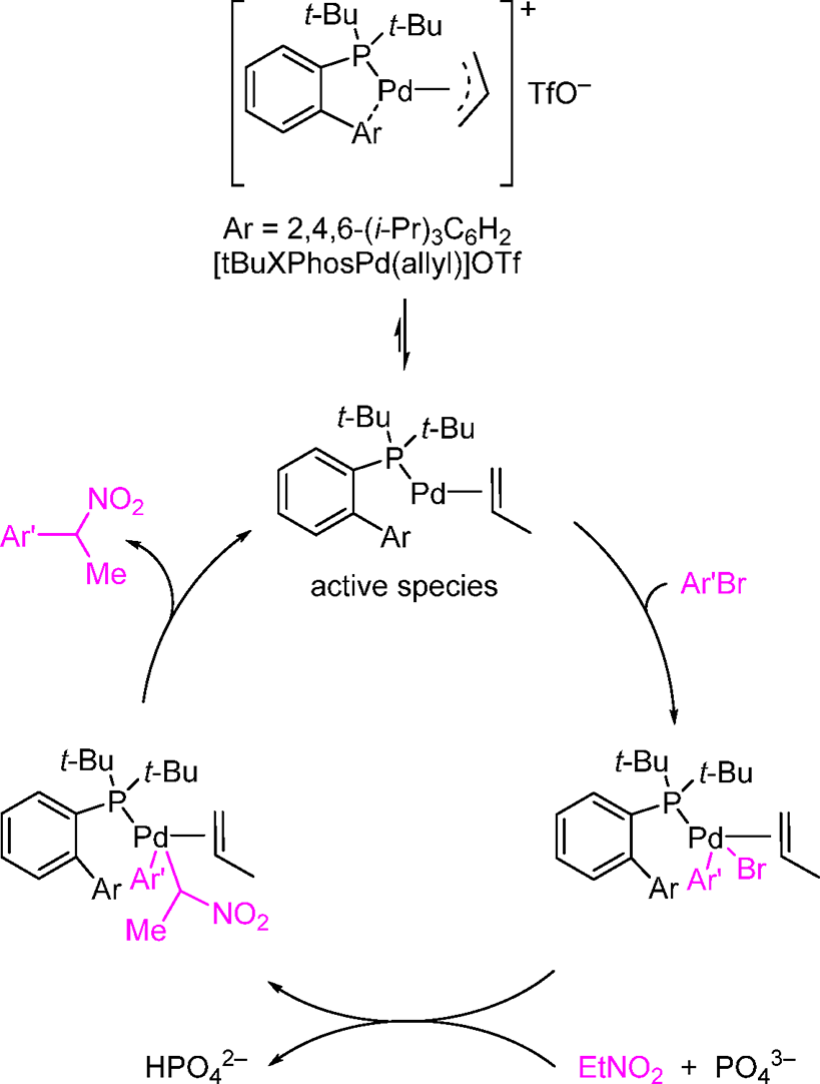
Handa’s Proposed Unconventional Mechanism for the Cross-Coupling of Nitroalkanes with Aryl Bromides

Handa and co-workers have also achieved the sp^2^–sp^3^ coupling of nitroalkanes with aryl bromides in water by utilizing L(π-allyl)PdOTf^187^ in conjunction with surfactant PS-750-M (FI-750-M) to mimic polar solvents such as DMF and 1,4-dioxane (Scheme 59).^277−279,294^ This method proved superior to the conventional technology that employs the Pd_2_dba_3_/XPhos system with 10 mol % Pd loading under glovebox conditions in anhydrous 1,4-dioxane at 80 °C.^277−279^ Following screening of various types of Pd sources while keeping *t*-BuXPhos as the ligand, the in situ catalysis employing Pd_2_dba_3_ or Pd(OAc)_2_ as the Pd source resulted in inferior yields even at higher Pd loadings in comparison to the Pd(π-allyl)(*t*-BuXphos)OTf system. In contrast to Lipshutz’s observations that substituents on the allyl group improve the activity and efficiency of the catalyst,^292,293^ the presence of an unsubstituted allyl group seems to be important in Handa’s results. However, Handa’s team did not screen the corresponding crotyl or cinnamyl Pd complexes with OTf as the counterion. Compared to chloride, the OTf counterion typically imparts a cationic character to the Pd complex. It is worth noting that when the same workers added propylene and allyl bromide to the in situ system containing Pd(OAc)_2_ and *t*-BuXPhos, comparable conversion was observed, albeit with a higher Pd loading. In the proposed mechanism (Scheme 60),^294^ it is postulated that the allyl group remains attached to the Pd throughout the catalytic cycle. However, detailed mechanistic studies are still needed to support this hypothesis, because, in such transformations, the typical oxidative addition takes place on L_*n*_Pd(0). Slow conversions were observed when these workers tried full recycling of the catalyst. However, the greenness of the process was demonstrated when they carried out full reaction medium recycling together with partial Pd recycling, resulting in a low E factor of 5.3 (when the solvent used in the chromatographic separation was recovered) or of 18.4 (chromatography solvent not recovered in the last cycle). Based on the results from Handa’s and Lipshutz’s studies, it would appear that the catalyst is getting inactivated after each reaction cycle. This is not surprising as the L_1_Pd(0) species is highly reactive and hence susceptible to degradation.

Despite the significant advances achieved in understanding and applying cross-coupling chemistry, the sustainable cross-coupling of quinoline and isoquinoline had remained underdeveloped.^295−297^ Using L_1_Pd(crotyl)X (X = Cl, OTf) based systems, Handa et al. recently reported a protocol for carrying out sustainable and clean Suzuki–Miyaura couplings of unactivated bromoquinolines and isoquinolines with a wide variety of coupling partners in water by employing PS-750-M (FI-750-M) as surfactant.^298^ In this type of chemistry, the size of the ligand seems to be important. The best result (100% conversion) was obtained with the medium sized PCy_3_ ligand in Cy_3_PPd(crotyl)Cl, while a good conversion (84%) was observed with the bulkier (*t*-Bu)_3_P based catalyst, (*t*-Bu)_3_PPd(crotyl)Cl. Among the Buchwald ligands, SPhos, XPhos, and RuPhos gave 90–94% conversions, while the bulky *t*-BuBrettPhos gave only a trace amount of the cross-coupling biaryl product.^298^

**Scheme 61:**
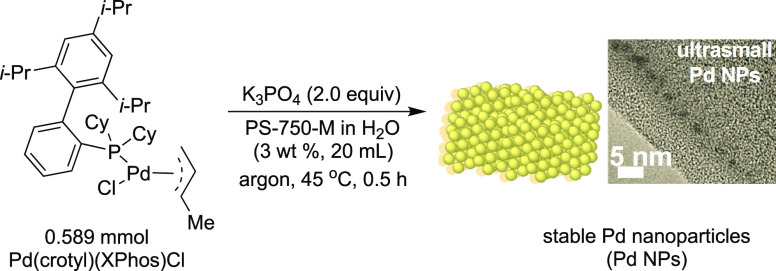
Formation of Palladium Nanoparticles from XPhosPd(crotyl)Cl under Micellar Conditions Adapted with permission from ref (299). Copyright 2020 American Chemical Society.

One of the unprecedented findings in this area is the formation of ultrasmall palladium nanoparticles (Pd NPs) under micellar conditions from the precatalyst XPhosPd(crotyl)Cl (Scheme 61).^299^ The authors noted that only π-allyl complexes^187^ formed Pd NPs, whereas other phosphine-PdCl_2_ or phosphine-Pd(OAc)_2_ complexes proved ineffective in this regard. The presence of the crotyl group in the precatalyst is key to the fast reductive elimination of crotyl chloride, resulting in formation of the Pd NPs both in the small (1 g) and large (20 g) scale runs.

The composition, ligation, morphology, and size distribution of the Pd NPs were determined by employing analytical techniques such as ^31^P NMR spectroscopy, high-resolution transmission electron microscopy (HRTEM), scanning transmission electron-microscopy-based high-angle annular dark-field imaging (STEM-HAADF), and energy-dispersive X-ray spectroscopy (EDX) mapping. Very interestingly, the XPhos-complexed ultrasmall Pd NPs showed a single peak at 43.1 ppm (confirming the binding of the phosphine ligand) vs −12.9 ppm for the free XPhos; Ph_3_P in a sealed capillary served as the internal standard (−6 ppm).

**Scheme 62:**
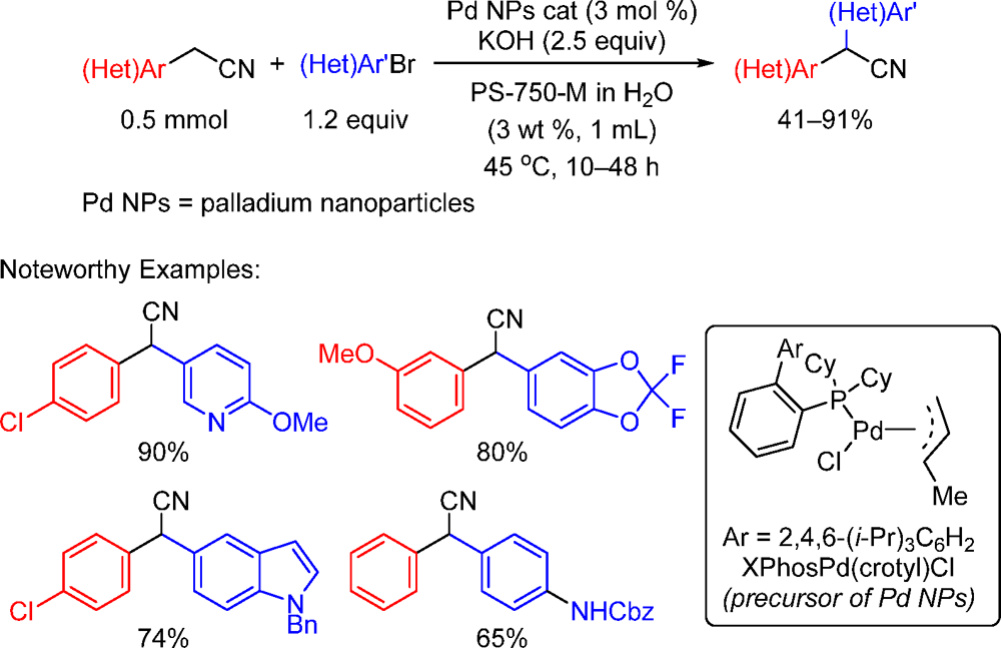
Leahy and Handa’s α-Arylation Reaction of Nitriles in Aqueous Micellar Medium Facilitated by Ultrasmall Palladium Nanoparticles

The Pd NPs formed from XPhosPd(crotyl)Cl catalyzed the α-arylation of nitriles in aqueous micellar medium using PS-750-M as surfactant. There is evidence that Pd-bound carbanions or keteniminates are formed as intermediates, which are stabilized inside the hydrophobic core of the micellar environment and thus protected from quenching (protonation) by water. The generality of the reaction was established with about 35 examples, including one on a 50 g scale (Scheme 62).^299^ The details of the cross-coupling reaction pathway, such as oxidative addition to Pd NPs, transmetalation, and reductive elimination, were established with control ^31^P NMR experiments on a stoichiometric variant.

**Scheme 63:**
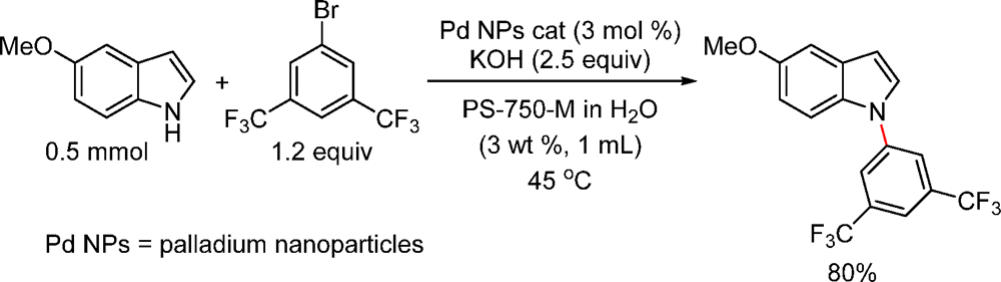
Proof of Concept of the Suitability of the Ultrasmall Pd NPs Catalytic System for the Buchwald–Hartwig Amination of Indoles with Aryl Bromides

The broader reactivity and applicability of the Pd NPs catalyst system and micellar conditions were demonstrated in the synthesis of biaryl ketones in 65–78% yields by a one-pot α-arylation of nitriles with heteroaryl bromides followed by oxidation with elemental oxygen. More importantly, in a proof-of-concept experiment, the protocol was shown to be effective for the Buchwald–Hartwig amination of indoles with aryl bromides (Scheme 63).^299^

## 6 Selected Industrial Applications of L_1_Pd(0) Precatalysts

Several industrial applications of the methods and protocols described in the preceding sections have been reported. In Table 1, we feature selected pharmaceutically relevant molecules synthesized by utilizing these catalyst systems in a variety of coupling reactions such as the Suzuki–Miyaura, Mizoroki–Heck, Buchwald–Hartwig, and Negishi couplings, as well as the Miyaura borylation, allylation, and α-arylation reactions. The examples were chosen from the patent literature and journal articles published by industrial research groups. Therefore, because of the need to ensure a broad protection of the relevant intellectual property, the complete advantages of the novel 12-electron L_1_Pd(0) catalysts over conventional ones may not have been fully disclosed. Nevertheless, we discuss in detail the synthesis of a few drug molecules, wherein the advantages of the 12-electron L_1_Pd(0) precatalysts over conventional catalysts are apparent.

**Table 1:**
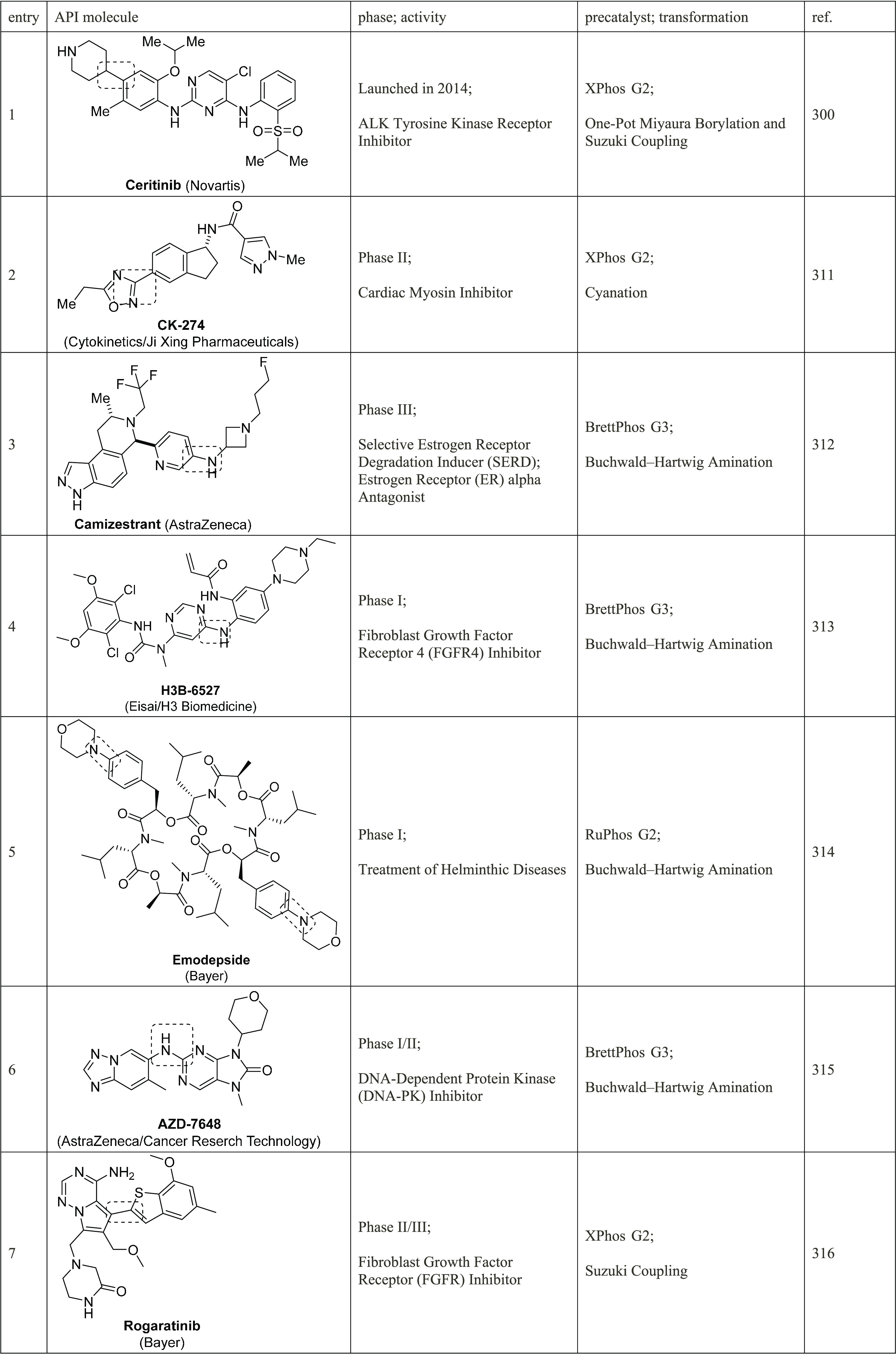

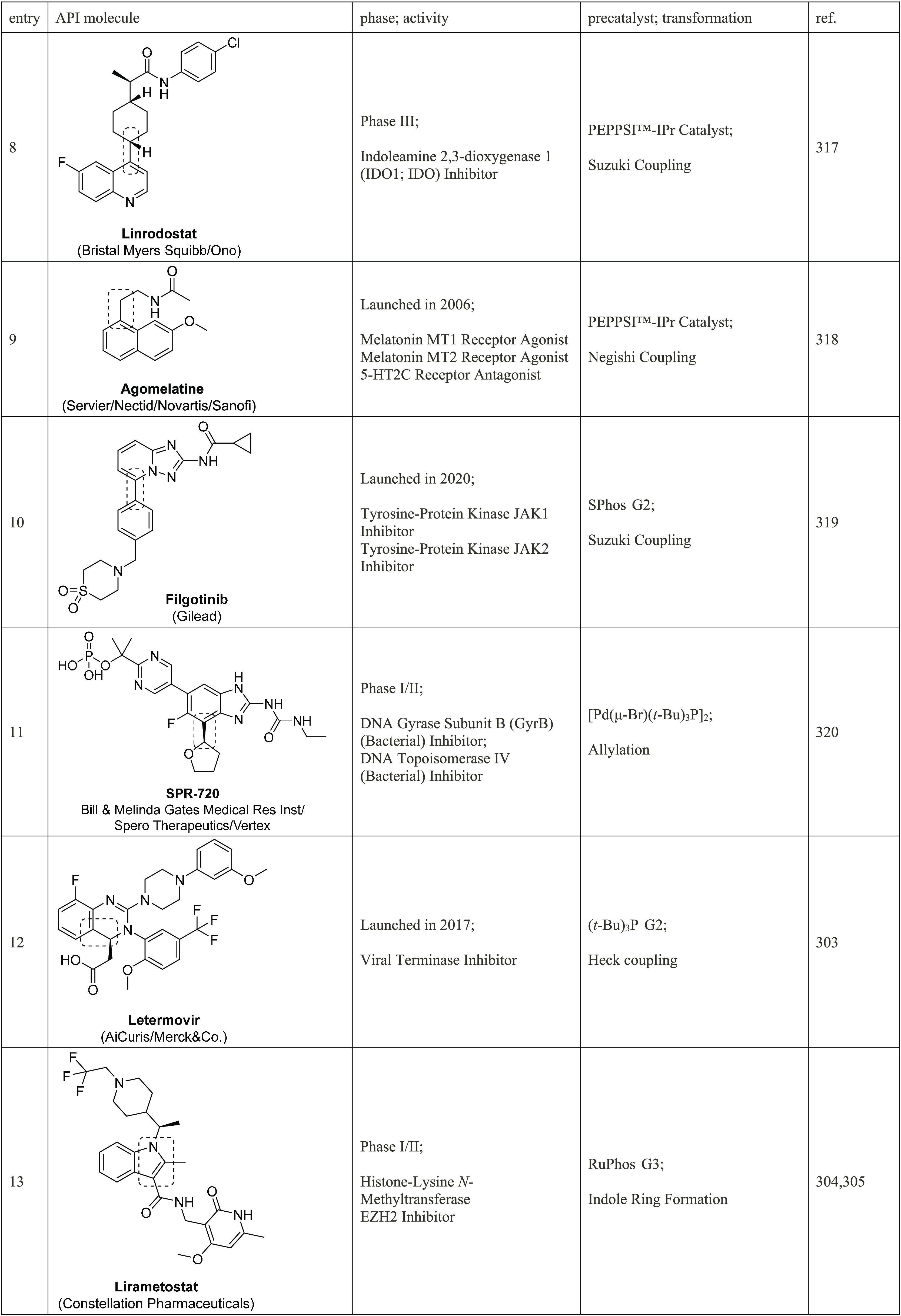

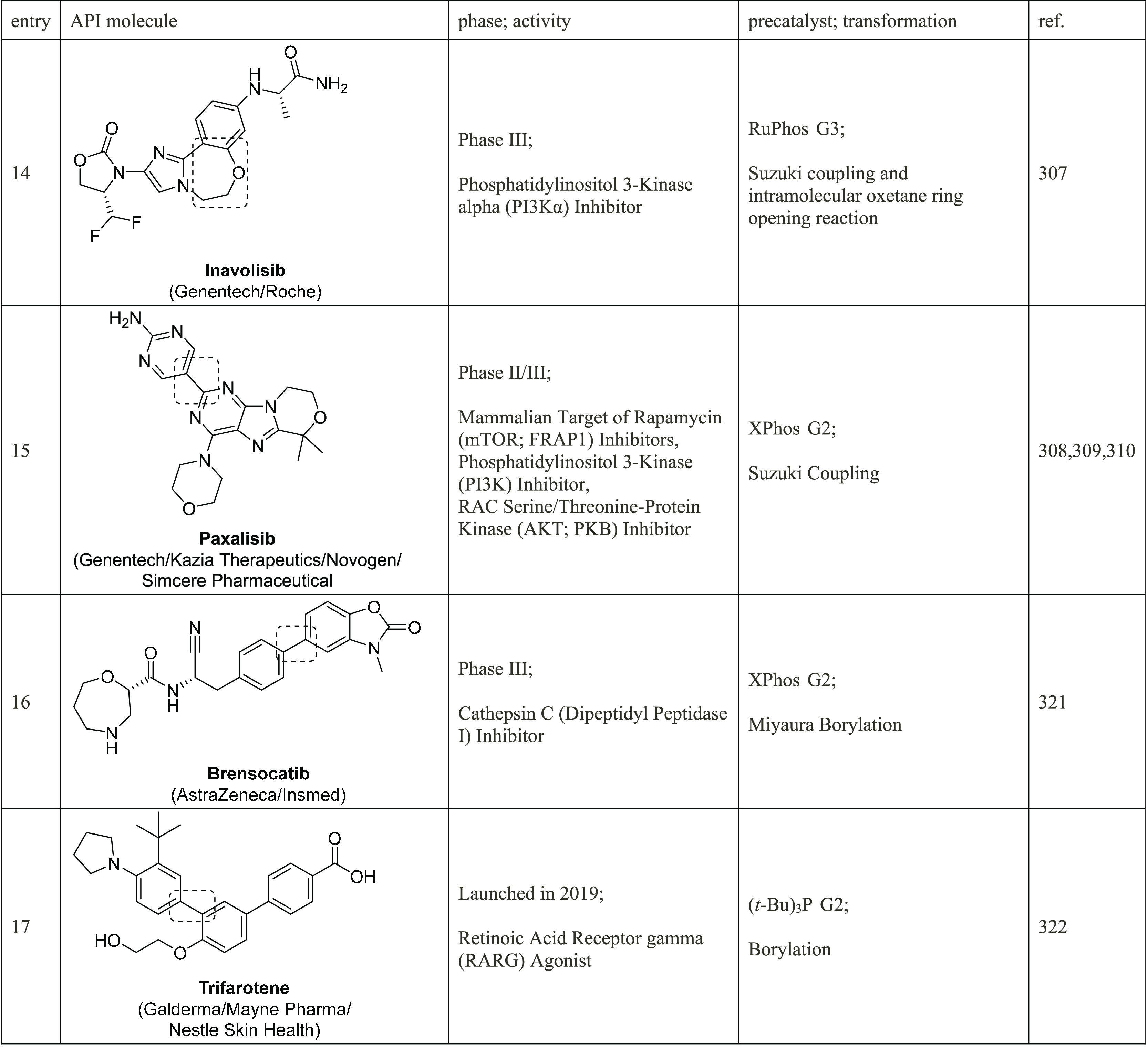
Pharmaceutically Relevant Molecules Synthesized by Utilizing 12-Electron-Based L_1_Pd(0) Precatalysts

**Scheme 64:**
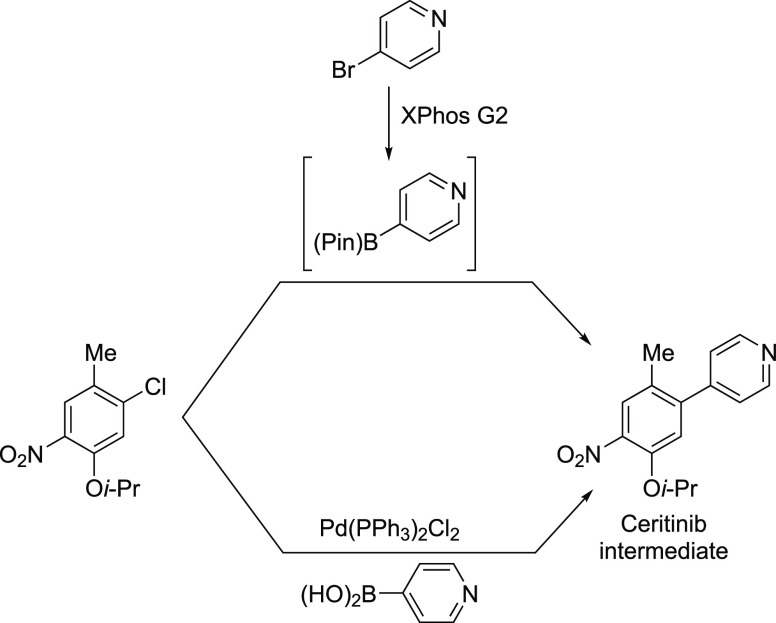
Application of L_1_Pd(0) Precatalyst in the Synthesis of Ceritinib Intermediate

For Ceritinib (Table 1, entry 1), the use of XPhos G2 helped carry out a one-pot synthesis involving both the Miyaura borylation and Suzuki coupling (Scheme 64).^300^ The conventional Pd(PPh_3_)_2_Cl_2_ catalyst required starting with a boronic acid that is available only in a 90% purity from commercial sources with varying amounts of anhydride impurity.^301^

**Scheme 65:**
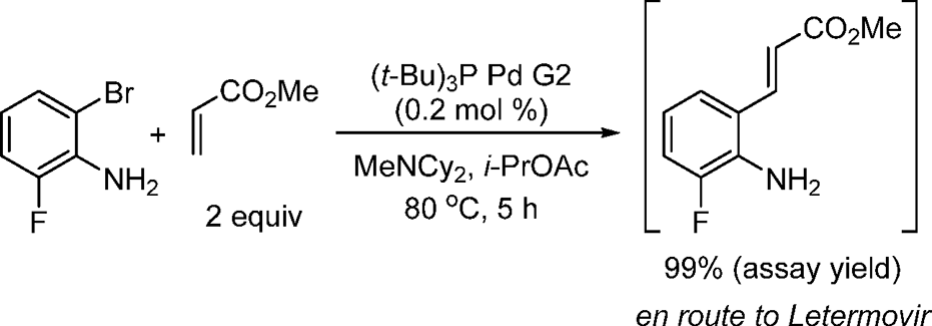
Application of L_1_Pd(0) Precatalyst in the Heck Coupling Step in a Relatively Short Asymmetric Synthesis of Letermovir

In the Heck coupling step en route to Letermovir (Table 1, entry 12), instead of the Pd(OAc)_2_/P(*o*-Tol)_3_ catalyst system^302^ with a possible loading of 9 mol %, only 0.2 mol % of (*t*-Bu)_3_P G2 was needed with significant improvement in reaction time, i.e., 5 h vs 48 h (Scheme 65).^303^

**Scheme 66:**
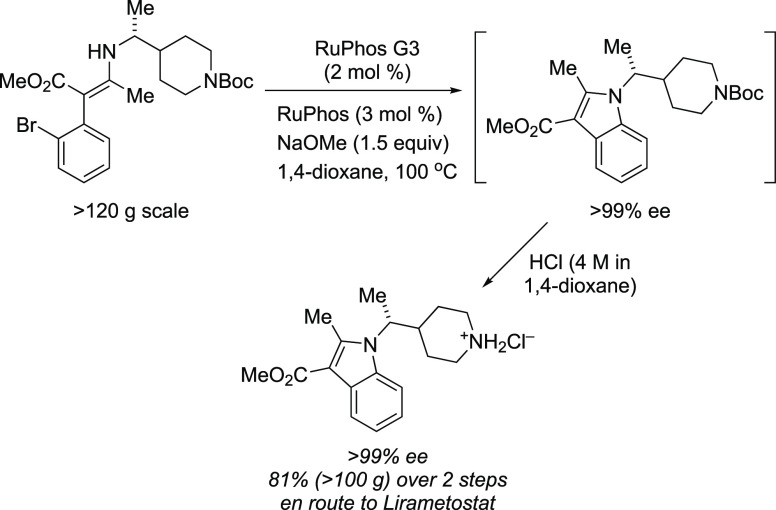
Application of L_1_Pd(0) Precatalyst in the Intramolecular C–N Arylation Step en Route to Lirametostat

Both preformed and in situ formed 12-electron catalysts have been effective in the indole ring formation for the synthesis of the API Lirametostat (Table 1, entry 13) (Scheme 66).^304,305^ This step employed RuPhos G3, which is an air-stable precatalyst in comparison to the one from the pyrophoric P(*t*-Bu)_3_.^306^

**Scheme 67:**
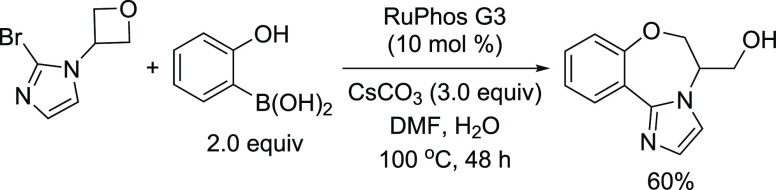
Use of L_1_Pd(0) Precatalyst to Form the Core Ring System of Inavolisib, a PI3kα Inhibitor

A one-pot tandem Suzuki/oxetane ring-opening/cyclization has been employed to form the eight-membered ring in the synthesis of Inavolisib (Table 1, entry 14) (Scheme 67).^307^

**Scheme 68:**
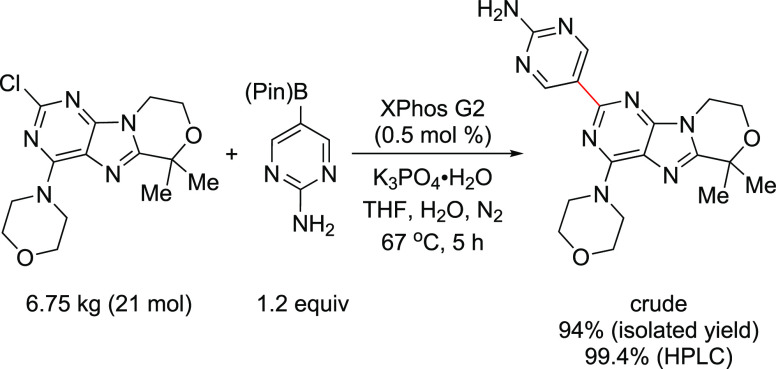
Last Step in an Efficient and Benign kg Scale Synthesis of Paxalisib, a Selective and Potent Inhibitor of PI3k and mTOR

In the synthesis of Paxalisib, replacing (dppf)PdCl_2_·CH_2_Cl_2_ with XPhos G2 facilitated the Suzuki–Miyaura coupling with a lower loading of 0.5 mol % compared with the 2 mol % loading needed with the first-generation catalyst (Table 1, entry 15) (Scheme 68).^308−310^

## 7 Summary and Outlook

Although about 4–5 Nobel Prizes in chemistry have been awarded to homogeneous catalysis from 2001 to 2021, the 2010 Nobel Prize winning technology, namely the Pd-catalyzed cross-coupling for carbon–carbon bond-forming reactions is the most practiced reaction in both academia and industry. This technology is also an example of how incremental innovation, with contributions from many pioneers, has led to a Nobel Prize. Moreover, the Murahashi reaction has recently been resurrected as the Murahashi–Feringa coupling with the significant contributions of Nobel Laureate Feringa.^311,323^ The C–N cross-coupling has also become very popular and industrially relevant; hence, we are anticipating another Nobel Prize in cross-coupling in the near future. These innovations in all the areas of cross-coupling are due to the continued development of new ligands and catalysts.^101^

Over the past decade, several new precatalyst technologies, such as Buchwald’s palladacycle technology, Colacot’s work on allyl/crotyl/cinnamyl based cationic complexes, and Buchwald’s OACs (G6), have been developed to create precursors for L_1_Pd(0) catalysts containing phosphine ligands as outlined in this review. Additionally, technologies employing NHCs for generating L_1_Pd(0) catalysts have been developed by Organ, Nolan, Szostak, and Hazari. The choice of ligand for the construction of the desired precatalyst is crucial for achieving better selectivity, reduced catalyst loading, and milder reaction conditions, all important considerations for successfully and sustainably carrying out a challenging reaction for real world applications. The facile activation of the precatalyst to L_1_Pd(0) varies from one system to another and, therefore, the ease of synthesis of the ligands and precatalysts is also important.

With the aid of mechanistic and kinetic studies, our understanding of the cross-coupling mechanism has evolved somewhat, whereby L_1_Pd(0) seems to be the active species in the catalytic cycle for all three major steps (oxidative addition, transmetalation, and reductive elimination), even with smaller ligands such as Ph_3_P. However, sterically demanding ligands help to form L_1_Pd(0) more easily than the less bulky ligands such as Ph_3_P. Having said that, the stability of the L*_n_*Pd(0) species is important for higher TONs, as L_1_Pd(0) may get deactivated depending on the reaction conditions and the type of ligand used. Based on Hirschi and Vetticatt’s recent studies combining DFT calculations and experimental ^13^C kinetic isotope effects, the proposed catalytic cycle even for Ph_3_P-based systems is shown in Scheme 5 (cf. section 2.1), where L_1_Pd(0) is the active catalytic species after the first cycle in the absence of excess Ph_3_P.^56^

AI based computational and machine learning technologies have emerged as powerful tools in predicting the active ligand/catalyst system by making use of ligand parametrization. In this context, Gensch, Sigman, Aspuru-Guzik, and co-workers very recently presented “*kraken”*, a discovery platform for monodentate organophosphorus(III) ligands that provides comprehensive physicochemical descriptors on the basis of representative conformer sets. By utilizing quantum-mechanical methods, they were able to calculate descriptors for over 1500 ligands, including some representative commercially available ones, and used machine learning simulations to predict properties of over 300 000 new ligands.^121,123^ Our company (MilliporeSigma, business of Merck KGaA, Darmstadt, Germany) is expanding the scope of this AI-based digital technology for commercialization to help industrial and academic customers to accelerate their research. Related predictive studies by Sigman, Doyle, and Schoenebeck, already discussed in section 3, clearly show that newer parameters beyond Toleman’s cone angle can be utilized to predict the formation of monoligated Pd complexes generating 12-electron-based catalytic systems. However, to be able to make AI-based, foolproof predictions, there is still a need to incorporate substrate parametrization, reaction conditions, and clean and reliable data, in addition to ligand and catalyst parametrization. Nevertheless, these methods have the potential to be employed for industrial applications with a focus on identifying suitable precatalysts, thereby minimizing Pd loadings, reducing harsh reaction conditions, and increasing reaction output in terms of yield and selectivity. MilliporeSigma (Merck KGaA, Darmstadt, Germany) is currently working on commercializing a digital tool based on AI technology which will support both academic and industrial customers to optimize coupling reactions, with an emphasis on medicinal and process chemistry.

With the increase in palladium metal prices in recent years, the cost-effective high yield synthesis of new-generation monoligated precatalysts is crucial for the cross-coupling technology to grow further. One way this could be achieved is by minimizing the number of steps in manufacturing commercial catalysts, which in turn minimizes Pd losses during the process. Also developing technologies for low loading Pd catalytic processes as well as efficient and cost-effective Pd capture and recovery methods need to be developed to efficiently recycle the expensive palladium metal. In addition, alternate technologies involving earth abundant metals, photocatalytic and electrochemical methodologies and enzymatic methodologies need to be developed from a sustainability point of view.

## References

[ref1] The Nobel Prize in Chemistry 2010. The Nobel Prize; Nobel Prize Outreach AB, 2022; https://www.nobelprize.org/prizes/chemistry/2010/summary/ (accessed 2021-08-26).

[ref2] Johansson-Seechurn C. C. C.; Kitching M. O.; Colacot T. J.; Snieckus V. Palladium-Catalyzed Cross-Coupling: A Historical Contextual Perspective to the 2010 Nobel Prize. Angew. Chem., Int. Ed. 2012, 51, 5062–5085. 10.1002/anie.201107017. 22573393

[ref3] Negishi E.; de Meijere A., Eds. Handbook of Organopalladium Chemistry for Organic Synthesis; Wiley: New York, 2002; Volumes 1 and 2.

[ref4] Negishi E. Selective Carbon–Carbon Bond Formation via Transition Metal Catalysis: Is Nickel or Palladium Better than Copper? In Aspects of Mechanism and Organometallic Chemistry; Brewster J. H., Ed.; Springer, 1978; pp 285–317.

[ref5] Littke A. F.; Fu G. C. A Convenient and General Method for Pd-Catalyzed Suzuki Cross-Couplings of Aryl Chlorides and Arylboronic Acids. Angew. Chem., Int. Ed. 1998, 37, 3387–3388. 10.1002/(SICI)1521-3773(19981231)37:24<3387::AID-ANIE3387>3.0.CO;2-P. 29711304

[ref6] For the first review of Pd-catalyzed coupling of aryl chlorides, see Littke A. F.; Fu G. C. Palladium-Catalyzed Coupling Reactions of Aryl Chlorides. Angew. Chem., Int. Ed. 2002, 41, 4176–4211. 10.1002/1521-3773(20021115)41:22<4176::AID-ANIE4176>3.0.CO;2-U. 12434342

[ref7] Nishiyama M.; Yamamoto T.; Koie Y. Synthesis of N-Arylpiperazines from Aryl Halides and Piperazine under a Palladium Tri-*tert*-butylphosphine Catalyst. Tetrahedron Lett. 1998, 39, 617–620. 10.1016/S0040-4039(97)10659-1.

[ref8] Yamamoto T.; Nishiyama M.; Koie Y. Palladium-Catalyzed Synthesis of Triarylamines from Aryl Halides and Diarylamines. Tetrahedron Lett. 1998, 39, 2367–2370. 10.1016/S0040-4039(98)00202-0.

[ref9] Old D. W.; Wolfe J. P.; Buchwald S. L. A Highly Active Catalyst for Palladium-Catalyzed Cross-Coupling Reactions: Room-Temperature Suzuki Couplings and Amination of Unactivated Aryl Chlorides. J. Am. Chem. Soc. 1998, 120, 9722–9723. 10.1021/ja982250+.

[ref10] Hamann B. C.; Hartwig J. F. Sterically Hindered Chelating Alkyl Phosphines Provide Large Rate Accelerations in Palladium-Catalyzed Amination of Aryl Iodides, Bromides, and Chlorides, and the First Amination of Aryl Tosylates. J. Am. Chem. Soc. 1998, 120, 7369–7370. 10.1021/ja981318i.

[ref11] DeAngelis A.; Colacot T. J. Prominent Ligand Types in Modern Cross-Coupling Reactions. In New Trends in Cross-Coupling: Theory and Applications; RSC Catalysis Series No. 21; Colacot T. J., Hardcare C., Ismagilov Z., Ozkan U., Eds.; Royal Society of Chemistry: Cambridge, U.K., 2015; Chapter 2, pp 20–90, 10.1039/9781782620259-00020.

[ref12] Arduengo A. J. III; Harlow R. L.; Kline M. A Stable Crystalline Carbene. J. Am. Chem. Soc. 1991, 113, 361–363. 10.1021/ja00001a054.

[ref13] Herrmann W. A.; Brossmer C.; Öfele K.; Beller M.; Fischer H. Zum Mechanismus der Heck-Reaktion: Katalysator-Deaktivierung durch PC-Bindungsbruch. J. Organomet. Chem. 1995, 491, C1–C4. 10.1016/0022-328X(94)05318-6.

[ref14] Herrmann W. A.; Elison M.; Fischer J.; Köcher C.; Artus G. R. J. Metal Complexes of N-Heterocyclic Carbenes–A New Structural Principle for Catalysts in Homogeneous Catalysis. Angew. Chem., Int. Ed. 1995, 34, 2371–2374. 10.1002/anie.199523711.

[ref15] Viciu M. S.; Germaneau R. F.; Navarro-Fernandez O.; Stevens E. D.; Nolan S. P. Activation and Reactivity of (NHC)Pd(allyl)Cl (NHC = N-Heterocyclic Carbene) Complexes in Cross-Coupling Reactions. Organometallics 2002, 21, 5470–5472. 10.1021/om020804i.

[ref16] For a recent book chapter, see Chartoire A.; Nolan S. P. Advances in C–C and C–X Coupling Using Palladium–N-Heterocyclic Carbene (Pd–NHC) Complexes. In New Trends in Cross-Coupling:Theory and Applications; RSC Catalysis Series No. 21; Colacot T. J., Hardcare C., Ismagilov Z., Ozkan U., Eds.; Royal Society of Chemistry: Cambridge, UK, 2015; Chapter 4, pp 139–227, 10.1039/9781782620259-00139.

[ref17] For an original review, see Marion N.; Nolan S. P. Well-Defined N-Heterocyclic Carbenes–Palladium(II) Precatalysts for Cross-Coupling Reactions. Acc. Chem. Res. 2008, 41, 1440–1449. 10.1021/ar800020y. 18774825

[ref18] Diez-González S.; Marion N.; Nolan S. P. N-Heterocyclic Carbenes in Late Transition Metal Catalysis. Chem. Rev. 2009, 109, 3612–3676. 10.1021/cr900074m. 19588961

[ref19] Clavier H.; Nolan S. P. Percent Buried Volume for Phosphine and N-Heterocyclic Carbene Ligands: Steric Properties in Organometallic Chemistry. Chem. Commun. 2010, 46, 841–861. 10.1039/b922984a. 20107630

[ref20] Clavier H.; Correa A.; Cavallo L.; Escudero-Adán E. C.; Benet-Buchholz J.; Slawin A. M. Z.; Nolan S. P. [Pd(NHC)(allyl)Cl] Complexes: Synthesis and Determination of the NHC Percent Buried Volume (%*V*_bur_) Steric Parameter. Eur. J. Inorg. Chem. 2009, 2009, 1767–1773. 10.1002/ejic.200801235.

[ref21] Kantchev E. A. B.; O’Brien C. J.; Organ M. G. Pd-N-Heterocyclic Carbene (NHC) Catalysts for Cross-Coupling Reactions. Aldrichimica Acta 2006, 39, 97–111.

[ref22] Kantchev E. A. B.; O’Brien C. J.; Organ M. G. Palladium Complexes of N-Heterocyclic Carbenes as Catalysts for Cross-Coupling Reactions—A Synthetic Chemist’s Perspective. Angew. Chem., Int. Ed. 2007, 46, 2768–2813. 10.1002/anie.200601663. 17410611

[ref23] Würtz S.; Glorius F. Surveying Sterically Demanding N-Heterocyclic Carbene Ligands with Restricted Flexibility for Palladium-Catalyzed Cross-Coupling Reactions. Acc. Chem. Res. 2008, 41, 1523–1533. 10.1021/ar8000876. 18720995

[ref24] Huser M.; Youinou M.-T.; Osborn J. A. Chlorocarbon Activation: Catalytic Carbonylation of Dichloromethane and Chlorobenzene. Angew. Chem., Int. Ed. Engl. 1989, 28, 1386–1388. 10.1002/anie.198913861.

[ref25] Ben-David Y.; Portnoy M.; Milstein D. Chelate-Assisted, Palladium-Catalyzed Efficient Carbonylation of Aryl Chlorides. J. Am. Chem. Soc. 1989, 111, 8742–8744. 10.1021/ja00205a039.

[ref26] Colacot T. J., Ed. New Trends in Cross-Coupling: Theory and Applications; RSC Catalysis Series, No. 21; Hardcare C., Colacot T., Ismagilov Z., Ozkan U., Eds.; Royal Society of Chemistry: Cambridge, UK, 2016; 10.1039/9781782620259-00091.

[ref27] Drahl C. Nano Nuisance for Palladium Source. Organometallic Chemistry: Catalyst Precursor’s Breakdown Complicates Efficiency Estimates.Chem. Eng. News 2012, 90 ( (March 13), ). https://cen.acs.org/articles/90/web/2012/03/Nano-Nuisance-Palladium-Source.html (accessed 2022-06-28).

[ref28] Ritter S. K. Chemists Introduce a User’s Guide for Palladium Acetate: Better Understanding of the Catalyst Precursor’s Properties Could Make Palladium Catalysis More Predictable and Reproducible. Chem. Eng. News 2016, 94, 20–21.

[ref29] Zalesskiy S. S.; Ananikov V. P. Pd_2_(dba)_3_ as a Precursor of Soluble Metal Complexes and Nanoparticles: Determination of Palladium Active Species for Catalysis and Synthesis. Organometallics 2012, 31, 2302–2309. 10.1021/om201217r.

[ref30] Carole W. A.; Bradley J.; Sarwar M.; Colacot T. J. Can Palladium Acetate Lose Its “Saltiness”? Catalytic Activities of the Impurities in Palladium Acetate. Org. Lett. 2015, 17, 5472–5475. 10.1021/acs.orglett.5b02835. 26507318

[ref31] Carole W. A.; Colacot T. J. Understanding Palladium Acetate from a User Perspective. Chem.—Eur. J. 2016, 22, 7686–7695. 10.1002/chem.201601450. 27125630

[ref32] Johansson Seechurn C. C. C.; Sperger T.; Scrase T. G.; Schoenebeck F.; Colacot T. J. Understanding the Unusual Reduction Mechanism of Pd(II) to Pd(I): Uncovering Hidden Species and Implications in Catalytic Cross-Coupling Reactions. J. Am. Chem. Soc. 2017, 139, 5194–5200. 10.1021/jacs.7b01110. 28300400

[ref33] Slack E. D.; Tancini P. D.; Colacot T. J. Process Economics and Atom Economy for Industrial Cross-Coupling Applications via L_n_Pd(0)-Based Catalysts. Top. Organomet. Chem. 2019, 65, 161–198. 10.1007/3418_2019_28.

[ref34] Li H.; Grasa G. A.; Colacot T. J. A Highly Efficient, Practical, and General Route for the Synthesis of (R_3_P)_2_Pd(0): Structural Evidence on the Reduction Mechanism of Pd(II) to Pd(0). Org. Lett. 2010, 12, 3332–3335. 10.1021/ol101106z. 20597473

[ref35] Langmuir I. Types of Valence. Science 1921, 54, 59–67. (b) 10.1126/science.54.1386.59. 17843674

[ref36] See also Jensen W. B. The Origin of the 18-Electron Rule. J. Chem. Educ. 2005, 82 (1), 28 10.1021/ed082p28.

[ref37] Miyaura N.; Suzuki A. Palladium-Catalyzed Cross-Coupling Reactions of Organoboron Compounds. Chem. Rev. 1995, 95, 2457–2483. 10.1021/cr00039a007.

[ref38] Farina V.; Krishnan B. Large Rate Accelerations in the Stille Reaction with Tri-2-furylphosphine and Triphenylarsine as Palladium Ligands: Mechanistic and Synthetic Implications. J. Am. Chem. Soc. 1991, 113, 9585–9595. 10.1021/ja00025a025.

[ref39] Johansson Seechurn C. C. C.; Li H.; Colacot T. J. Pd-Phosphine Precatalysts for Modern Cross-Coupling Reactions. In New Trends in Cross-Coupling: Theory and Applications; Colacot T. J., Hardcare C., Ismagilov Z., Ozkan U., Ed.; Royal Society of Chemistry: Cambridge, U.K., 2015; Chapter 3, pp 91–138, 10.1039/9781782620259-00091.

[ref40] Stambuli J. P.; Bühl M.; Hartwig J. F. Synthesis, Characterization, and Reactivity of Monomeric, Arylpalladium Halide Complexes with a Hindered Phosphine as the Only Dative Ligand. J. Am. Chem. Soc. 2002, 124, 9346–9347. 10.1021/ja0264394. 12167009

[ref41] Yamashita M.; Hartwig J. F. Synthesis, Structure, and Reductive Elimination Chemistry of Three-Coordinate Arylpalladium Amido Complexes. J. Am. Chem. Soc. 2004, 126, 5344–5345. 10.1021/ja0315107. 15113190

[ref42] Stambuli J. P.; Incarvito C. D.; Bühl M.; Hartwig J. F. Synthesis, Structure, Theoretical Studies, and Ligand Exchange Reactions of Monomeric, T-Shaped Arylpalladium(II) Halide Complexes with an Additional, Weak Agostic Interaction. J. Am. Chem. Soc. 2004, 126, 1184–1194. 10.1021/ja037928m. 14746489

[ref43] Hartwig J. F.; Paul F. Oxidative Addition of Aryl Bromide after Dissociation of Phosphine from a Two-Coordinate Palladium(0) Complex, Bis(tri-*o*-tolylphosphine)palladium(0). J. Am. Chem. Soc. 1995, 117, 5373–5374. 10.1021/ja00124a026.

[ref44] Brunel J. M. P(*t*-Bu)_3_: A Versatile and Efficient Ligand in Homogeneous Catalysis. Mini-Rev. Org. Chem. 2004, 1, 249–277. 10.2174/1570193043403163.

[ref45] Amatore C.; Pfluger F. Mechanism of Oxidative Addition of Palladium(0) with Aromatic Iodides in Toluene, Monitored at Ultramicroelectrodes. Organometallics 1990, 9, 2276–2282. 10.1021/om00158a026.

[ref46] Vikse K.; Naka T.; McIndoe J. S.; Besora M.; Maseras F. Oxidative Additions of Aryl Halides to Palladium Proceed through the Monoligated Complex. ChemCatChem. 2013, 5, 3604–3609. 10.1002/cctc.201300723.

[ref47] Ahlquist M.; Norrby P.-O. Oxidative Addition of Aryl Chlorides to Monoligated Palladium(0): A DFT-SCRF Study. Organometallics 2007, 26, 550–553. 10.1021/om0604932.

[ref48] Li Z.; Fu Y.; Guo Q.-X.; Liu L. Theoretical Study on Monoligated Pd-Catalyzed Cross-Coupling Reactions of Aryl Chlorides and Bromides. Organometallics 2008, 27, 4043–4049. 10.1021/om701065f.

[ref49] Barrios-Landeros F.; Carrow B. P.; Hartwig J. F. Effect of Ligand Steric Properties and Halide Identity on the Mechanism for Oxidative Addition of Haloarenes to Trialkylphosphine Pd(0) Complexes. J. Am. Chem. Soc. 2009, 131, 8141–8154. 10.1021/ja900798s. 19469511 PMC2885888

[ref50] Carrow B. P. Mechanistic Studies on Palladium-Catalyzed Coupling Reactions. Ph.D. Dissertation. University of Illinois at Urbana–Champaign, 2011; https://www.ideals.illinois.edu/bitstream/handle/2142/24376/Carrow_Bradley.pdf?sequence=1 (accessed 2020-12-27).

[ref51] Galardon E.; Ramdeehul S.; Brown J. M.; Cowley A.; Hii K. K.; Jutand A. Profound Steric Control of Reactivity in Aryl Halide Addition to Bisphosphane Palladium(0) Complexes. Angew. Chem., Int. Ed. 2002, 41, 1760–1763. 10.1002/1521-3773(20020517)41:10<1760::AID-ANIE1760>3.0.CO;2-3. 19750708

[ref52] McMullin C. L.; Jover J.; Harvey J. N.; Fey N. Accurate Modelling of Pd(0) + PhX Oxidative Addition Kinetics. Dalton Trans. 2010, 39, 10833–10836. 10.1039/c0dt00778a. 20963224

[ref53] Macgregor S. A.; Roe D. C.; Marshall W. J.; Bloch K. M.; Bakhmutov V. I.; Grushin V. V. The F/Ph Rearrangement Reaction of [(Ph_3_P)_3_RhF], the Fluoride Congener of Wilkinson’s Catalyst. J. Am. Chem. Soc. 2005, 127, 15304–15321. 10.1021/ja054506z. 16248673

[ref54] Hu H.; Qu F.; Gerlach D. L.; Shaughnessy K. L. Mechanistic Study of the Role of Substrate Steric Effects and Aniline Inhibition on the Bis(trineopentylphosphine)palladium(0)-Catalyzed Arylation of Aniline Derivatives. ACS Catal. 2017, 7, 2516–2527. 10.1021/acscatal.7b00024.

[ref55] Raders S. M.; Moore J. N.; Parks J. K.; Miller A. D.; Leißing T. M.; Kelley S. P.; Rogers R. D.; Shaughnessy K. H. Trineopentylphosphine: A Conformationally Flexible Ligand for the Coupling of Sterically Demanding Substrates in the Buchwald–Hartwig Amination and Suzuki–Miyaura Reaction. J. Org. Chem. 2013, 78, 4649–4664. 10.1021/jo400435z. 23638733

[ref56] Joshi C.; Macharia J. M.; Izzo J. A.; Wambua V.; Kim S.; Hirschi J. S.; Vetticatt M. J. Isotope Effects Reveal the Catalytic Mechanism of the Archetypical Suzuki–Miyaura Reaction. ACS Catal. 2022, 12, 2959–2966. 10.1021/acscatal.1c05802. PMC10168682 37168650

[ref57] Widenhoefer R. A.; Buchwald S. L. Electronic Dependence of C–O Reductive Elimination from Palladium (Aryl)neopentoxide Complexes. J. Am. Chem. Soc. 1998, 120, 6504–6511. 10.1021/ja9806581.

[ref58] Tatsumi K.; Hoffmann R.; Yamamoto A.; Stille J. K. Reductive Elimination of d^8^-Organotransition Metal Complexes. Bull. Chem. Soc. Jpn. 1981, 54, 1857–1867. 10.1246/bcsj.54.1857.

[ref59] Culkin D. A.; Hartwig J. F. Carbon–Carbon Bond-Forming Reductive Elimination from Arylpalladium Complexes Containing Functionalized Alkyl Groups. Influence of Ligand Steric and Electronic Properties on Structure, Stability, and Reactivity. Organometallics 2004, 23, 3398–3416. 10.1021/om049726k.

[ref60] Loar M. K.; Stille J. K. Mechanisms of 1,1-Reductive Elimination from Palladium: Coupling of Styrylmethylpalladium Complexes. J. Am. Chem. Soc. 1981, 103, 4174–4181. 10.1021/ja00404a033.

[ref61] Moravskiy A.; Stille J. K. Mechanisms of 1,1-Reductive Elimination from Palladium: Elimination of Ethane from Dimethylpalladium(II) and Trimethylpalladium(IV). J. Am. Chem. Soc. 1981, 103, 4182–4186. 10.1021/ja00404a034.

[ref62] Culkin D. A.; Hartwig J. F. Palladium-Catalyzed α-Arylation of Carbonyl Compounds and Nitriles. Acc. Chem. Res. 2003, 36, 234–245. 10.1021/ar0201106. 12693921

[ref63] Hartwig J. F. Carbon–Heteroatom Bond-Forming Reductive Eliminations of Amines, Ethers, and Sulfides. Acc. Chem. Res. 1998, 31, 852–860. 10.1021/ar970282g.

[ref64] Mann G.; Shelby Q.; Roy A. H.; Hartwig J. F. Electronic and Steric Effects on the Reductive Elimination of Diaryl Ethers from Palladium(II). Organometallics 2003, 22, 2775–2789. 10.1021/om030230x.

[ref65] Labinger J. A. Tutorial on Oxidative Addition. Organometallics 2015, 34, 4784–4795. 10.1021/acs.organomet.5b00565.

[ref66] Hartwig J. F. Organotransition Metal Chemistry: From Bonding to Catalysis; University Science Books: Sausalito, CA, 2010.

[ref67] Collman J. P.; Hegedus L. S.; Norton J. R.; Finke R. G. Principles and Applications of Organotransition Metal Chemistry, 2nd ed.; Oxford University Press: Oxford, UK, 1987.

[ref68] Hartwig J. F. Electronic Effects on Reductive Elimination to Form Carbon–Carbon and Carbon–Heteroatom Bonds from Palladium(II) Complexes. Inorg. Chem. 2007, 46, 1936–1947. 10.1021/ic061926w. 17348724

[ref69] Procelewska J.; Zahl A.; Liehr G.; van Eldik R.; Smythe N. A.; Williams B. S.; Goldberg K. L. Mechanistic Information on the Reductive Elimination from Cationic Trimethylplatinum(IV) Complexes to Form Carbon–Carbon Bonds. Inorg. Chem. 2005, 44, 7732–7742. 10.1021/ic050478+. 16241122

[ref70] Shekhar S.; Hartwig J. F. Distinct Electronic Effects on Reductive Eliminations of Symmetrical and Unsymmetrical Bis-Aryl Platinum Complexes. J. Am. Chem. Soc. 2004, 126, 13016–13027. 10.1021/ja0480365. 15469300

[ref71] Komiya S.; Albright T. A.; Hoffmann R.; Kochi J. K. Reductive Elimination and Isomerization of Organogold Complexes. Theoretical Studies of Trialkylgold Species as Reactive Intermediates. J. Am. Chem. Soc. 1976, 98, 7255–7265. 10.1021/ja00439a024.

[ref72] Labadie J. W.; Stille J. K. Mechanisms of the Palladium-Catalyzed Couplings of Acid Chlorides with Organotin Reagents. J. Am. Chem. Soc. 1983, 105, 6129–6137. 10.1021/ja00357a026.

[ref73] Tymonko S. A.; Smith R. C.; Ambrosi A.; Denmark S. E. Mechanistic Significance of the Si–O–Pd Bond in the Palladium-Catalyzed Cross-Coupling Reactions of Alkenylsilanolates. J. Am. Chem. Soc. 2015, 137, 6192–6199. 10.1021/jacs.5b02515. 25945390 PMC4442670

[ref74] Tymonko S. A.; Smith R. C.; Ambrosi A.; Ober M. H.; Wang H.; Denmark S. E. Mechanistic Significance of the Si–O–Pd Bond in the Palladium-Catalyzed Cross-Coupling Reactions of Arylsilanolates. J. Am. Chem. Soc. 2015, 137, 6200–6218. 10.1021/jacs.5b02518. 25945516 PMC4442671

[ref75] For the nomenclature, see Perkins C. W.; Martin J. C.; Arduengo A. J.; Lau W.; Alegria A.; Kochi J. K. An Electrically Neutral σ-Sulfuranyl Radical from the Homolysis of a Perester with Neighboring Sulfenyl Sulfur: 9-S-3 Species. J. Am. Chem. Soc. 1980, 102, 7753–7759. 10.1021/ja00546a019.

[ref76] Magano J.; Dunetz J. R. Large-Scale Applications of Transition Metal-Catalyzed Couplings for the Synthesis of Pharmaceuticals. Chem. Rev. 2011, 111, 2177–2250. 10.1021/cr100346g. 21391570

[ref77] Magano J.; Dunetz J. R. Recent Large-Scale Applications of Transition Metal-Catalyzed Couplings for the Synthesis of Pharmaceuticals. In New Trends in Cross-Coupling: Theory and Applications; RSC Catalysis Series No. 21; Colacot T. J., Hardcare C., Ismagilov Z., Ozkan U., Eds.; Royal Society of Chemistry: Cambridge, UK, 2014; Chapter 15, pp 697–778.

[ref78] Miyaura N.; Yamada K.; Suzuki A. A New Stereospecific Cross-Coupling by the Palladium-Catalyzed Reaction of 1-Alkenylboranes with 1-Alkenyl or 1-Alkynyl Halides. Tetrahedron Lett. 1979, 20, 3437–3440. 10.1016/S0040-4039(01)95429-2.

[ref79] Miyaura N.; Suzuki A. Stereoselective Synthesis of Arylated (*E*)-alkenes by the Reaction of Alk-1-enylboranes with Aryl Halides in the Presence of Palladium Catalyst. J. Chem. Soc., Chem. Commun. 1979, 866–867. 10.1039/c39790000866.

[ref80] Miyaura N.; Yamada K.; Suginome H.; Suzuki A. Novel and Convenient Method for the Stereo- and Regiospecific Synthesis of Conjugated Alkadienes and Alkenynes via the Palladium-Catalyzed Cross-Coupling Reaction of 1-Alkenylboranes with Bromoalkenes and Bromoalkynes. J. Am. Chem. Soc. 1985, 107, 972–980. 10.1021/ja00290a037.

[ref81] Matos K.; Soderquist J. A. Alkylboranes in the Suzuki–Miyaura Coupling: Stereochemical and Mechanistic Studies. J. Org. Chem. 1998, 63, 461–470. 10.1021/jo971681s. 11672034

[ref82] Amatore C.; Jutand A.; Le Duc G. Kinetic Data for the Transmetalation/Reductive Elimination in Palladium-Catalyzed Suzuki–Miyaura Reactions: Unexpected Triple Role of Hydroxide Ions Used as Base. Chem.—Eur. J. 2011, 17, 2492–2503. 10.1002/chem.201001911. 21319240

[ref83] Amatore C.; Jutand A.; Le Duc G. The Triple Role of Fluoride Ions in Palladium-Catalyzed Suzuki–Miyaura Reactions: Unprecedented Transmetalation from [ArPdFL_2_] Complexes. Angew. Chem., Int. Ed. 2012, 51, 1379–1382. 10.1002/anie.201107202. 22213630

[ref84] Schmidt A. F.; Kurokhtina A. A.; Larina E. V. Role of a Base in Suzuki-Miyaura Reaction. Russ. J. Gen. Chem. 2011, 81 (1573), 1573 10.1134/S1070363211070334.

[ref85] Carrow B. P.; Hartwig J. F. Distinguishing Between Pathways for Transmetalation in Suzuki–Miyaura Reactions. J. Am. Chem. Soc. 2011, 133, 2116–2119. 10.1021/ja1108326. 21280669 PMC3041853

[ref86] Sicre C.; Braga A. A. C.; Maseras F.; Cid M. M. Mechanistic Insights into the Transmetalation Step of a Suzuki–Miyaura Reaction of 2(4)-Bromopyridines: Characterization of an Intermediate. Tetrahedron 2008, 64, 7437–7443. 10.1016/j.tet.2008.05.018.

[ref87] Sumimoto M.; Iwane N.; Takahama T.; Sakaki S. Theoretical Study of Trans-metalation Process in Palladium-Catalyzed Borylation of Iodobenzene with Diboron. J. Am. Chem. Soc. 2004, 126, 10457–10471. 10.1021/ja040020r. 15315462

[ref88] Goossen L. J.; Koley D.; Hermann H. L.; Thiel W. The Palladium-Catalyzed Cross-Coupling Reaction of Carboxylic Anhydrides with Arylboronic Acids: A DFT Study. J. Am. Chem. Soc. 2005, 127, 11102–11114. 10.1021/ja052435y. 16076218

[ref89] Goossen L. J.; Koley D.; Hermann H. L.; Thiel W. Palladium Monophosphine Intermediates in Catalytic Cross-Coupling Reactions: A DFT Study. Organometallics 2006, 25, 54–67. 10.1021/om050685h.

[ref90] Lennox A. J. J.; Lloyd-Jones G. C. Transmetalation in the Suzuki–Miyaura Coupling: The Fork in the Trail. Angew. Chem., Int. Ed. 2013, 52, 7362–7370. 10.1002/anie.201301737. 23780626

[ref91] Ortuño M. A.; Lledós A.; Maseras F.; Ujaque G. The Transmetalation Process in Suzuki–Miyaura Reactions: Calculations Indicate Lower Barrier via Boronate Intermediate. ChemCatChem. 2014, 6, 3132–3138. 10.1002/cctc.201402326.

[ref92] Jover J.; Fey N.; Purdie M.; Lloyd-Jones G. C.; Harvey J. N. A Computational Study of Phosphine Ligand Effects in Suzuki–Miyaura Coupling. J. Mol. Catal. A: Chem. 2010, 324, 39–47. 10.1016/j.molcata.2010.02.021.

[ref93] Thomas A. A.; Wang H.; Zahrt A. F.; Denmark S. E. Structural, Kinetic, and Computational Characterization of the Elusive Arylpalladium(II)boronate Complexes in the Suzuki–Miyaura Reaction. J. Am. Chem. Soc. 2017, 139, 3805–3821. 10.1021/jacs.6b13384. 28266847 PMC7784246

[ref94] Thomas A. A.; Zahrt A. F.; Delaney C. P.; Denmark S. E. Elucidating the Role of the Boronic Esters in the Suzuki–Miyaura Reaction: Structural, Kinetic, and Computational Investigations. J. Am. Chem. Soc. 2018, 140, 4401–4416. 10.1021/jacs.8b00400. 29543441 PMC6008789

[ref95] Thomas A. A.; Denmark S. E. Pre-Transmetalation Intermediates in the Suzuki–Miyaura Reaction Revealed: The Missing Link. Science 2016, 352, 329–332. 10.1126/science.aad6981. 27081068

[ref96] Lennox A. J. J.; Lloyd-Jones G. C. Organotrifluoroborate Hydrolysis: Boronic Acid Release Mechanism and an Acid–Base Paradox in Cross-Coupling. J. Am. Chem. Soc. 2012, 134, 7431–7441. 10.1021/ja300236k. 22512340

[ref97] Lennox A. J. J.; Lloyd-Jones G. C. Preparation of Organotrifluoroborate Salts: Precipitation-Driven Equilibrium under Non-Etching Conditions. Angew. Chem., Int. Ed. 2012, 51, 9385–9388. 10.1002/anie.201203930. 22903850

[ref98] Butters M.; Harvey J. N.; Jover J.; Lennox A. J. J.; Lloyd-Jones G. C.; Murray M. Aryl Trifluoroborates in Suzuki–Miyaura Coupling: The Roles of Endogenous Aryl Boronic Acid and Fluoride. Angew. Chem., Int. Ed. 2010, 49, 5156–5160. 10.1002/anie.201001522. 20544767

[ref99] Gonzalez J. A.; Ogba O. M.; Morehouse G. F.; Rosson N.; Houk K. N.; Leach A. G.; Cheong P. H.-Y.; Burke M. D.; Lloyd-Jones G. C. MIDA Boronates Are Hydrolysed Fast and Slow by Two Different Mechanisms. Nat. Chem. 2016, 8, 1067–1075. 10.1038/nchem.2571. 27768100 PMC5115273

[ref100] Delaney C. P.; Kassel V. M.; Denmark S. E. Potassium Trimethylsilanolate Enables Rapid, Homogeneous Suzuki–Miyaura Cross-Coupling of Boronic Esters. ACS Catal. 2020, 10, 73–80. 10.1021/acscatal.9b04353. 33585070 PMC7880502

[ref101] Gildner P. G.; Colacot T. J. Reactions of the 21st Century: Two Decades of Innovative Catalyst Design for Palladium-Catalyzed Cross-Couplings. Organometallics 2015, 34, 5497–5508. 10.1021/acs.organomet.5b00567.

[ref102] Surry D. S.; Buchwald S. L. Dialkylbiaryl Phosphines in Pd-Catalyzed Amination: A User’s Guide. Chem. Sci. 2011, 2, 27–50. 10.1039/C0SC00331J. 22432049 PMC3306613

[ref103] Campeau L.-C.; Hazari N. Cross-Coupling and Related Reactions: Connecting Past Success to the Development of New Reactions for the Future. Organometallics 2019, 38, 3–35. 10.1021/acs.organomet.8b00720. 31741548 PMC6860378

[ref104] Golding W. A.; Schmitt H. L.; Phipps R. J. Systematic Variation of Ligand and Cation Parameters Enables Site-Selective C–C and C–N Cross-Coupling of Multiply Chlorinated Arenes through Substrate–Ligand Electrostatic Interactions. J. Am. Chem. Soc. 2020, 142, 21891–21898. 10.1021/jacs.0c11056. 33332114

[ref105] Palani V.; Hugelshofer C. L.; Kevlishvili I.; Liu P.; Sarpong R. A Short Synthesis of Delavatine A Unveils New Insights into Site-Selective Cross-Coupling of 3,5-Dibromo-2-pyrone. J. Am. Chem. Soc. 2019, 141, 2652–2660. 10.1021/jacs.8b13012. 30646686

[ref106] Wambua V.; Hirschi J. S.; Vetticatt M. J. Rapid Evaluation of the Mechanism of Buchwald–Hartwig Amination and Aldol Reactions Using Intramolecular ^13^C Kinetic Isotope Effects. ACS Catal. 2021, 11, 60–67. 10.1021/acscatal.0c04752. 34659873 PMC8516138

[ref107] Vidossich P.; Ujaque G.; Lledós A. Palladium Monophosphine Pd(PPh_3_): Is It Really Accessible in Solution?. Chem. Commun. 2014, 50, 661–663. 10.1039/C3CC47404F. 24281838

[ref108] Littke A. F.; Dai C.; Fu G. C. Versatile Catalysts for the Suzuki Cross-Coupling of Arylboronic Acids with Aryl and Vinyl Halides and Triflates under Mild Conditions. J. Am. Chem. Soc. 2000, 122, 4020–4028. 10.1021/ja0002058.

[ref109] Blanksby S. J.; Ellison G. B. Bond Dissociation Energies of Organic Molecules. Acc. Chem. Res. 2003, 36, 255–263. 10.1021/ar020230d. 12693923

[ref110] Wolfe J. P.; Wagaw S.; Marcoux J.-F.; Buchwald S. L. Rational Development of Practical Catalysts for Aromatic Carbon–Nitrogen Bond Formation. Acc. Chem. Res. 1998, 31, 805–818. 10.1021/ar9600650.

[ref111] Wolfe J. P.; Singer R. A.; Yang B. H.; Buchwald S. L. Highly Active Palladium Catalysts for Suzuki Coupling Reactions. J. Am. Chem. Soc. 1999, 121, 9550–9561. 10.1021/ja992130h.

[ref112] Kendall A. J.; Zakharov L. N.; Tyler D. R. Steric and Electronic Influences of Buchwald-Type Alkyl-JohnPhos Ligands. Inorg. Chem. 2016, 55, 3079–3090. 10.1021/acs.inorgchem.5b02996. 26913633

[ref113] Ingoglia B. T.; Wagen C. C.; Buchwald S. L. Biaryl Monophosphine Ligands in Palladium-Catalyzed C–N Coupling: An Updated User’s Guide. Tetrahedron 2019, 75, 4199–4211. 10.1016/j.tet.2019.05.003. 31896889 PMC6939672

[ref114] Ruiz-Castillo P.; Buchwald S. L. Applications of Palladium-Catalyzed C–N Cross-Coupling Reactions. Chem. Rev. 2016, 116, 12564–12649. 10.1021/acs.chemrev.6b00512. 27689804 PMC5070552

[ref115] Martin R.; Buchwald S. L. Palladium-Catalyzed Suzuki–Miyaura Cross-Coupling Reactions Employing Dialkylbiaryl Phosphine Ligands. Acc. Chem. Res. 2008, 41, 1461–1473. 10.1021/ar800036s. 18620434 PMC2645945

[ref116] Zapf A.; Ehrentraut A.; Beller M. A New Highly Efficient Catalyst System for the Coupling of Nonactivated and Deactivated Aryl Chlorides with Arylboronic Acids. Angew. Chem., Int. Ed. 2000, 39, 4153–4155. 10.1002/1521-3773(20001117)39:22<4153::AID-ANIE4153>3.0.CO;2-T. 11093237

[ref117] Aranyos A.; Old D. W.; Kiyomori A.; Wolfe J. P.; Sadighi J. P.; Buchwald S. L. Novel Electron-Rich Bulky Phosphine Ligands Facilitate the Palladium-Catalyzed Preparation of Diaryl Ethers. J. Am. Chem. Soc. 1999, 121, 4369–4378. 10.1021/ja990324r.

[ref118] Kolter M.; Böck K.; Karaghiosoff K.; Koszinowski K. Anionic Palladium(0) and Palladium(II) Ate Complexes. Angew. Chem., Int. Ed. 2017, 56, 13244–13248. 10.1002/anie.201707362. 28817225

[ref119] Zheng Q.; Liu Y.; Chen Q.; Hu M.; Helmy R.; Sherer E. C.; Welch C. J.; Chen H. Capture of Reactive Monophosphine-Ligated Palladium(0) Intermediates by Mass Spectrometry. J. Am. Chem. Soc. 2015, 137, 14035–14038. 10.1021/jacs.5b08905. 26498505

[ref120] Chen X.; Wei Z.; Huang K.-H.; Uehling M.; Wleklinski M.; Krska S.; Makarov A. A.; Nowak T.; Cooks R. G. Pd Reaction Intermediates in Suzuki-Miyaura Cross-Coupling Characterized by Mass Spectrometry. ChemPlusChem. 2022, 87, e202100545 10.1002/cplu.202100545. 35112808

[ref121] Christensen M.; Yunker L. P. E.; Adedeji F.; Häse F.; Roch L. M.; Gensch T.; dos Passos Gomes G.; Zepel T.; Sigman M. S.; Aspuru-Guzik A. Data-Science Driven Autonomous Process Optimization. Commun. Chem. 2021, 4, 112 10.1038/s42004-021-00550-x. PMC9814253 36697524

[ref122] Gensch T.; Smith S. R.; Colacot T. J.; Timsina Y. N.; Xu G.; Glasspoole B. W.; Sigman M. S. Design and Application of a Screening Set for Monophosphine Ligands in Cross-Coupling. ACS Catal. 2022, 12, 7773–7780. 10.1021/acscatal.2c01970.

[ref123] Gensch T.; dos Passos Gomes G.; Friederich P.; Peters E.; Gaudin T.; Pollice R.; Jorner K.; Nigam A.; Lindner-D’Addario M.; Sigman M. S.; et al. A Comprehensive Discovery Platform for Organophosphorus Ligands for Catalysis. J. Am. Chem. Soc. 2022, 144, 1205–1217. 10.1021/jacs.1c09718. 35020383

[ref124] Tolman C. A. Steric Effects of Phosphorus Ligands in Organometallic Chemistry and Homogeneous Catalysis. Chem. Rev. 1977, 77, 313–348. 10.1021/cr60307a002.

[ref125] Müller T. E.; Mingos D. M. P. Determination of the Tolman Cone Angle from Crystallographic Parameters and a Statistical Analysis Using the Crystallographic Data Base. Transition Met. Chem. 1995, 20, 533–539. 10.1007/BF00136415.

[ref126] Lundgren R. J.; Stradiotto M. Key Concepts in Ligand Design: An Introduction. In Ligand Design in Metal Chemistry: Reactivity and Catalysis; Stradiotto M., Lundgren R. J., Eds.; Wiley: Hoboken, NJ, 2016; Chapter 1, pp 1–14.

[ref127] Jover J.; Cirera J. Computational Assessment on the Tolman Cone Angles for P-Ligands. Dalton Trans. 2019, 48, 15036–15048. 10.1039/C9DT02876E. 31513203

[ref128] Bilbrey J. A.; Kazez A. H.; Locklin J.; Allen W. D. Exact Ligand Cone Angles. J. Comput. Chem. 2013, 34, 1189–1197. 10.1002/jcc.23217. 23408559

[ref129] Newman-Stonebraker S. H.; Smith S. R.; Borowski J. E.; Peters E.; Gensch T.; Johnson H. C.; Sigman M. S.; Doyle A. G. Univariate Classification of Phosphine Ligation State and Reactivity in Cross-Coupling Catalysis. Science 2021, 374, 301–308. 10.1126/science.abj4213. 34648340

[ref130] Wu K.; Doyle A. G. Parameterization of Phosphine Ligand Demonstrates Enhancement of Nickel Catalysis via Remote Steric Effects. Nat. Chem. 2017, 9, 779–784. 10.1038/nchem.2741. 28754948 PMC5609847

[ref131] Niemeyer Z. L.; Milo A.; Hickey D. P.; Sigman M. S. Parameterization of Phosphine Ligands Reveals Mechanistic Pathways and Predicts Reaction Outcomes. Nat. Chem. 2016, 8, 610–617. 10.1038/nchem.2501. 27219707

[ref132] Schoenebeck F.; Houk K. N. Ligand-Controlled Regioselectivity in Palladium-Catalyzed Cross Coupling Reactions. J. Am. Chem. Soc. 2010, 132, 2496–2497. 10.1021/ja9077528. 20121156

[ref133] Zapf A.; Beller M. Palladium Catalyst Systems for Cross-Coupling Reactions of Aryl Chlorides and Olefins. Chem.—Eur. J. 2001, 7, 2908–2915. 10.1002/1521-3765(20010702)7:13<2908::AID-CHEM2908>3.0.CO;2-R. 11486967

[ref134] Hueffel J. A.; Sperger T.; Funes-Ardoiz I.; Ward J. S.; Rissanen K.; Schoenebeck F. Accelerated Dinuclear Palladium Catalyst Identification Through Unsupervised Machine Learning. Science 2021, 374, 1134–1140. 10.1126/science.abj0999. 34822285

[ref135] Shaughnessy K. H. Development of Palladium Precatalysts That Efficiently Generate LPd(0) Active Species. Isr. J. Chem. 2020, 60, 180–194. 10.1002/ijch.201900067.

[ref136] Christmann U.; Vilar R. Monoligated Palladium Species as Catalysts in Cross-Coupling Reactions. Angew. Chem., Int. Ed. 2005, 44, 366–374. 10.1002/anie.200461189. 15624192

[ref137] Lau S. H.; Chen L.; Davis K.; Kevlishvili I.; Liu P.; Carrow B. P. Capturing the Most Active State of a Palladium(0) Cross-Coupling Catalyst. ChemRxiv; Cambridge University Press, 2021; 10.33774/chemrxiv-2021-477kn).

[ref138] Lee C.-L.; James B. R.; Nelson D. A.; Hallen R. T. Kinetics and Thermodynamics of the Reversible Reaction between Carbon Monoxide and Palladium(I) Dimers Containing Bis(diphenyIphosphino)methane. Organometallics 1984, 3, 1360–1364. 10.1021/om00087a007.

[ref139] For a recent review on Pd(I) dimers, see Fricke C.; Sperger T.; Mendel M.; Schoenebeck F. Catalysis with Palladium(I) Dimers. Angew. Chem., Int. Ed. 2021, 60, 3355–3366. 10.1002/anie.202011825. PMC7898807 33058375

[ref140] Inatomi T.; Koga Y.; Matsubara K. Dinuclear Nickel(I) and Palladium(I) Complexes for Highly Active Transformations of Organic Compounds. Molecules 2018, 23, 140 10.3390/molecules23010140. 29324677 PMC6017577

[ref141] Murahashi T.; Kurosawa H. Organopalladium Complexes Containing Palladium–Palladium Bonds. Coord. Chem. Rev. 2002, 231, 207–228. 10.1016/S0010-8545(02)00121-2.

[ref142] Chalkley M. J.; Guard L. M.; Hazari N.; Hofmann P.; Hruszkewycz D. P.; Schmeier T. J.; Takase M. K. Synthesis, Electronic Structure, and Reactivity of Palladium(I) Dimers with Bridging Allyl, Cyclopentadienyl, and Indenyl Ligands. Organometallics 2013, 32, 4223–4238. 10.1021/om400415c.

[ref143] Hazari N.; Hruszkewycz D. P. Dinuclear Pd(I) Complexes with Bridging Allyl and Related Ligands. Chem. Soc. Rev. 2016, 45, 2871–2899. 10.1039/C5CS00537J. 27051890

[ref144] Xu W.; Li M.; Qiao L.; Xie J. Recent Advances of Dinuclear Nickel- and Palladium-Complexes in Homogeneous Catalysis. Chem. Commun. 2020, 56, 8524–8536. 10.1039/D0CC02542A. 32613965

[ref145] Colacot T. J. A Highly Active Palladium(I) Dimer for Pharmaceutical Applications: [Pd(μ-Br)(tBu_3_P)]_2_ as a Practical Cross-Coupling Catalyst. Platinum Metals Rev. 2009, 53, 183–188. and references therein. 10.1595/147106709X472147.

[ref146] Vilar R.; Mingos D. M. P.; Cardin C. J. Synthesis and Structural Characterisation of [Pd_2_(μ-Br)_2_(PBu^*t*^_3_)_2_], an Example of a Palladium(1)–Palladium(1) Dimer. J. Chem. Soc., Dalton Trans. 1996, 4313–4314. 10.1039/DT9960004313.

[ref147] Durà-Vilà V.; Mingos D. M. P.; Vilar R.; White A. J. P.; Williams D. J. Reactivity Studies of [Pd_2_(μ-X)_2_(PBu^*t*^_3_)_2_] (X = Br, I) with CNR (R = 2,6-Dimethylphenyl), H_2_ and Alkynes. J. Organomet. Chem. 2000, 600, 198–205. 10.1016/S0022-328X(00)00187-X.

[ref148] Stambuli J. P.; Kuwano R.; Hartwig J. F. Unparalleled Rates for the Activation of Aryl Chlorides and Bromides: Coupling with Amines and Boronic Acids in Minutes at Room Temperature. Angew. Chem., Int. Ed. 2002, 41, 4746–4748. 10.1002/anie.200290036. 12481346

[ref149] Kuwano R.; Utsunomiya M.; Hartwig J. F. Aqueous Hydroxide as a Base for Palladium-Catalyzed Amination of Aryl Chlorides and Bromides. J. Org. Chem. 2002, 67, 6479–6486. 10.1021/jo0258913. 12201770

[ref150] Prashad M.; Mak X. Y.; Liu Y.; Repič O. Palladium-Catalyzed Amination of Aryl Bromides with Hindered N-Alkyl-Substituted Anilines Using a Palladium(I) Tri-*tert*-butylphosphine Bromide Dimer. J. Org. Chem. 2003, 68, 1163–1164. 10.1021/jo020609d. 12558453

[ref151] Mamone P.; Grünberg M. F.; Fromm A.; Khan B. A.; Gooßen L. J. [Pd(μ-Br)(P^*t*^Bu_3_)]_2_ as a Highly Active Isomerization Catalyst: Synthesis of Enol Esters from Allylic Esters. Org. Lett. 2012, 14, 3716–3719. 10.1021/ol301563g. 22747236

[ref152] Powers I. G.; Uyeda C. Metal–Metal Bonds in Catalysis. ACS Catal. 2017, 7, 936–958. 10.1021/acscatal.6b02692.

[ref153] Pye D. R.; Mankad N. P. Bimetallic Catalysis for C–C and C–X Coupling Reactions. Chem. Sci. 2017, 8, 1705–1718. 10.1039/C6SC05556G. 29780450 PMC5933431

[ref154] Proutiere F.; Aufiero M.; Schoenebeck F. Reactivity and Stability of Dinuclear Pd(I) Complexes: Studies on the Active Catalytic Species, Insights into Precatalyst Activation and Deactivation, and Application in Highly Selective Cross-Coupling Reactions. J. Am. Chem. Soc. 2012, 134, 606–612. 10.1021/ja209424z. 22132686

[ref155] Barrios-Landeros F.; Carrow B. P.; Hartwig J. F. Autocatalytic Oxidative Addition of PhBr to Pd(PtBu_3_)_2_ via Pd(PtBu_3_)_2_(H)(Br). J. Am. Chem. Soc. 2008, 130, 5842–5843. 10.1021/ja711159y. 18402444 PMC2828772

[ref156] Colacot T. J.; Hooper M. W.; Grasa G. A. Process for the Preparation of Palladium (I) Tri-*tert*-butylphosphine Bromide Dimer. World Patent WO2011/012889, January 3, 2011; https://patentscope.wipo.int/search/en/detail.jsf?docId=WO2011012889&tab=PCTBIBLIO (accessed 2021-02-15).

[ref157] Carole W.; Colacot T.; Seechurn C.; Scrase T. Process. World Patent WO2018073559, April 26, 2018; https://patentscope.wipo.int/search/en/detail.jsf?docId=WO2018073559 (accessed 2021-02-15).

[ref158] Goossen L.; Arndt M.; Mamone P.; Gruenberg M. Method for the Preparation of Palladium(I) Tri-*tert*-butylphosphine Bromide Dimer and Process for Its Use in Isomerization Reactions. World Patent WO2013/000874, January 3, 2013; https://patentscope.wipo.int/search/en/detail.jsf?docId=WO2013000874&tab=PCTBIBLIO (accessed 2021-02-15).

[ref159] Aufiero M.; Sperger T.; Tsang A. S.-K.; Schoenebeck F. Highly Efficient C–SeCF_3_ Coupling of Aryl Iodides Enabled by an Air-Stable Dinuclear Pd^I^ Catalyst. Angew. Chem., Int. Ed. 2015, 54, 10322–10326. 10.1002/anie.201503388. 26118426

[ref160] Buscemi G.; Miller P. W.; Kealey S.; Gee A. D.; Long N. J.; Passchier J.; Vilar R. Rapid Carbonylative Coupling Reactions Using Palladium(I) Dimers: Applications to ^11^CO-Radiolabelling for the Synthesis of PET Tracers. Org. Biomol. Chem. 2011, 9, 3499–3503. 10.1039/c1ob05268c. 21431235

[ref161] Aufiero M.; Scattolin T.; Proutière F.; Schoenebeck F. Air-Stable Dinuclear Iodine-Bridged Pd(I) Complex - Catalyst, Precursor, or Parasite? The Additive Decides. Systematic Nucleophile-Activity Study and Application as Precatalyst in Cross-Coupling. Organometallics 2015, 34, 5191–5195. 10.1021/acs.organomet.5b00766.

[ref162] Mayr H.; Ofial A. R. Do General Nucleophilicity Scales Exist?. J. Phys. Org. Chem. 2008, 21, 584–595. 10.1002/poc.1325.

[ref163] Baidya M.; Kobayashi S.; Brotzel F.; Schmidhammer U.; Riedle E.; Mayr H. DABCO and DMAP—Why Are They Different in Organocatalysis?. Angew. Chem., Int. Ed. 2007, 46, 6176–6179. 10.1002/anie.200701489. 17628470

[ref164] Fricke C.; Sherborne G. J.; Funes-Ardoiz I.; Senol E.; Guven S.; Schoenebeck F. Orthogonal Nanoparticle Catalysis with Organogermanes. Angew. Chem., Int. Ed. 2019, 58, 17788–17795. 10.1002/anie.201910060. PMC6899604 31562670

[ref165] Bonney K. J.; Proutiere F.; Schoenebeck F. Dinuclear Pd(I) Complexes—Solely Precatalysts? Demonstration of Direct Reactivity of a Pd(I) Dimer with an Aryl Iodide. Chem. Sci. 2013, 4, 4434–4439. 10.1039/c3sc52054d.

[ref166] Kalvet I.; Bonney K. J.; Schoenebeck F. Kinetic and Computational Studies on Pd(I) Dimer-Mediated Halogen Exchange of Aryl Iodides. J. Org. Chem. 2014, 79, 12041–12046. 10.1021/jo501889j. 25247472

[ref167] Barnett K. L.; Howard J. R.; Treager C. J.; Shipley A. T.; Stullich R. M.; Qu F.; Gerlach D. L.; Shaughnessy K. H. Air-Stable [(R_3_P)PdCl_2_]_2_ Complexes of Neopentylphosphines as Cross-Coupling Precatalysts: Catalytic Application and Mechanism of Catalyst Activation and Deactivation. Organometallics 2018, 37, 1410–1424. 10.1021/acs.organomet.8b00082.

[ref168] Krause S. B.; McAtee J. R.; Yap G. P. A.; Watson D. A. A Bench-Stable, Single-Component Precatalyst for Silyl–Heck Reactions. Org. Lett. 2017, 19, 5641–5644. 10.1021/acs.orglett.7b02807. 28960083 PMC5983356

[ref169] Viciu M. S.; Kissling R. M.; Stevens E. D.; Nolan S. P. An Air-Stable Palladium/*N*-Heterocyclic Carbene Complex and Its Reactivity in Aryl Amination. Org. Lett. 2002, 4, 2229–2231. 10.1021/ol0260831. 12074674

[ref170] Diebolt O.; Braunstein P.; Nolan S. P.; Cazin C. S. J. Room-Temperature Activation of Aryl Chlorides in Suzuki–Miyaura Coupling using a [Pd(μ-Cl)Cl(NHC)]_2_ Complex (NHC = N-Heterocyclic Carbene). Chem. Commun. 2008, 3190–3192. 10.1039/b804695f. 18594738

[ref171] Hartmann C. E.; Nolan S. P.; Cazin C. S. J. Highly Active [Pd(μ-Cl)(Cl)(NHC)]_2_ (NHC = N-Heterocyclic Carbene) in the Cross-Coupling of Grignard Reagents with Aryl Chlorides. Organometallics 2009, 28, 2915–2919. 10.1021/om900072f.

[ref172] Tessin U. I.; Bantreil X.; Songis O.; Cazin C. S. J. Highly Active [Pd(μ-Cl)Cl(NHC)]_2_ Complexes in the Mizoroki–Heck Reaction. Eur. J. Inorg. Chem. 2013, 2013, 2007–2010. 10.1002/ejic.201300169.

[ref173] Ostrowska S.; Lorkowski L.; Kubicki M.; Pietraszuk C. [{Pd(μ-OH)Cl(IPr)}_2_]—A Highly Efficient Precatalyst for Suzuki–Miyaura Coupling also Able to Act under Base-Free Conditions. ChemCatChem. 2016, 8, 3580–3583. 10.1002/cctc.201600998.

[ref174] Herrmann W. A.; Öfele K.; v. Preysing D.; Schneider S. K. Phospha-Palladacycles and *N*-Heterocyclic Carbene Palladium Complexes: Efficient Catalysts for CC-Coupling Reactions. J. Organomet. Chem. 2003, 687, 229–248. 10.1016/j.jorganchem.2003.07.028.

[ref175] Bedford R. B. Palladacyclic Catalysts in C–C and C–Heteroatom Bond-Forming Reactions. Chem. Commun. 2003, 1787–1796. 10.1039/B211298C. 12931974

[ref176] Dupont J.; Consorti C. S.; Spencer J. The Potential of Palladacycles: More Than Just Precatalysts. Chem. Rev. 2005, 105, 2527–2571. 10.1021/cr030681r. 15941221

[ref177] Mo D.-L.; Zhang T.-K.; Ge G.-C.; Huang X.-J; Ding C.-H.; Dai L.-X.; Hou X.-L. The Applications of Palladacycles as Transition-Metal Catalysts in Organic Synthesis. Synlett 2014, 25, 2686–2702. 10.1055/s-0034-1379230.

[ref178] Nájera C. Oxime-Derived Palladacycles: Applications in Catalysis. ChemCatChem. 2016, 8, 1865–1881. 10.1002/cctc.201600035.

[ref179] Bruneau A.; Roche M.; Alami M.; Messaoudi S. 2-Aminobiphenyl Palladacycles: The “Most Powerful” Precatalysts in C–C and C–Heteroatom Cross-Couplings. ACS Catal. 2015, 5, 1386–1396. 10.1021/cs502011x.

[ref180] Navarro O.; Kelly R. A. III; Nolan S. P. A General Method for the Suzuki–Miyaura Cross-Coupling of Sterically Hindered Aryl Chlorides: Synthesis of Di- and Tri-ortho-substituted Biaryls in 2-Propanol at Room Temperature. J. Am. Chem. Soc. 2003, 125, 16194–16195. 10.1021/ja038631r. 14692753

[ref181] Viciu M. S.; Kelly R. A. III; Stevens E. D.; Naud F.; Studer M.; Nolan S. P. Synthesis, Characterization, and Catalytic Activity of N-Heterocyclic Carbene (NHC) Palladacycle Complexes. Org. Lett. 2003, 5, 1479–1482. 10.1021/ol034264c. 12713303

[ref182] Navarro O.; Marion N.; Oonishi Y.; Kelly R. A. III; Nolan S. P. Suzuki–Miyaura, α-Ketone Arylation and Dehalogenation Reactions Catalyzed by a Versatile *N*-Heterocyclic Carbene–Palladacycle Complex. J. Org. Chem. 2006, 71, 685–692. 10.1021/jo0521201. 16408981

[ref183] Broggi J.; Clavier H.; Nolan S. P. N-Heterocyclic Carbenes (NHCs) Containing N*-*C*-*Palladacycle Complexes: Synthesis and Reactivity in Aryl Amination Reactions. Organometallics 2008, 27, 5525–5531. 10.1021/om8006689.

[ref184] Biscoe M. R.; Fors B. P.; Buchwald S. L. A New Class of Easily Activated Palladium Precatalysts for Facile C–N Cross-Coupling Reactions and the Low Temperature Oxidative Addition of Aryl Chlorides. J. Am. Chem. Soc. 2008, 130, 6686–6687. 10.1021/ja801137k. 18447360 PMC2587037

[ref185] Kinzel T.; Zhang Y.; Buchwald S. L. A New Palladium Precatalyst Allows for the Fast Suzuki–Miyaura Coupling Reactions of Unstable Polyfluorophenyl and 2-Heteroaryl Boronic Acids. J. Am. Chem. Soc. 2010, 132, 14073–14075. 10.1021/ja1073799. 20858009 PMC2953245

[ref186] Bruno N. C.; Tudge M. T.; Buchwald S. L. Design and Preparation of New Palladium Precatalysts for C–C and C–N Cross-Coupling Reactions. Chem. Sci. 2013, 4, 916–920. 10.1039/C2SC20903A. 23667737 PMC3647481

[ref187] DeAngelis A. J.; Gildner P. G.; Chow R.; Colacot T. J. Generating Active “L-Pd(0)” via Neutral or Cationic π-Allylpalladium Complexes Featuring Biaryl/Bipyrazolylphosphines: Synthetic, Mechanistic, and Structure–Activity Studies in Challenging Cross-Coupling Reactions. J. Org. Chem. 2015, 80, 6794–6813. 10.1021/acs.joc.5b01005. 26035637

[ref188] Bruno N. C.; Niljianskul N.; Buchwald S. L. *N*-Substituted 2-Aminobiphenylpalladium Methanesulfonate Precatalysts and Their Use in C–C and C–N Cross-Couplings. J. Org. Chem. 2014, 79, 4161–4166. 10.1021/jo500355k. 24724692 PMC4017611

[ref189] Chen L.; Ren P.; Carrow B. P. Tri(1-adamantyl)phosphine: Expanding the Boundary of Electron-Releasing Character Available to Organophosphorus Compounds. J. Am. Chem. Soc. 2016, 138, 6392–6395. 10.1021/jacs.6b03215. 27164163

[ref190] Peh G.-R.; Kantchev E. A. B.; Er J.-C.; Ying J. Y. Rational Exploration of N-Heterocyclic Carbene (NHC) Palladacycle Diversity: A Highly Active and Versatile Precatalyst for Suzuki–Miyaura Coupling Reactions of Deactivated Aryl and Alkyl Substrates. Chem.—Eur. J. 2010, 16, 4010–4017. 10.1002/chem.200902842. 20175159

[ref191] Hill L. L.; Crowell J. L.; Tutwiler S. L.; Massie N. L.; Hines C. C.; Griffin S. T.; Rogers R. D.; Shaughnessy K. H.; Grasa G. A.; Johansson Seechurn C. C. C.; et al. Synthesis and X-ray Structure Determination of Highly Active Pd(II), Pd(I), and Pd(0) Complexes of Di(*tert*-butyl)neopentylphosphine (DTBNpP) in the Arylation of Amines and Ketones. J. Org. Chem. 2010, 75, 6477–6488. 10.1021/jo101187q. 20806983

[ref192] Reddy C. V.; Kingston J. V.; Verkade J. G. (*t*-Bu)_2_PN = P(*i*-BuNCH_2_CH_2_)_3_N: New Efficient Ligand for Palladium-Catalyzed C–N Couplings of Aryl and Heteroaryl Bromides and Chlorides and for Vinyl Bromides at Room Temperature. J. Org. Chem. 2008, 73, 3047–3062. 10.1021/jo702367k. 18370424

[ref193] Johansson Seechurn C. C. C.; Parisel S. L.; Colacot T. J. Air-Stable Pd(R-allyl)LCl (L = Q-Phos, P(*t*-Bu)_3_, etc.) Systems for C–C/N Couplings: Insight into the Structure–Activity Relationship and Catalyst Activation Pathway. J. Org. Chem. 2011, 76, 7918–7932. 10.1021/jo2013324. 21823586

[ref194] Marion N.; Navarro O.; Mei J.; Stevens E. D.; Scott N. M.; Nolan S. P. Modified (NHC)Pd(allyl)Cl (NHC = *N*-Heterocyclic Carbene) Complexes for Room-Temperature Suzuki–Miyaura and Buchwald–Hartwig Reactions. J. Am. Chem. Soc. 2006, 128, 4101–4111. 10.1021/ja057704z. 16551119

[ref195] Hruszkewycz D. P.; Balcells D.; Guard L. M.; Hazari N.; Tilset M. Insight into the Efficiency of Cinnamyl-Supported Precatalysts for the Suzuki–Miyaura Reaction: Observation of Pd(I) Dimers with Bridging Allyl Ligands During Catalysis. J. Am. Chem. Soc. 2014, 136, 7300–7316. 10.1021/ja412565c. 24824779

[ref196] Hruszkewycz D. P.; Guard L. M.; Balcells D.; Feldman N.; Hazari N.; Tilset M. Effect of 2-Substituents on Allyl-Supported Precatalysts for the Suzuki–Miyaura Reaction: Relating Catalytic Efficiency to the Stability of Palladium(I) Bridging Allyl Dimers. Organometallics 2015, 34, 381–394. 10.1021/om501250y.

[ref197] Wu L.; Drinkel E.; Gaggia F.; Capolicchio S.; Linden A.; Falivene L.; Cavallo L.; Dorta R. Room-Temperature Synthesis of Tetra-ortho-Substituted Biaryls by NHC-Catalyzed Suzuki–Miyaura Couplings. Chem.—Eur. J. 2011, 17, 12886–12890. 10.1002/chem.201102442. 21984486

[ref198] Chartoire A.; Lesieur M.; Falivene L.; Slawin A. M. Z.; Cavallo L.; Cazin C. S. J.; Nolan S. P. [Pd(IPr*)(cinnamyl)Cl]: An Efficient Pre-catalyst for the Preparation of Tetra-*ortho*-substituted Biaryls by Suzuki–Miyaura Cross-Coupling. Chem.—Eur. J. 2012, 18, 4517–4521. 10.1002/chem.201104009. 22415936

[ref199] Melvin P. R.; Nova A.; Balcells D.; Dai W.; Hazari N.; Hruszkewycz D. P.; Shah H. P.; Tudge M. T. Design of a Versatile and Improved Precatalyst Scaffold for Palladium-Catalyzed Cross-Coupling: (η^3^-1-^t^Bu-indenyl)_2_(μ-Cl)_2_Pd_2_. ACS Catal. 2015, 5 (6), 3680–3688. 10.1021/acscatal.5b00878.

[ref200] Kang J.; Kim K. S. *N*-Cyclopropylation of Aromatic Amines. J. Chem. Soc., Chem. Commun. 1987, 897–898. 10.1039/c39870000897.

[ref201] Cui W.; Loeppky R. N. The Synthesis of *N*-Arylcyclopropylamines via Palladium-Catalyzed C–N Bond Formation. Tetrahedron 2001, 57, 2953–2956. 10.1016/S0040-4020(01)00118-1.

[ref202] Bénard S.; Neuville L.; Zhu J. Copper-Promoted *N*-Cyclopropylation of Anilines and Amines by Cyclopropylboronic Acid. Chem. Commun. 2010, 46, 3393–3395. 10.1039/b925499d. 20361101

[ref203] Gildner P. G.; DeAngelis A.; Colacot T. J. Palladium-Catalyzed *N*-Arylation of Cyclopropylamines. Org. Lett. 2016, 18, 1442–1445. 10.1021/acs.orglett.6b00377. 26934493

[ref204] Tassone J. P.; MacQueen P. M.; Lavoie C. M.; Ferguson M. J.; McDonald R.; Stradiotto M. Nickel-Catalyzed *N*-Arylation of Cyclopropylamine and Related Ammonium Salts with (Hetero)aryl (Pseudo)halides at Room Temperature. ACS Catal. 2017, 7, 6048–6059. 10.1021/acscatal.7b02014.

[ref205] Mikhailine A. A.; Grasa Mannino G. A.; Colacot T. J. Catalyst-Directed Chemoselective Double Amination of Bromo-chloro(hetero)arenes: A Synthetic Route toward Advanced Amino-aniline Intermediates. Org. Lett. 2018, 20, 2301–2305. 10.1021/acs.orglett.8b00646. 29620906

[ref206] O’Brien C. J.; Kantchev E. A. B.; Valente C.; Hadei N.; Chass G. A.; Lough A.; Hopkinson A. C.; Organ M. G. Easily Prepared Air- and Moisture-Stable Pd–NHC (NHC = N-Heterocyclic Carbene) Complexes: A Reliable, User-Friendly, Highly Active Palladium Precatalyst for the Suzuki–Miyaura Reaction. Chem.—Eur. J. 2006, 12, 4743–4748. 10.1002/chem.200600251. 16568494

[ref207] PEPPSI Catalysts; MilliporeSigma, 2022; https://www.sigmaaldrich.com/chemistry/chemical-synthesis/technology-spotlights/peppsi.html (accessed 2022-02-03).

[ref208] PEPPSI: Instructions for Use; MilliporeSigma, 2022; https://www.sigmaaldrich.com/content/dam/sigma-aldrich/docs/Aldrich/Bulletin/al_peppsi_activation_guide.pdf (accessed 2022-02-03).

[ref209] Nasielski J.; Hadei N.; Achonduh G.; Kantchev E. A. B.; O’Brien C. J.; Lough A.; Organ M. G. Structure–Activity Relationship Analysis of Pd–PEPPSI Complexes in Cross-Couplings: A Close Inspection of the Catalytic Cycle and the Precatalyst Activation Model. Chem.—Eur. J. 2010, 16, 10844–10853. 10.1002/chem.201000138. 20665575

[ref210] Chartoire A.; Frogneux X.; Boreux A.; Slawin A. M. Z.; Nolan S. P. [Pd(IPr*)(3-Cl-pyridinyl)Cl_2_]: A Novel and Efficient PEPPSI Precatalyst. Organometallics 2012, 31, 6947–6951. 10.1021/om300725f.

[ref211] Sharif S.; Rucker R. P.; Chandrasoma N.; Mitchell D.; Rodriguez M. J.; Froese R. D. J.; Organ M. G. Selective Monoarylation of Primary Amines Using the Pd-PEPPSI-IPent(Cl) Precatalyst. Angew. Chem., Int. Ed. 2015, 54, 9507–9511. 10.1002/anie.201502822. 26097000

[ref212] Hoi K. H.; Coggan J. A.; Organ M. G. Pd-PEPPSI-IPent^Cl^: An Effective Catalyst for the Preparation of Triarylamines. Chem.—Eur. J. 2013, 19, 843–845. 10.1002/chem.201203379. 23255429

[ref213] Pompeo M.; Froese R. D. J.; Hadei N.; Organ M. G. Pd-PEPPSI-IPent^Cl^: A Highly Effective Catalyst for the Selective Cross-Coupling of Secondary Organozinc Reagents. Angew. Chem., Int. Ed. 2012, 51, 11354–11357. 10.1002/anie.201205747. 23038603

[ref214] Atwater B.; Chandrasoma N.; Mitchell D.; Rodriguez M. J.; Organ M. G. Pd-PEPPSI-IHept^Cl^: A General-Purpose, Highly Reactive Catalyst for the Selective Coupling of Secondary Alkyl Organozincs. Chem.—Eur. J. 2016, 22, 14531–14534. 10.1002/chem.201603603. 27481602

[ref215] Zhang Y.; Lavigne G.; César V. Buchwald–Hartwig Amination of (Hetero)Aryl Tosylates Using a Well-Defined N-Heterocyclic Carbene/Palladium(II) Precatalyst. J. Org. Chem. 2015, 80, 7666–7673. 10.1021/acs.joc.5b01272. 26161731

[ref216] Lan X.-B.; Li Y.; Li Y.-F.; Shen D.-S.; Ke Z.; Liu F.-S. Flexible Steric Bulky Bis(Imino)acenaphthene (BIAN)-Supported N-Heterocyclic Carbene Palladium Precatalysts: Catalytic Application in Buchwald–Hartwig Amination in Air. J. Org. Chem. 2017, 82, 2914–2925. 10.1021/acs.joc.6b02867. 28244313

[ref217] Ouyang J.-S.; Li Y.-F.; Huang F.-D.; Lu D.-D.; Liu F.-S. The Highly Efficient Suzuki–Miyaura Cross-Coupling of (Hetero)aryl Chlorides and (Hetero)arylboronic Acids Catalyzed by “Bulky-yet-Flexible” Palladium–PEPPSI Complexes in Air. ChemCatChem. 2018, 10, 371–375. 10.1002/cctc.201701076.

[ref218] Lei P.; Meng G.; Ling Y.; An J.; Szostak M. Pd-PEPPSI: Pd-NHC Precatalyst for Suzuki–Miyaura Cross-Coupling Reactions of Amides. J. Org. Chem. 2017, 82, 6638–6646. 10.1021/acs.joc.7b00749. 28654258

[ref219] Lei P.; Meng G.; Szostak M. General Method for the Suzuki–Miyaura Cross-Coupling of Amides Using Commercially Available, Air- and Moisture-Stable Palladium/NHC (NHC = N-Heterocyclic Carbene) Complexes. ACS Catal. 2017, 7, 1960–1965. 10.1021/acscatal.6b03616.

[ref220] Shi S.; Lei P.; Szostak M. Pd-PEPPSI: A General Pd-NHC Precatalyst for Suzuki–Miyaura Cross-Coupling of Esters by C–O Cleavage. Organometallics 2017, 36, 3784–3789. 10.1021/acs.organomet.7b00565.

[ref221] Yang S.; Zhou T.; Poater A.; Cavallo L.; Nolan S. P.; Szostak M. Suzuki–Miyaura Cross-Coupling of Esters by Selective O–C(O) Cleavage Mediated by Air- and Moisture-Stable [Pd(NHC)(μ-Cl)Cl]_2_ Precatalysts: Catalyst Evaluation and Mechanism. Catal. Sci. Technol. 2021, 11, 3189–3197. 10.1039/D1CY00312G. 34211698 PMC8240519

[ref222] Shi S.; Szostak M. Pd–PEPPSI: A General Pd–NHC Precatalyst for Buchwald–Hartwig Cross-Coupling of Esters and Amides (Transamidation) Under the Same Reaction Conditions. Chem. Commun. 2017, 53, 10584–10587. 10.1039/C7CC06186B. 28895966

[ref223] Buchspies J.; Rahman M. M.; Szostak R.; Szostak M. *N*-Acylcarbazoles and *N*-Acylindoles: Electronically Activated Amides for N–C(O) Cross-Coupling by N_lp_ to Ar Conjugation Switch. Org. Lett. 2020, 22, 4703–4709. 10.1021/acs.orglett.0c01488. 32476426

[ref224] Diebolt O.; Jurčík V.; Correa da Costa R.; Braunstein P.; Cavallo L.; Nolan S. P.; Slawin A. M. Z.; Cazin C. S. J. Mixed Phosphite/N-Heterocyclic Carbene Complexes: Synthesis, Characterization and Catalytic Studies. Organometallics 2010, 29, 1443–1450. 10.1021/om9011196.

[ref225] Chen M.-T.; Vicic D. A.; Turner M. L.; Navarro O. (N-Heterocyclic Carbene)PdCl_2_(TEA) Complexes: Studies on the Effect of the “Throw-Away” Ligand in Catalytic Activity. Organometallics 2011, 30, 5052–5056. 10.1021/om200699p.

[ref226] Tang Y.-Q.; Lu J.-M.; Shao L.-X. J. NHC–Pd(II)–Im (NHC = *N*-Heterocyclic Carbene; Im = 1-Methylimidazole) Complexes as Efficient Catalysts for Suzuki–Miyaura Coupling Reactions of Aryl Chlorides. J. Organomet. Chem. 2011, 696, 3741–3744. 10.1016/j.jorganchem.2011.08.042.

[ref227] Zhou X.-X.; Shao L.-X. N-Heterocyclic Carbene/Pd(II)/1-Methylimidazole Complex Catalyzed Suzuki–Miyaura Coupling Reaction of Aryl Chlorides in Water. Synthesis 2011, 2011, 3138–3142. 10.1055/s-0030-1260169.

[ref228] Farmer J. L.; Pompeo M.; Lough A. J.; Organ M. G. [(IPent)PdCl_2_(morpholine)]: A Readily Activated Precatalyst for Room-Temperature, Additive-Free Carbon–Sulfur Coupling. Chem.–Eur. J. 2014, 20, 15790–15798. 10.1002/chem.201404705. 25303733

[ref229] Eckert P.; Sharif S.; Organ M. G. Salt to Taste: The Critical Roles Played by Inorganic Salts in Organozinc Formation and in the Negishi Reaction. Angew. Chem., Int. Ed. 2021, 60, 12224–12241. 10.1002/anie.202010917. 32986262

[ref230] Diner C.; Organ M. G. What Industrial Chemists Want—Are Academics Giving It to Them?. Organometallics 2019, 38, 66–75. 10.1021/acs.organomet.8b00818.

[ref231] Lombardi C.; Rucker R. P.; Froese R. D. J.; Sharif S.; Champagne P. A.; Organ M. G. Rate and Computational Studies for Pd-NHC-Catalyzed Amination with Primary Alkylamines and Secondary Anilines: Rationalizing Selectivity for Monoarylation versus Diarylation with NHC Ligands. Chem.—Eur. J. 2019, 25, 14223–14229. 10.1002/chem.201903362. 31593345

[ref232] Valente C.; Pompeo M.; Sayah M.; Organ M. G. Carbon–Heteroatom Coupling Using Pd-PEPPSI Complexes. Org. Process Res. Dev. 2014, 18, 180–190. 10.1021/op400278d.

[ref233] Valente C.; Çalimsiz S.; Hoi K. H.; Mallik D.; Sayah M.; Organ M. G. The Development of Bulky Palladium NHC Complexes for the Most-Challenging Cross-Coupling Reactions. Angew. Chem., Int. Ed. 2012, 51, 3314–3332. 10.1002/anie.201106131. 22287485

[ref234] Zhao Q.; Meng G.; Nolan S. P.; Szostak M. N-Heterocyclic Carbene Complexes in C–H Activation Reactions. Chem. Rev. 2020, 120, 1981–2048. 10.1021/acs.chemrev.9b00634. 31967451 PMC7241961

[ref235] Fitton P.; Johnson M. P.; McKeon J. E. Oxidative Additions to Palladium(0). J. Chem. Soc., Chem. Commun. 1968, 1968, 6–7. 10.1039/C19680000006.

[ref236] De Graaf W.; Boersma J.; Smeets W. J. J.; Spek A. L.; van Koten G. Dimethyl(*N,N,N’,N’*-tetramethylethanediamine)palladium(II) and Dimethyl[1,2-bis(dimethylphosphino)-ethane]palladium(II): Syntheses, X-ray Crystal Structures, and Thermolysis, Oxidative-Addition and Ligand-Exchange Reactions. Organometallics 1989, 8, 2907–2917. 10.1021/om00114a028.

[ref237] Markies B. A.; Canty A. J.; de Graaf W.; Boersma J.; Janssen M. D.; Hogerheide M. P.; Smeets W. J.; Spek A. L.; van Koten G. Synthesis and Structural Studies of Phenyl(iodo)- and Methyl(phenyl)Palladium(II) Complexes of Bidentate Nitrogen Donor Ligands. J. Organomet. Chem. 1994, 482, 191–199. 10.1016/0022-328X(94)88201-0.

[ref238] Markies B. A.; Canty A. J.; Boersma J.; van Koten G. Phenylpalladium(IV) Chemistry: Selectivity in Reductive Elimination from Palladium(IV) Complexes and Alkyl Halide Transfer from Palladium(IV) to Palladium(II). Organometallics 1994, 13, 2053–2058. 10.1021/om00017a071.

[ref239] Milstein D.; Stille J. K. A General, Selective, and Facile Method for Ketone Synthesis from Acid Chlorides and Organotin Compounds Catalyzed by Palladium. J. Am. Chem. Soc. 1978, 100, 3636–3638. 10.1021/ja00479a077.

[ref240] Milstein D.; Stille J. K. Palladium-Catalyzed Coupling of Tetraorganotin Compounds with Aryl and Benzyl Halides. Synthetic Utility and Mechanism. J. Am. Chem. Soc. 1979, 101, 4992–4998. 10.1021/ja00511a032.

[ref241] Scott W. J.; Stille J. K. Palladium-Catalyzed Coupling of Vinyl Triflates with Organostannanes. Synthetic and Mechanistic Studies. J. Am. Chem. Soc. 1986, 108, 3033–3040. 10.1021/ja00271a037.

[ref242] Echavarren A. M.; Stille J. K. Palladium-Catalyzed Coupling of Aryl Triflates with Organostannanes. J. Am. Chem. Soc. 1987, 109, 5478–5486. 10.1021/ja00252a029.

[ref243] Schoenberg A.; Heck R. F. Palladium-Catalyzed Amidation of Aryl, Heterocyclic, and Vinylic Halides. J. Org. Chem. 1974, 39, 3327–3331. 10.1021/jo00937a004.

[ref244] Schoenberg A.; Bartoletti I.; Heck R. F. Palladium-Catalyzed Carboalkoxylation of Aryl, Benzyl, and Vinylic Halides. J. Org. Chem. 1974, 39, 3318–3326. 10.1021/jo00937a003.

[ref245] Moser W. R.; Wang A. W.; Kildahl N. K. Mechanistic Studies of the Palladium-Catalyzed Reaction of Methanol with Bromobenzene and CO to Produce Methyl Benzoate. 1. Stoichiometric Study. J. Am. Chem. Soc. 1988, 110, 2816–2820. 10.1021/ja00217a020.

[ref246] Wallow T. I.; Goodson F. E.; Novak B. M. New Methods for the Synthesis of ArPdL_2_I (L = Tertiary Phosphine) Complexes. Organometallics 1996, 15, 3708–3716. 10.1021/om9602567.

[ref247] Yokoyama A.; Suzuki H.; Kubota Y.; Ohuchi K.; Higashimura H.; Yokozawa T. Chain-Growth Polymerization for the Synthesis of Polyfluorene via Suzuki–Miyaura Coupling Reaction from an Externally Added Initiator Unit. J. Am. Chem. Soc. 2007, 129, 7236–7237. 10.1021/ja070313v. 17506555

[ref248] Watson D. A.; Su M.; Teverovskiy G.; Zhang Y.; García- Fortanet J.; Kinzel T.; Buchwald S. L. Formation of ArF from LPdAr(F): Catalytic Conversion of Aryl Triflates to Aryl Fluorides. Science 2009, 325, 1661–1664. 10.1126/science.1178239. 19679769 PMC3038120

[ref249] Lundgren R. J.; Peters B. D.; Alsabeh P. G.; Stradiotto M. A P,N-Ligand for Palladium-Catalyzed Ammonia Arylation: Coupling of Deactivated Aryl Chlorides, Chemoselective Arylations, and Room Temperature Reactions. Angew. Chem., Int. Ed. 2010, 49, 4071–4074. 10.1002/anie.201000526. 20437437

[ref250] For related precatalysts, see Takahashi R.; Kubota K.; Ito H. Air- and Moisture-Stable Xantphos-Ligated Palladium Dialkyl Complex as a Precatalyst for Cross-Coupling Reactions. Chem. Commun. 2020, 56, 407–410. 10.1039/C9CC06946A. 31821394

[ref251] Ingoglia B. T.; Buchwald S. L. Oxidative Addition Complexes as Precatalysts for Cross-Coupling Reactions Requiring Extremely Bulky Biarylphosphine Ligands. Org. Lett. 2017, 19, 2853–2856. 10.1021/acs.orglett.7b01082. 28498667 PMC5580394

[ref252] Dennis J. M.; White N. A.; Liu R. Y.; Buchwald S. L. Breaking the Base Barrier: An Electron-Deficient Palladium Catalyst Enables the Use of a Common Soluble Base in C–N Coupling. J. Am. Chem. Soc. 2018, 140, 4721–4725. 10.1021/jacs.8b01696. 29529363 PMC5894476

[ref253] Baumgartner L. M.; Dennis J. M.; White N. A.; Buchwald S. L.; Jensen K. F. Use of a Droplet Platform to Optimize Pd-Catalyzed C–N Coupling Reactions Promoted by Organic Bases. Org. Process Res. Dev. 2019, 23, 1594–1601. 10.1021/acs.oprd.9b00236.

[ref254] McCann S. D.; Reichert E. C.; Arrechea P. L.; Buchwald S. L. Development of an Aryl Amination Catalyst with Broad Scope Guided by Consideration of Catalyst Stability. J. Am. Chem. Soc. 2020, 142, 15027–15037. 10.1021/jacs.0c06139. 32786769 PMC8057821

[ref255] Xu J.; Liu R. Y.; Yeung C. S.; Buchwald S. L. Monophosphine Ligands Promote Pd-Catalyzed C–S Cross-Coupling Reactions at Room Temperature with Soluble Bases. ACS Catal. 2019, 9, 6461–6466. 10.1021/acscatal.9b01913. 31929949 PMC6953912

[ref256] Uehling M. R.; King R. P.; Krska S. W.; Cernak T.; Buchwald S. L. Pharmaceutical Diversification via Palladium Oxidative Addition Complexes. Science 2019, 363, 405–408. 10.1126/science.aac6153. 30679373

[ref257] Chen L.; Francis H.; Carrow B. P. An “On-Cycle” Precatalyst Enables Room-Temperature Polyfluoroarylation Using Sensitive Boronic Acids. ACS Catal. 2018, 8, 2989–2994. 10.1021/acscatal.8b00341. PMC12308727 40740548

[ref258] Chen L.; Sanchez D. R.; Zhang B.; Carrow B. P. Cationic” Suzuki–Miyaura Coupling with Acutely Base-Sensitive Boronic Acids. J. Am. Chem. Soc. 2017, 139, 12418–12421. 10.1021/jacs.7b07687. 28862445

[ref259] Lau S. H.; Yu P.; Chen L.; Madsen-Duggan C. B.; Williams M. J.; Carrow B. P. Aryl Amination Using Soluble Weak Base Enabled by a Water-Assisted Mechanism. J. Am. Chem. Soc. 2020, 142, 20030–20039. 10.1021/jacs.0c09275. 33179489 PMC7690001

[ref260] Fuentes-Rivera J. J.; Zick M. E.; Düfert M. A.; Milner P. J. Overcoming Halide Inhibition of Suzuki–Miyaura Couplings with Biaryl Monophosphine-Based Catalysts. Org. Process Res. Dev. 2019, 23, 1631–1637. 10.1021/acs.oprd.9b00255.

[ref261] Vinogradova E. V.; Zhang C.; Spokoyny A. M.; Pentelute B. L.; Buchwald S. L. Organometallic Palladium Reagents for Cysteine Bioconjugation. Nature 2015, 526, 687–691. 10.1038/nature15739. 26511579 PMC4809359

[ref262] Rojas A. J.; Pentelute B. L.; Buchwald S. L. Water-Soluble Palladium Reagents for Cysteine *S*-Arylation under Ambient Aqueous Conditions. Org. Lett. 2017, 19, 4263–4266. 10.1021/acs.orglett.7b01911. 28777001 PMC5818991

[ref263] Rojas A. J.; Zhang C.; Vinogradova E. V.; Buchwald N. H.; Reilly J.; Pentelute B. L.; Buchwald S. L. Divergent Unprotected Peptide Macrocyclisation by Palladium-Mediated Cysteine Arylation. Chem. Sci. 2017, 8, 4257–4263. 10.1039/C6SC05454D. 29081961 PMC5635729

[ref264] Kondasinghe T. D.; Saraha H. Y.; Odeesho S. B.; Stockdill J. L. Direct Palladium-Mediated On-Resin Disulfide Formation from Allocam Protected Peptides. Org. Biomol. Chem. 2017, 15, 2914–2918. 10.1039/C7OB00536A. 28327729 PMC5475270

[ref265] Lee H. G.; Lautrette G.; Pentelute B. L.; Buchwald S. L. Palladium-Mediated Arylation of Lysine in Unprotected Peptides. Angew. Chem., Int. Ed. 2017, 56, 3177–3181. 10.1002/anie.201611202. PMC5741856 28206688

[ref266] Kubota K.; Dai P.; Pentelute B. L.; Buchwald S. L. Palladium Oxidative Addition Complexes for Peptide and Protein Cross-linking. J. Am. Chem. Soc. 2018, 140, 3128–3133. 10.1021/jacs.8b00172. 29406701 PMC5831526

[ref267] Roy A. H.; Hartwig J. F. Reductive Elimination of Aryl Halides from Palladium(II). J. Am. Chem. Soc. 2001, 123, 1232–1233. 10.1021/ja0034592. 11456679

[ref268] Roy A. H.; Hartwig J. F. Directly Observed Reductive Elimination of Aryl Halides from Monomeric Arylpalladium(II) Halide Complexes. J. Am. Chem. Soc. 2003, 125, 13944–13945. 10.1021/ja037959h. 14611215

[ref269] Roy A. H.; Hartwig J. F. Reductive Elimination of Aryl Halides upon Addition of Hindered Alkylphosphines to Dimeric Arylpalladium(II) Halide Complexes. Organometallics 2004, 23, 1533–1541. 10.1021/om034277u.

[ref270] Alcazar-Roman L. M.; Hartwig J. F. Mechanism of Aryl Chloride Amination: Base-Induced Oxidative Addition. J. Am. Chem. Soc. 2001, 123, 12905–12906. 10.1021/ja016491k. 11749551

[ref271] Cornils B.; Herrmann W. A., Eds. Aqueous-Phase Organometallic Catalysis: Concepts and Applications, 2nd ed.; Wiley-VCH: Weinheim, 2004.

[ref272] Herrmann W. A.; Kohlpaintner C. W. Water-Soluble Ligands, Metal Complexes, and Catalysts: Synergism of Homogeneous and Heterogeneous Catalysis. Angew. Chem., Int. Ed. 1993, 32, 1524–1544. 10.1002/anie.199315241.

[ref273] Schaper L.-A.; Hock S. J.; Herrmann W. A.; Kühn F. E. Synthesis and Application of Water-Soluble NHC Transition-Metal Complexes. Angew. Chem., Int. Ed. 2013, 52, 270–289. 10.1002/anie.201205119. 23143709

[ref274] Kitanosono T.; Masuda K.; Xu P.; Kobayashi S. Catalytic Organic Reactions in Water toward Sustainable Society. Chem. Rev. 2018, 118, 679–746. 10.1021/acs.chemrev.7b00417. 29218984

[ref275] Zhong R.; Pöthig A.; Feng Y.; Riener K.; Herrmann W. A.; Kühn F. E. Facile-Prepared Sulfonated Water-Soluble PEPPSI-Pd-NHC Catalysts for Aerobic Aqueous Suzuki–Miyaura Cross-Coupling Reactions. Green Chem. 2014, 16, 4955–4962. 10.1039/C4GC00986J.

[ref276] Borah D.; Saha B.; Sarma B.; Das P. A New PEPPSI Type N-Heterocyclic Carbene Palladium(II) Complex and Its Efficiency as a Catalyst for Mizoroki-Heck Cross-Coupling Reactions in Water. J. Chem. Sci. 2020, 132, 51 10.1007/s12039-020-1754-y.

[ref277] Lipshutz B. H.; Ghorai S. Transition-Metal-Catalyzed Cross-Couplings Going Green: in Water at Room Temperature. Aldrichimica Acta 2008, 41, 59–72. PMC3691853 23807816

[ref278] Lipshutz B. H.; Ghorai S. Designer-Surfactant-Enabled Cross-Couplings in *Water* at Room Temperature. Aldrichimica Acta 2012, 45, 3–16. 23807816 PMC3691853

[ref279] Sharma S.; Buchbinder N. W.; Braje W. M.; Handa S. Fast Amide Couplings in Water: Extraction, Column Chromatography, and Crystallization Not Required. Org. Lett. 2020, 22, 5737–5740. 10.1021/acs.orglett.0c01676. 32574062

[ref280] Handa S.; Andersson M. P.; Gallou F.; Reilly J.; Lipshutz B. H. HandaPhos: A General Ligand Enabling Sustainable ppm Levels of Palladium-Catalyzed Cross-Couplings in Water at Room Temperature. Angew. Chem., Int. Ed. 2016, 55, 4914–4918. 10.1002/anie.201510570. PMC4966530 26924396

[ref281] Takale B. S.; Thakore R. R.; Handa S.; Gallou F.; Reilly J.; Lipshutz B. H. A New, Substituted Palladacycle for ppm Level Pd-Catalyzed Suzuki–Miyaura Cross Couplings in Water. Chem. Sci. 2019, 10, 8825–8831. 10.1039/C9SC02528F. 31803456 PMC6849884

[ref282] Andersson M. P.; Gallou F.; Klumphu P.; Takale B. S.; Lipshutz B. H. Structure of Nanoparticles Derived from Designer Surfactant TPGS-750-M in Water, As Used in Organic Synthesis. Chem.—Eur. J. 2018, 24, 6778–6786. 10.1002/chem.201705524. 29504665

[ref283] De Martino M. T.; Abdelmohsen L. K. E. A.; Rutjes F. P. J. T.; van Hest J. C. M. Nanoreactors for Green Catalysis. Beil. J. Org. Chem. 2018, 14, 716–733. 10.3762/bjoc.14.61. PMC5905268 29719570

[ref284] Sheldon R. A. The E Factor 25 Years On: The Rise of Green Chemistry and Sustainability. Green Chem. 2017, 19, 18–43. 10.1039/C6GC02157C.

[ref285] Lipshutz B. H.; Isley N. A.; Fennewald J. C.; Slack E. D. On the Way Towards Greener Transition-Metal-Catalyzed Processes as Quantified by E Factors. Angew. Chem., Int. Ed. 2013, 52, 10952–10958. 10.1002/anie.201302020. PMC4068147 24030905

[ref286] Thayer A. M. Trace Metals Debate. Chem. Eng. News 2013, 91 ( (August 19), ) https://cen.acs.org/articles/91/i33/Trace-Metals-Debate.html (accessed 2022-06-28).

[ref287] Phillips S.; Holdsworth D.; Kauppinen P.; Mac Namara C. Palladium Impurity Removal from Active Pharmaceutical Ingredient Process Streams. Johnson Matthey Technol. Rev. 2016, 60, 277–286. 10.1595/205651316X693247.

[ref288] U.S. Food and Drug Administration. Q3D(R1) Elemental Impurities Guidance for Industry, Revision 1; U.S. Food and Drug Administration, March 2020; https://www.fda.gov/media/135956/download (accessed 2020-08-18).

[ref289] The HandaPhos and EvanPhos ligands are available from www.sigmaaldrich.com (accessed 2020-08-18).

[ref290] EvanPhos, a modified SPhos ligand, is relatively easy to prepare compared to HandaPhos, the synthesis of which involves a multistep process. EvanPhos was first reported by Landstrom E. B.; Handa S.; Aue D. H.; Gallou F.; Lipshutz B. H. EvanPhos: A Ligand for ppm Level Pd-Catalyzed Suzuki–Miyaura Couplings in Either Organic Solvent or Water. Green Chem. 2018, 20, 3436–3443. 10.1039/C8GC01356J.

[ref291] Thakore R. R.; Takale B. S.; Gallou F.; Reilly J.; Lipshutz B. H. *N,C*-Disubstituted Biarylpalladacycles as Precatalysts for ppm Pd-Catalyzed Cross Couplings in Water under Mild Conditions. ACS Catal. 2019, 9, 11647–11657. 10.1021/acscatal.9b04204.

[ref292] Zhang Y.; Takale B. S.; Gallou F.; Reilly J.; Lipshutz B. H. Sustainable ppm Level Palladium-Catalyzed Aminations in Nanoreactors under Mild, Aqueous Conditions. Chem. Sci. 2019, 10, 10556–10561. and references therein 10.1039/C9SC03710A. 32110341 PMC7020654

[ref293] Bacsa I.; Szemerédi D.; Wölfling J.; Schneider G.; Fekete L.; Mernyák E. The First Pd-catalyzed Buchwald–Hartwig Aminations at C-2 or C-4 in the Estrone Series. Beil. J. Org. Chem. 2018, 14, 998–1003. 10.3762/bjoc.14.85. PMC6009172 29977371

[ref294] Brals J.; Smith J. D.; Ibrahim F.; Gallou F.; Handa S. Micelle-Enabled Palladium Catalysis for Convenient sp^2^–sp^3^ Coupling of Nitroalkanes with Aryl Bromides in Water under Mild Conditions. ACS Catal. 2017, 7, 7245–7250. 10.1021/acscatal.7b02663.

[ref295] Parry P. R.; Wang C.; Batsanov A. S.; Bryce M. R.; Tarbit B. Functionalized Pyridylboronic Acids and Their Suzuki Cross-Coupling Reactions to Yield Novel Heteroarylpyridines. J. Org. Chem. 2002, 67, 7541–7543. 10.1021/jo020388b. 12375993

[ref296] Tagata T.; Nishida M. Palladium Charcoal-Catalyzed Suzuki–Miyaura Coupling to Obtain Arylpyridines and Arylquinolines. J. Org. Chem. 2003, 68, 9412–9415. 10.1021/jo034970r. 14629166

[ref297] Navarro O.; Marion N.; Mei J.; Nolan S. P. Rapid Room Temperature Buchwald–Hartwig and Suzuki–Miyaura Couplings of Heteroaromatic Compounds Employing Low Catalyst Loadings. Chem.—Eur. J. 2006, 12, 5142–5148. 10.1002/chem.200600283. 16628762

[ref298] Handa S.; Ibrahim F.; Ansari T. N.; Gallou F. π-Allylpalladium Species in Micelles of FI-750-M for Sustainable and General Suzuki–Miyaura Couplings of Unactivated Quinoline Systems in Water. ChemCatChem. 2018, 10, 4229–4233. 10.1002/cctc.201800958.

[ref299] Bihani M.; Ansari T. N.; Finck L.; Bora P. P.; Jasinski J. B.; Pavuluri B.; Leahy D. K.; Handa S. Scalable α-Arylation of Nitriles in Aqueous Micelles using Ultrasmall Pd Nanoparticles: Surprising Formation of Carbanions in Water. ACS Catal. 2020, 10, 6816–6821. 10.1021/acscatal.0c01196.

[ref300] Kapferer T.; Gong B.; Davis M. C.; Zheng X.; Har D. Chemical Process for Preparing Pyrimidine Derivatives and Intermediates Thereof. World Patent WO2016138648, September 9, 2016.

[ref301] See 4-Pyridinylboronic Acid; MilliporeSigma, 2022; https://www.sigmaaldrich.com/US/en/product/aldrich/634492 (accessed 2022-07-28).

[ref302] Park B.; Nam J. H.; Kim J. H.; Kim H. J.; Onnis V.; Balboni G.; Lee K.-T.; Park J. H.; Catto M.; Carotti A.; et al. 3,4-Dihydroquinazoline Derivatives Inhibit the Activities of Cholinesterase Enzymes. Bioorg. Med. Chem. Lett. 2017, 27, 1179–1185. 10.1016/j.bmcl.2017.01.068. 28189420

[ref303] Humphrey G. R.; Dalby S. M.; Andreani T.; Xiang B.; Luzung M. R.; Song Z. J.; Shevlin M.; Christensen M.; Belyk K. M.; Tschaen D. M. Asymmetric Synthesis of Letermovir Using a Novel Phase-Transfer-Catalyzed Aza-Michael Reaction. Org. Process Res. Dev. 2016, 20, 1097–1103. 10.1021/acs.oprd.6b00076.

[ref304] Vaswani R. G.; Albrecht B. K.; Audia J. E.; Côté A.; Dakin L. A.; Duplessis M.; Gehling V. S.; Harmange J.-C.; Hewitt M. C.; Leblanc Y.; et al. A Practical Synthesis of Indoles via a Pd-Catalyzed C–N Ring Formation. Org. Lett. 2014, 16, 4114–4117. 10.1021/ol5018118. 25068576

[ref305] Vaswani R. G.; Gehling V. S.; Dakin L. A.; Cook A. S.; Nasveschuk C. G.; Duplessis M.; Iyer P.; Balasubramanian S.; Zhao F.; Good A. C.; et al. Identification of (*R*)-*N*-((4-Methoxy-6-methyl-2-oxo-1,2-dihydropyridin-3-yl)methyl)-2-methyl-1-(1-(1-(2,2,2-trifluoroethyl)piperidin-4-yl)-ethyl)-1*H*-indole-3-carboxamide (CPI-1205), a Potent and Selective Inhibitor of Histone Methyl-transferase EZH2, Suitable for Phase I Clinical Trials for B-Cell Lymphomas. J. Med. Chem. 2016, 59, 9928–9941. 10.1021/acs.jmedchem.6b01315. 27739677 PMC5451150

[ref306] Mortimore M.; Young S. C.; Everitt S. R. L.; Knegtel R.; Pinder J. L.; Rutherford A. P.; Durrant S.; Brenchley G.; Charrier J. D.; O’Donnell M. 5-Cyano-4-(pyrrolo[2,3-*b*]pyridine-3-yl)pyrimidine Derivatives Useful as Protein Kinase Inhibitors. World Patent Appl. WO2008079346 A1, July 3, 2008.

[ref307] DeRatt L. G.; Lawson E. C.; Kumar K.; Hwang S. S.; DesJarlais R. L.; Kuduk S. D. Tandem Suzuki Coupling/Intramolecular Oxetane Ring Opening to Form Polycyclic Ring Systems. Org. Lett. 2020, 22, 5828–5832. 10.1021/acs.orglett.0c01899. 32702238

[ref308] Stumpf A.; McClory A.; Yajima H.; Segraves N.; Angelaud R.; Gosselin F. Development of an Efficient, Safe, and Environmentally Friendly Process for the Manufacture of GDC-0084. Org. Process Res. Dev. 2016, 20, 751–759. 10.1021/acs.oprd.6b00011.

[ref309] Heffron T. P.; McClory A.; Stumpf A. The Discovery and Process Chemistry Development of GDC-0084, a Brain Penetrating Inhibitor of PI3K and mTOR. ACS Symp. Ser. 2016, 1239, 147–173. 10.1021/bk-2016-1239.ch006.

[ref310] Stumpf A.; Angelaud R.; McClory A.; Yajima H.; Ndubaku C.; Olivero A. Process for the Preparation of Tricyclic PI3K Inhibitor Compounds and Methods for Using the Same for the Treatment of Cancer. World Patent WO2017106647 A1, June 22, 2017.

[ref311] Tom N.; Pfeiffer M.; Andersen D.; Gao Q. Polymorphs of (*R*)-*N*-(5-(5-Ethyl-1,2,4-oxadiazol-3-yl)-2,3-dihydro-1*H*-inden-1-yl)-1-methyl-1*H*-pyrazole-4-carboxamide. World Patent Appl. WO2021011807 A1, January 21, 2021.

[ref312] Barlaam B. C.; O’Donovan D. H.; Hughes S. J.; Moss T. A.; Nissink J. W. M.; Scott J. S.; Yang B. Chemical Compounds. U.S. Patent Appl. US20200239467 A1, July 30, 2020.

[ref313] Moniz G.; Sanders K.; Chanda A.; Yoshida K. Crystalline FGFR4 Inhibitor Compound and Uses Thereof. U.S. Patent Appl. US20200317645 A1, October 8, 2020.

[ref314] Walshe N.; Pointon H.; Nikbin N. Methods for Production of Emodepside from PF1022A Derivatives. World Patent WO2019040589, February 28, 2019.

[ref315] Finlay M. R. V.; Goldberg F. W.; Ting A. K. T. Amino-triazolopyridine Compounds and Their Use in Treating Cancer. World Patent Appl. WO2018114999 A1, June 28, 2018.

[ref316] Brohm D.; Heroult M.; Collin M.-P.; Hübsch W.; Lobell M.; Lustig K.; Grünewald S.; Bömer U.; Vöhringer V. Disubstituted Benzothienyl-pyrrolotriazines and Their Use as FGFR Kinase Inhibitors. World Patent Appl. WO2013087578 A1, June 20, 2013.

[ref317] Beck H. P.; Jaen J. C.; Osipov M.; Powers J. P.; Reilly M. K.; Shunatona H. P.; Walker J. R.; Zibinsky M.; Balog J. A.; Williams D. K. Immunoregulatory Agents. World Patent Appl. WO2016073770 A1, May 12, 2016.

[ref318] Briere J.-F.; Laclef S.; Levacher V.; Hardouin C. Novel Method for the Synthesis of Agomelatine. World Patent Appl. WO2018051042 A1, March 22, 2018.

[ref319] Zheng X.; Zhang Y.; Fu C. Preparation Method of Filgotinib. Chinese Patent Appl. CN110878097 A1, March 13, 2020.

[ref320] Locher C. P.; Bennani Y. L.; Grillot A.-L.; O’Dowd H.; Perola E.; Le Tiran A.; Charifson P. S. Combination Therapy to Treat Mycobacterium Diseases. U.S. Patent US20140045791, February 13, 2014.

[ref321] Lonn H. R.; Connolly S.; Swallow S.; Karlsson S. P.; Aurell C.-J.; Pontén J. F.; Doyle K. J.; van De Poël A. J.; Jones G. P.; Watson D. W. Certain (2*S*)-*N*-[(1*S*)-1-Cyano-2-phenylethyl]-1,4-oxazepane-2-carboxamides as Dipeptidyl Peptidase 1 Inhibitors. U.S. Patent Appl. US20180251436 A1, September 6, 2018.

[ref322] Ozer I.; Kaftanov Y.; Simhon E.; Dushkin A.; Sheffer Dee-Noor S.; Pizem H.; Avramoff A. Preparation of Trifarotene and Intermediates and Polymorphs Thereof. World Patent Appl. WO2021119351 A1, June 17, 2021.

[ref323] Hazra S.; Johansson Seechurn C. C. C.; Handa S.; Colacot T. J. The Resurrection of Murahashi Coupling after Four Decades. ACS Catal. 2021, 11, 13188–13202. 10.1021/acscatal.1c03564.

